# Design, Synthesis, and Evaluation of Ribose-Modified Anilinopyrimidine Derivatives as EGFR Tyrosine Kinase Inhibitors

**DOI:** 10.3389/fchem.2017.00101

**Published:** 2017-11-15

**Authors:** Xiuqin Hu, Disha Wang, Yi Tong, Linjiang Tong, Xia Wang, Lili Zhu, Hua Xie, Shiliang Li, You Yang, Yufang Xu

**Affiliations:** ^1^Shanghai Key Laboratory of New Drug Design, School of Pharmacy, East China University of Science and Technology, Shanghai, China; ^2^Division of Anti-tumor Pharmacology, State Key Laboratory of Drug Research, Shanghai Institute of Materia Medica, Chinese Academy of Sciences, Shanghai, China

**Keywords:** EGFR, tyrosine kinase inhibitors, anilinopyrimidine, glycosides, carbohydrate-based drugs

## Abstract

The synthesis of a series of ribose-modified anilinopyrimidine derivatives was efficiently achieved by utilizing DBU or *t*BuOLi-promoted coupling of ribosyl alcohols with 2,4,5-trichloropyrimidine as key step. Preliminary biological evaluation of this type of compounds as new EGFR tyrosine kinase inhibitors for combating EGFR L858R/T790M mutant associated with drug resistance in the treatment of non-small cell lung cancer revealed that 3-*N*-acryloyl-5-*O*-anilinopyrimidine ribose derivative **1a** possessed potent and specific inhibitory activity against EGFR L858R/T790M over WT EGFR. Based upon molecular docking studies of the binding mode between compound **1a** and EGFR, the distance between the Michael receptor and the pyrimidine scaffold is considered as an important factor for the inhibitory potency and future design of selective EGFR tyrosine kinase inhibitors against EGFR L858R/T790M mutants.

## Introduction

Epidermal growth factor receptor (EGFR), a transmembrane protein with tyrosine kinase activity, is essential for cell growth, differentiation, migration, adhesion, and proliferation under normal physiological conditions (Gschwind et al., 2004). However, overexpression of EGFR has been associated with tumor growth and progression in a variety of cancers including non-small cell lung cancer (NSCLC), head and neck squamous cell carcinoma, and pancreatic cancer (Huang and Harari, 1999; Kris et al., 2003; Moore et al., 2007; Harrington et al., 2009). Therefore, regulation of EGFR has been deemed as an important strategy for the development of cancer therapy (Huang and Harari, 1999; Gschwind et al., 2004; Steuer et al., 2015).

**Figure 1:**
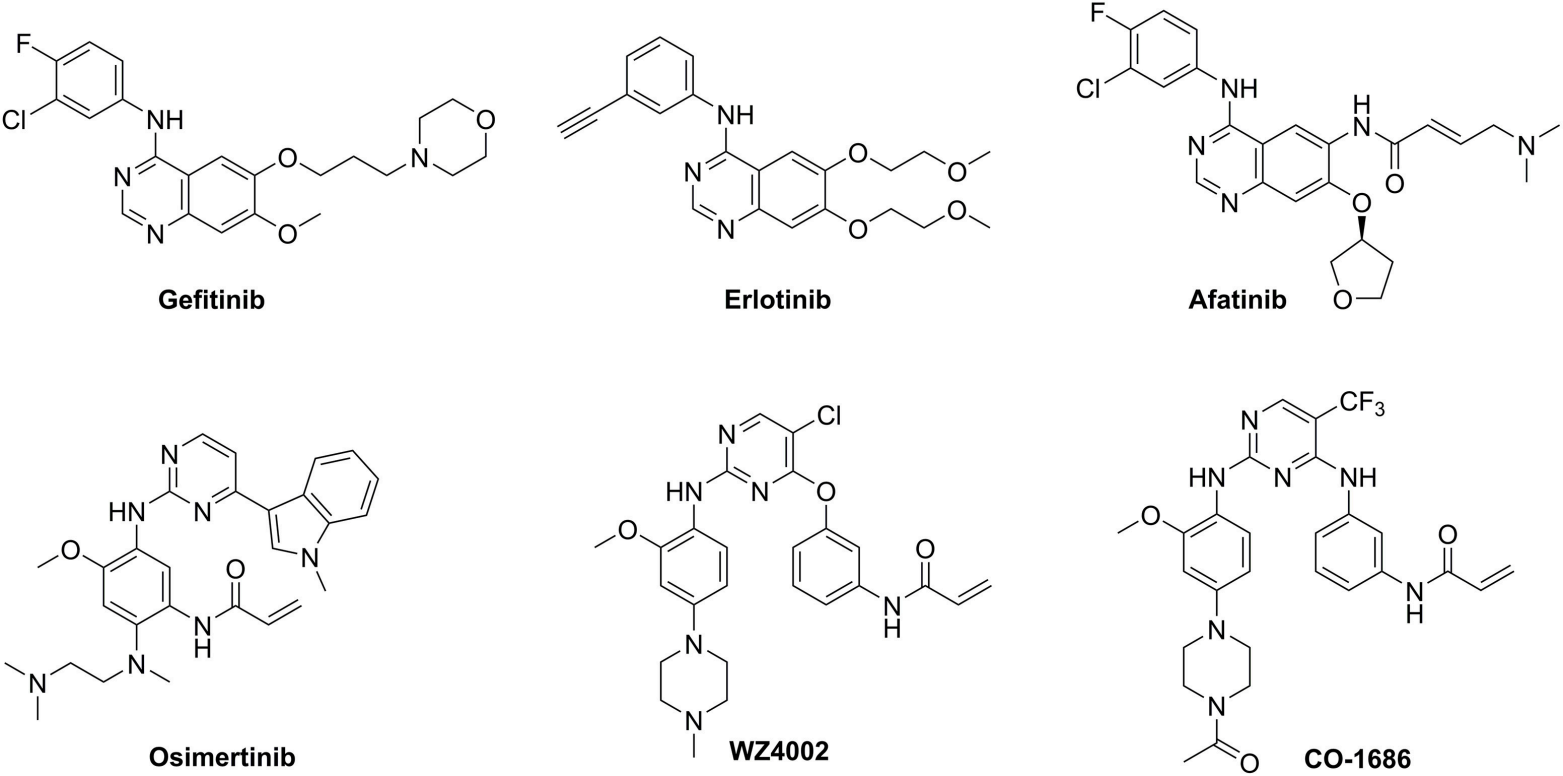
Structures of three generations of EGFR tyrosine kinase inhibitors.

First-generation EGFR tyrosine kinase inhibitors (TKIs) such as gefitinib and erlotinib that possess a 4-anilinoquinazoline scaffold, reversibly inhibit EGFR mutants (L858R and delE746_A750) as well as wild-type (WT) EGFR, resulting in significant disease control of patients with NSCLC (Figure 1) (Cohen et al., 2005; Cheng et al., 2016). However, drug resistance driven by activating mutation of the gatekeeper T790M residue in which the threonine group is replaced with the methionine moiety, greatly counteracted the clinical efficiency of first-generation TKIs against NSCLC (Ozvegy-Laczka et al., 2005; Balak et al., 2006; De Luca et al., 2008; Pao and Chmielecki, 2010). To address this issue, the irreversible EGFR TKIs (afatinib, osimertinib, WZ4002, and CO-1686) which contain a Michael acceptor moiety for binding covalently to the thiol group of Cys797 in the ATP binding domain of EGFR, were developed to treat NSCLC via the efficient inhibition of EGFR mutants (Figure 1; Castellanos and Horn, 2015). Among them, second-generation TKIs such as afatinib potently inhibited both EGFR mutants (L858R/T790M) and WT-EGFR without mutant selectivity, thereby leading to side effects such as rash and diarrhea (Dungo and Keating, 2013). In contrast, third-generation TKIs such as osimertinib, WZ4002, and CO-1686 bearing an anilinopyrimidine core, showed high potency and selectivity for EGFR L858R/T790M over WT EGFR, therefore serving as mutant-selective TKIs targeting EGFR mutants involved in NSCLC (Zhou et al., 2009; Walter et al., 2013; Cross et al., 2014; Finlay et al., 2014; Gray and Haura, 2014).

**Figure 2:**
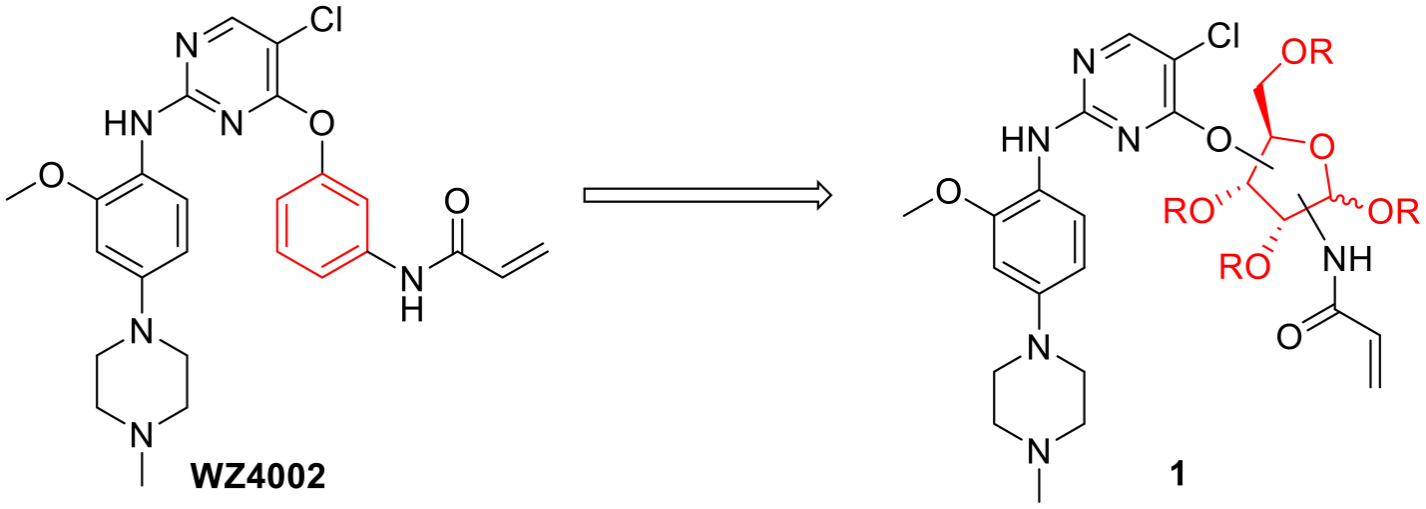
Design of novel EGFR tyrosine kinase inhibitors.

Considering the drug resistance is rapidly emerging for third-generation TKIs (Eberlein et al., 2015; Niederst et al., 2015; Piotrowska et al., 2015; Thress et al., 2015), design of EGFR inhibitors with new structural skeletons could lead to the discovery of novel types of TKIs against EGFR mutants such as the triple mutant L858R/T790M/C797S (Günther et al., 2016, 2017; Jia et al., 2016; Juchum et al., 2017; Park et al., 2017). Based on the fact that most commercially available TKIs are ATP-competitive inhibitors for binding at the catalytic domain of the EGFR tyrosine kinase (Traxler and Furet, 1999; Grünwald and Hidalgo, 2003; Normanno et al., 2003), we envisioned that replacement of the phenyl ring on the right side of WZ4002 with a chiral ribosyl moiety would provide compound **1** as a novel type of carbohydrate-based EGFR TKI against the drug resistance involved in NSCLC (Figure 2). Here we report the synthesis, preliminary biological evaluation and molecular docking studies of ribose-containing anilinopyrimidine derivatives as EGFR TKIs against EGFR L858R/T790M.

## Materials and methods

Commercial reagents were used without further purification except where noted. Solvents were dried and redistilled prior to use in the usual way. All reactions were performed in oven-dried glassware with magnetic stirring under an inert atmosphere unless noted otherwise. Analytical thin layer chromatography (TLC) was performed on precoated plates of Silica Gel (0.25–0.3 mm, Shanghai, China). The TLC plates were visualized with UV light and by staining with sulfuric acid-ethanol solution. Silica gel column chromatography was performed on Silica Gel AR (100–200 mesh, Shanghai, China). NMR spectra were measured with a Bruker Avance III 400 or Bruker Avance III 500 spectrometer. The ^1^H and ^13^C NMR spectra were calibrated against the residual proton and carbon signals of the solvents as internal references (CDCl_3_: δ_H_ = 7.26 ppm and δ_C_ = 77.2 ppm; CD_3_OD: δ_H_ = 3.31 ppm and δ_C_ = 49.0 ppm). Multiplicities are quoted as singlet (s), broad singlet (br s), doublet (d), doublet of doublets (dd), triplet (t), or multiplet (m). All NMR chemical shifts (δ) were recorded in ppm and coupling constants (*J*) were reported in Hz. Mass spectra were recorded on an Agilent Technologies 6120 or LCT Premier XE FTMS instrument.

### 1,2-*O*-isopropylidene-3-*N*-acryloyl-3-deoxy-5-*O*-(2,5-dichloropyrimidin-4-yl)-α-d-ribofuranoside 7

To a solution of compound **5** (0.60 g, 2.47 mmol) in anhydrous CH_2_Cl_2_ (30 mL) at room temperature, was added DBU (1.48 mL, 5.94 mmol) and 2,4,5-trichloropyrimidine **6** (0.57 mL, 4.94 mmol). After stirring at room temperature for 2 h, the reaction mixture was diluted with saturated aqueous NH_4_Cl, and extracted with CH_2_Cl_2_. The organic layer was washed with brine, dried over Na_2_SO_4_, and concentrated *in vacuo*. The residue was purified by silica gel chromatography (petroleum ether/EtOAc: 80/1) to give **7** (0.88 g, 92%) as a pale yellow syrup: ^1^H NMR (400 MHz, CDCl_3_) δ 8.30 (s, 1 H), 6.29 (dd, *J* = 1.2, 17.2 Hz, 1 H), 6.12 (dd, *J* = 10.4, 17.2 Hz, 1 H), 6.07 (d, *J* = 8.8 Hz, 1 H, NH), 5.90 (d, *J* = 3.6 Hz, 1 H, H-1), 5.69 (dd, *J* = 1.2, 10.4 Hz, 1 H), 4.73 (dd, *J* = 2.4, 12.0 Hz, 1 H), 4.66 (t, *J* = 4.0 Hz, 1 H), 4.61–4.55 (m, 2 H), 4.16 (m, 1 H), 1.57 (s, 3 H), 1.35 (s, 3 H); ^13^C NMR (100 MHz, CDCl_3_) δ 165.4, 165.3, 157.4, 157.2, 130.1, 128.0, 117.0, 113.0, 104.7, 79.1, 78.2, 67.3, 52.1, 26.8, 26.5; ESI-MS (ESI) m/z calcd for C_15_H_17_O_5_N_3_Cl_2_Na [M + Na]^+^ 412.0, found 412.0.

### 1,2-*O*-isopropylidene-3-*N*-acryloyl-3-deoxy-5-*O*-[5-chloro-2-*N*-(2-methoxy-4-(4-methylpiperazin-1-yl)phenyl)pyrimidin-4-yl]-α-d-ribofuranoside 1a

To a solution of compound **7** (80 mg, 0.21 mmol) and aniline derivative **8** (91 mg, 0.41 mmol) in isobutanol (3 mL), was added TFA (0.12 mL, 1.55 mmol). The mixture was heated to 100°C and stirred for 5 h. After cooling down to room temperature, the mixture was quenched with Et_3_N (3 mL) and concentrated *in vacuo* to give a residue, which was purified by silica gel column chromatography (CH_2_Cl_2_/MeOH: 30/1) to give **1a** (82 mg, 69%) as a pale yellow powder: ^1^H NMR (400 MHz, CDCl_3_) δ 8.10 (d, *J* = 8.8 Hz, 1 H), 8.08 (s, 1 H), 7.35 (br s, 1 H), 6.56 (dd, *J* = 2.4, 8.8 Hz, 1 H), 6.52 (d-like, *J* = 2.4 Hz, 1 H), 6.30 (dd, *J* = 1.2, 17.2 Hz, 1 H), 6.11 (m, 2 H), 5.91 (d, *J* = 3.6 Hz, 1 H, H-1), 5.67 (dd, *J* = 1.2, 10.4 Hz, 1 H), 4.66 (m, 2 H), 4.59–4.49 (m, 3 H), 4.22 (m, 1 H), 3.86 (s, 3 H), 3.16 (t-like, *J* = 5.2 Hz, 4 H), 2.58 (t-like, *J* = 5.2 Hz, 4 H), 2.34 (s, 3 H), 1.58 (s, 3 H), 1.35 (s, 3 H); ^13^C NMR (100 MHz, CDCl_3_) δ 165.4, 164.1, 157.9, 156.5, 149.2, 147.5, 130.2, 127.8, 122.0, 120.0, 112.9, 108.3, 106.2, 104.8, 100.6, 79.1, 78.2, 66.6, 55.8, 55.3, 52.7, 50.2, 46.2, 26.8, 26.5; HRMS (ESI) m/z calcd for C_27_H_36_O_6_N_6_ClNa [M + H]^+^ 575.2385, found 575.2385.

### 3-*N*-acryloyl-3-deoxy-5-*O*-[5-chloro-2-*N*-(2-methoxy-4-(4-methylpiperazin-1-yl)phenyl)pyrimidin-4-yl]-d-ribofuranose 1b

A solution of compound **1a** (58 mg, 0.10 mmol) in TFA/acetic acid/water (1/15/4, v/v/v, 5 mL) was stirred at 70°C for 7 h. Concentration *in vacuo* and elution through reverse phase C-18 column (H_2_O/MeOH: 2/3) provided **1b** (44 mg, 83%) as a pale yellow syrup: ^1^H NMR (400 MHz, CD_3_OD) δ 8.07 (s, 1 H), 8.05 (s, 2.4 H), 7.92 (m, 3.4 H), 6.67 (d-like, *J* = 2.4 Hz, 3.4 H), 6.58 (dd, *J* = 2.8, 8.8 Hz, 3.4 H), 6.35 (m, 3.4 H), 6.21 (m, 3.4 H), 5.67 (m, 3.4 H), 5.38 (d, *J* = 4.0 Hz, 1 H), 5.22 (s, 2.4 H), 4.67 (m, 2.4 H), 4.58 (m, 3.4 H), 4.49 (m, 4.4 H), 4.34–4.24 (m, 4.4 H), 4.00 (d-like, *J* = 4.4 Hz, 2.4 H), 3.87 (s, 10.2 H), 3.78 (m, 6.8 H), 3.58 (m, 6.8 H), 3.26 (m, 6.8 H), 3.03 (m, 6.8 H), 2.96 (s, 10.2 H); HRMS (ESI) m/z calcd for C_24_H_32_O_6_N_6_ClNa [M + H]^+^ 535.2072, found 535.2079.

### 2,3-*O*-isopropylidene-5-*O*-(2,5-dichloropyrimidin-4-yl)-α-d-ribofuranosyl acrylamide 14

To a solution of compound **13** (147 mg, 0.60 mmol) in anhydrous CH_2_Cl_2_ (25 mL) at room temperature, was added DBU (0.21 mL, 1.42 mmol) and 2,4,5-trichloropyrimidine **6** (0.12 mL, 1.07 mmol). After stirring at room temperature for 2 h, the reaction mixture was diluted with saturated aqueous NH_4_Cl, and extracted with CH_2_Cl_2_. The organic layer was washed with brine, dried over Na_2_SO_4_, and concentrated *in vacuo*. The residue was purified by silica gel chromatography (petroleum ether/EtOAc: 5/1) to give **14** (217 mg, 93%) as a colorless syrup: ^1^H NMR (400 MHz, CDCl_3_) δ 8.36 (s, 1 H), 6.58 (d, *J* = 9.2 Hz, 1 H, NH), 6.32 (dd, *J* = 1.2, 17.2 Hz, 1 H), 6.15 (dd, *J* = 10.4, 17.2 Hz, 1 H), 6.08 (dd, *J* = 4.0, 9.2 Hz, 1 H, H-1), 5.72 (dd, *J* = 1.2, 10.4 Hz, 1 H), 4.86 (d-like, *J* = 6.0 Hz, 1 H), 4.82 (dd, *J* = 4.4, 6.0 Hz, 1 H), 4.55 (m, 2 H), 4.45 (t, *J* = 2.8 Hz, 1 H), 1.57 (s, 3 H), 1.38 (s, 3 H); ^13^C NMR (100 MHz, CDCl_3_) δ 165.1, 165.0, 157.7, 157.4, 130.6, 128.2, 116.8, 113.4, 82.3, 81.6, 79.7, 79.4, 70.4, 26.4, 24.8; HRMS (ESI) m/z calcd for C_15_H_17_O_5_N_3_Cl_2_Na [M + Na]^+^ 412.0443, found 412.0448.

### 5-*O*-[5-chloro-2-*N*-(2-methoxy-4-(4-methylpiperazin-1-yl)phenyl)pyrimidin-4-yl]-d-ribofuranosyl acrylamide 1c

To a solution of compound **14** (58 mg, 0.15 mmol) and aniline derivative **8** (66 mg, 0.30 mmol) in isobutanol (3 mL), was added TFA (0.084 mL, 1.13 mmol). The mixture was heated to 100°C and stirred for 5 h. After cooling down to room temperature, the mixture was quenched with Et_3_N (3 mL) and concentrated *in vacuo* to give a residue, which was purified by silica gel column chromatography (CH_2_Cl_2_/MeOH: 30/1) to give **15** (60 mg, 70%) as a pale yellow oil: HRMS (ESI) m/z calcd for C_27_H_36_O_6_N_6_Cl [M + H]^+^ 575.2385, found 575.2383. A solution of compound **15** (85 mg, 0.15 mmol) in TFA/acetic acid/water (1/20/4, v/v/v, 4 mL) was stirred at 50°C for 5 h. Concentration *in vacuo* and elution through reverse phase C-18 column (H_2_O/MeOH: 2/3) provided **1c** (64 mg, 80%) as a pale yellow syrup. **1c** (α): ^1^H NMR (400 MHz, CD_3_OD) δ 8.06 (s, 1 H), 7.88 (d, *J* = 8.8 Hz, 1 H), 6.63 (d-like, *J* = 2.4 Hz, 1 H), 6.53 (dd, *J* = 2.8, 8.8 Hz, 1 H), 6.34 (dd, *J* = 10.0, 17.2 Hz, 1 H), 6.27 (dd, *J* = 2.0, 17.2 Hz, 1 H), 5.83 (d, *J* = 4.4 Hz, 1 H, H-1), 5.70 (dd, *J* = 2.0, 10.0 Hz, 1 H), 4.57 (dd, *J* = 3.2, 12.0 Hz, 1 H), 4.40 (dd, *J* = 4.0, 11.6 Hz, 1 H), 4.25 (m, 3 H), 3.85 (s, 3 H), 3.16 (t, *J* = 4.8 Hz, 4 H), 2.61 (t, *J* = 4.8 Hz, 4 H), 2.34 (s, 3 H); ^13^C NMR (100 MHz, CD_3_OD) δ 168.0, 165.6, 159.5, 157.3, 151.6, 148.8, 132.1, 128.1, 123.0, 122.3, 109.3, 106.7, 101.9, 81.9 (C-1), 81.5, 73.3, 71.8, 68.2, 56.4, 55.8, 50.2, 45.6; **1c** (β): ^1^H NMR (400 MHz, CD_3_OD) δ 8.05 (s, 1 H), 7.87 (d, *J* = 8.4 Hz, 1 H), 6.65 (d-like, *J* = 2.4 Hz, 1 H), 6.54 (dd, *J* = 2.4, 8.8 Hz, 1 H), 6.23 (m, 2 H), 5.67 (dd, *J* = 4.8, 6.8 Hz, 1 H), 5.48 (d, *J* = 4.4 Hz, 1 H, H-1), 4.58 (dd, *J* = 3.6, 12.0 Hz, 1 H), 4.41 (dd, *J* = 4.8, 12.0 Hz, 1 H), 4.24–4.14 (m, 2 H), 4.03 (t, *J* = 4.8 Hz, 1 H), 3.84 (s, 3 H), 3.76 (m, 2 H), 3.57 (m, 2 H), 3.23 (m, 2 H), 3.00 (m, 2 H), 2.93 (s, 3 H); HRMS (ESI) m/z calcd for C_24_H_32_O_6_N_6_Cl [M + H]^+^ 535.2072, found 535.2087.

### 5-*O*-*tert*-butyldiphenylsilyl-3-*O*-(2,5-dichloropyrimidin-4-yl)-2-*O*-*tert*-butyldimethylsilyl-β-d-ribofuranosyl azide 21

To a solution of compound **19** (0.86 g, 1.63 mmol) in anhydrous CH_2_Cl_2_ (25 mL) at room temperature, was added *t*BuOLi (1.83 g, 22.82 mmol) and 2,4,5-trichloropyrimidine **6** (0.37 mL, 3.26 mmol). After stirring under reflux for 36 h, the reaction mixture was diluted with saturated aqueous NH_4_Cl, and extracted with CH_2_Cl_2_. The organic layer was washed with brine, dried over Na_2_SO_4_, and concentrated *in vacuo*. The residue was purified by silica gel chromatography (petroleum ether/EtOAc: 80/1) to give **21** (0.88 g, 80%) as a pale yellow syrup: ^1^H NMR (400 MHz, CDCl_3_) δ 8.34 (s, 1 H), 7.71–7.67 (m, 4 H), 7.45–7.34 (m, 6 H), 5.66 (t, *J* = 4.8 Hz, 1 H, H-3), 5.25 (d, *J* = 3.6 Hz, 1 H, H-1), 4.40 (dd, *J* = 3.6, 7.6 Hz, 1 H), 4.33 (t, *J* = 4.4 Hz, 1 H), 3.91 (dd, *J* = 4.0, 11.6 Hz, 1 H), 3.84 (dd, *J* = 3.6, 11.6 Hz, 1 H), 1.09 (s, 9 H), 0.75 (s, 9 H), 0.05 (s, 3 H), −0.15 (s, 3 H); HRMS (ESI) m/z calcd for C_31_H_42_O_4_N_5_Cl_2_Si_2_ [M + H]^+^ 674.2152, found 674.2155.

### 5-*O*-*tert*-butyldiphenylsilyl-3-*O*-[5-chloro-2-*N*-(2-methoxy-4-(4-methylpiperazin-1-yl)phenyl)pyrimidin-4-yl]-2-*O*-*tert*-butyldimethylsilyl-β-d-ribofuranosyl azide 22

To a solution of compound **21** (0.53 g, 0.79 mmol) and aniline derivative **8** (0.70 g, 3.16 mmol) in isobutanol (12 mL), was added TFA (1.47 mL, 19.75 mmol). The mixture was heated to 100°C and stirred for 5 h. After cooling down to room temperature, the mixture was quenched with Et_3_N (8 mL) and concentrated *in vacuo* to give a residue, which was purified by silica gel column chromatography (CH_2_Cl_2_/MeOH: 30/1) to give **22** (0.47 g, 69%) as a white powder: ^1^H NMR (400 MHz, CDCl_3_) δ 8.12 (s, 1 H), 8.08 (d, *J* = 9.2 Hz, 1 H), 7.69 (dd, *J* = 1.6, 7.6 Hz, 2 H), 7.63 (dd, *J* = 1.6, 8.0 Hz, 2 H), 7.43–7.25 (m, 6 H), 6.54 (m, 2 H), 5.58 (dd, *J* = 4.4, 7.2 Hz, 1 H, H-3), 5.29 (d, *J* = 1.6 Hz, 1 H, H-1), 4.47 (m, 1 H), 4.38 (dd, *J* = 2.0, 4.4 Hz, 1 H), 4.02 (dd, *J* = 2.8, 11.6 Hz, 1 H), 3.88 (s, 3 H), 3.83 (dd, *J* = 3.6, 12.0 Hz, 1 H), 3.17 (t, *J* = 5.2 Hz, 4 H), 2.61 (t, *J* = 4.8 Hz, 4 H), 2.37 (s, 3 H), 1.06 (s, 9 H), 0.78 (s, 9 H), −0.07 (s, 3 H), −0.25 (s, 3 H); ^13^C NMR (100 MHz, CDCl_3_) δ 163.6, 157.9, 156.9, 149.3, 147.6, 135.8, 135.7, 133.1, 133.0, 129.9, 127.9, 127.8, 121.8, 120.1, 108.4, 106.1, 100.6, 95.8, 81.6, 74.7, 74.2, 62.8, 55.8, 55.3, 50.1, 46.3, 26.9, 25.6, 19.3, 18.0, −4.9, −5.4; HRMS (ESI) m/z calcd for C_43_H_60_O_5_N_8_ClSi_2_ [M + H]^+^ 859.3914, found 859.3920.

### 5-*O*-*tert*-butyldiphenylsilyl-3-*O*-[5-chloro-2-*N*-(2-methoxy-4-(4-methylpiperazin-1-yl)phenyl)pyrimidin-4-yl]-2-*O*-*tert*-butyldimethylsilyl-α-d-ribofuranosyl acrylamide 23

A mixture of compound **22** (190 mg, 0.22 mmol) and Pd/C (50 mg, 10%) in EtOH (7 mL) was stirred under an atmosphere of H_2_ at room temperature for overnight. The mixture was filtered through celite, washed with EtOH and concentrated *in vacuo* to afford the corresponding amine for the next step without further purification. To a solution of the resulting amine in CH_2_Cl_2_ (7 mL) at room temperature, was added DCC (69 mg, 0.33 mmol), DMAP (41 mg, 0.33 mmol), and acrylic acid (0.061 mL, 0.89 mmol). After stirring at room temperature for 4 h, the mixture was concentrated *in vacuo* to give a residue, which was purified by silica gel column chromatography (CH_2_Cl_2_/MeOH: 30/1) to afford **23** (76 mg, 39% over two steps) as a pale yellow syrup: ^1^H NMR (400 MHz, CDCl_3_) δ 8.17 (s, 1 H), 8.09 (d, *J* = 8.8 Hz, 1 H), 7.71 (m, 4 H), 7.43–7.36 (m, 6 H), 7.12 (d, *J* = 9.2 Hz, 1 H), 6.53 (d, *J* = 2.4 Hz, 1 H), 6.42 (m, 1 H), 6.36 (dd, *J* = 1.2, 16.8 Hz, 1 H), 6.14 (dd, *J* = 10.4, 17.2 Hz, 1 H), 6.02 (d-like, *J* = 4.8 Hz, 1 H), 5.98 (dd, *J* = 6.0, 9.2 Hz, 1 H), 5.70 (dd, *J* = 1.2, 10.4 Hz, 1 H), 4.72 (dd, *J* = 5.2 Hz, 1 H), 4.36 (br s, 1 H), 3.86 (m, 5 H), 3.07 (br s, 4 H), 2.54 (br s, 4 H), 2.35 (s, 3 H), 1.11 (s, 9 H), 0.68 (s, 9 H), −0.02 (s, 3 H), −0.09 (s, 3 H); ^13^C NMR (100 MHz, CDCl_3_) δ 165.9, 164.0, 157.8, 157.0, 149.3, 147.6, 135.8, 135.5, 133.4, 132.5, 131.0, 130.1, 130.0, 128.9, 128.0, 127.4, 121.5, 120.0, 108.1, 105.8, 100.4, 82.2, 80.4, 77.4, 71.0, 64.2, 55.7, 55.2, 49.9, 46.2, 27.0, 25.4, 19.5, 17.7, −5.1, −5.3; HRMS (ESI) m/z calcd for C_46_H_64_O_6_N_6_ClSi_2_ [M + H]^+^ 887.4114, found 887.4116.

### 3-*O*-[5-chloro-2-*N*-(2-methoxy-4-(4-methylpiperazin-1-yl)phenyl)pyrimidin-4-yl]-2-*O*-*tert*-butyldimethylsilyl-α-d-ribofuranosyl acrylamide 1d

To a solution of compound **23** (91 mg, 0.11 mmol) in pyridine (3 mL) at room temperature, was added HF·pyridine (0.19 mL). After stirring at room temperature for overnight, the mixture was poured into saturated aqueous NaHCO_3_ and extracted with CH_2_Cl_2_. The combined organic layers were washed with brine, dried over Na_2_SO_4_, and concentrated *in vacuo*. The residue was purified by silica gel column chromatography (CH_2_Cl_2_/MeOH: 20/1) to afford **1d** (40 mg, 68%) as a pale yellow syrup: ^1^H NMR (400 MHz, CDCl_3_) δ 8.15 (s, 1 H), 8.02 (d, *J* = 8.4 Hz, 1 H), 7.71 (m, 4 H), 7.30 (br s, 1 H), 7.06 (d, *J* = 8.4 Hz, 1 H), 6.54 (m, 2 H), 6.33 (d-like, *J* = 17.2 Hz, 1 H), 6.12 (dd, *J* = 10.0, 16.8 Hz, 1 H), 5.90 (dd, *J* = 6.0, 8.4 Hz, 1 H), 5.73 (m, 2 H), 4.51 (t, *J* = 5.6 Hz, 1 H), 4.30 (br s, 1 H), 3.87 (s, 3 H), 3.82 (d-like, *J* = 12.4 Hz, 1 H), 3.71 (d-like, *J* = 11.2 Hz, 1 H), 3.18 (br s, 4 H), 2.61 (br s, 4 H), 2.37 (s, 3 H), 0.72 (s, 9 H), −0.02 (s, 3 H), −0.10 (s, 3 H); ^13^C NMR (100 MHz, CDCl_3_) δ 166.1, 163.8, 157.9, 157.0, 149.6, 147.5, 130.9, 127.7, 121.9, 120.5, 108.6, 106.1, 100.8, 82.1, 80.6, 76.3, 70.9, 62.5, 55.8, 55.0, 49.6, 45.7, 25.5, 17.9, −5.0, −5.2; HRMS (ESI) m/z calcd for C_30_H_46_O_6_N_6_ClSi [M + H]^+^ 649.2937, found 649.2940.

### Kinase assay

Kinases domain of EGFR WT and EGFR L858R/T790M were expressed using the Bac-to-Bac™ baculo virus expression system (Invitrogen, Carlsbad, CA, USA) and purified in Ni-NTA columns (QIAGEN Inc., Valencia, CA, USA). The kinase activity was evaluated with enzyme-linked immunosorbent assay (ELISA). Briefly, 20 μg/mL Poly (Glu, Tyr) 4:1 (Sigma, St. Louis, MO) was precoated in 96-well ELISA plates as substrate. After adding 50 μL of 10 μmol/L ATP solution which was diluted in kinase reaction buffer (50 mM HEPES pH 7.4, 20 mM MgCl_2_, 0.1 mM MnCl_2_, 0.2 mM Na_3_VO_4_, 1 mM DTT), the plate was treated with 1 μL of indicated concentrations of compounds (dissolved in DMSO) per well. Experiments at each concentration were performed in duplicate. Reaction was initiated by adding tyrosine kinase diluted in kinase reaction buffer. After incubation at 37°C for 1 h, the wells were washed three times with phosphate buffered saline (PBS) containing 0.1% Tween 20 (T-PBS). One hundred microliters of anti-phosphotyrosine (PY99) antibody (1:1,000, Santa Cruz Biotechnology, Santa Cruz, CA) diluted in T-PBS containing 5 mg/mL BSA was added and the plate was incubated at 37°C for 30 min. After the plate was washed three times, 100 μL horseradish peroxidase-conjugated goat anti-mouse IgG (1:2,000, Calbiochem, SanDiego, CA) was added and the plate was incubated at 37°C for 30 min. The plate was washed, added with 100 μL citrate buffer (0.1 M, pH 5.5) containing 0.03% H_2_O_2_. Then 2 mg/mL *o*-phenylenediamine was added, and samples were incubated at room temperature until color emerged. The reaction was terminated immediately by adding 50 μL of 2 M H_2_SO_4_. Plate was read using a multiwell spectrophotometer (VERSAmax™, Molecular Devices, Sunnyvale, CA, USA) at 492 nm. The inhibitory rate (%) was calculated with the formula: [1 − (A492 treated/A492 control)] × 100%. IC_50_ values were calculated from the inhibitory curves.

### Molecular docking

EGFR^T790M/L858R^ structure (PDB: 3IKA) was retrieved from the Protein Data Bank and covalent docking was performed with maestro (Schrödinger, Inc., version 10.2). Compound **1a** was docked into the EGFR protein as an irreversible inhibitor using Covalent Docking module. The docking procedure was validated by re-docking the co-crystallized ligand WZ4002 into the ATP binding site of EGFR^T790M/L858R^ structure. The details of the docking workflow are listed below:

1. Protein was prepared using the “Protein Preparation Wizard” workflow. All water molecules were removed from the structure of the complex. Hydrogen atoms and charges were added during a brief relaxation. After optimizing the hydrogen bond network, the crystal structure was minimized using the OPLS_2005 force field with the maximum root mean square deviation (RMSD) value of 0.3 Å.
2. The ligand was prepared with LigPrep module in Maestro, including adding hydrogen atoms, ionizing at a pH range from 5.0 to 9.0, and producing the corresponding low-energy 3D structure.
3. Pose prediction mode of Covalent Docking module was adopted to dock the molecules into the ATP-binding site with the default parameters. The center of the grid box was defined with the intrinsic ligand and Michael addition reaction type was chosen. The top-ranking poses of molecule **1a** were retained.

## Result and discussion

**Scheme 1:**
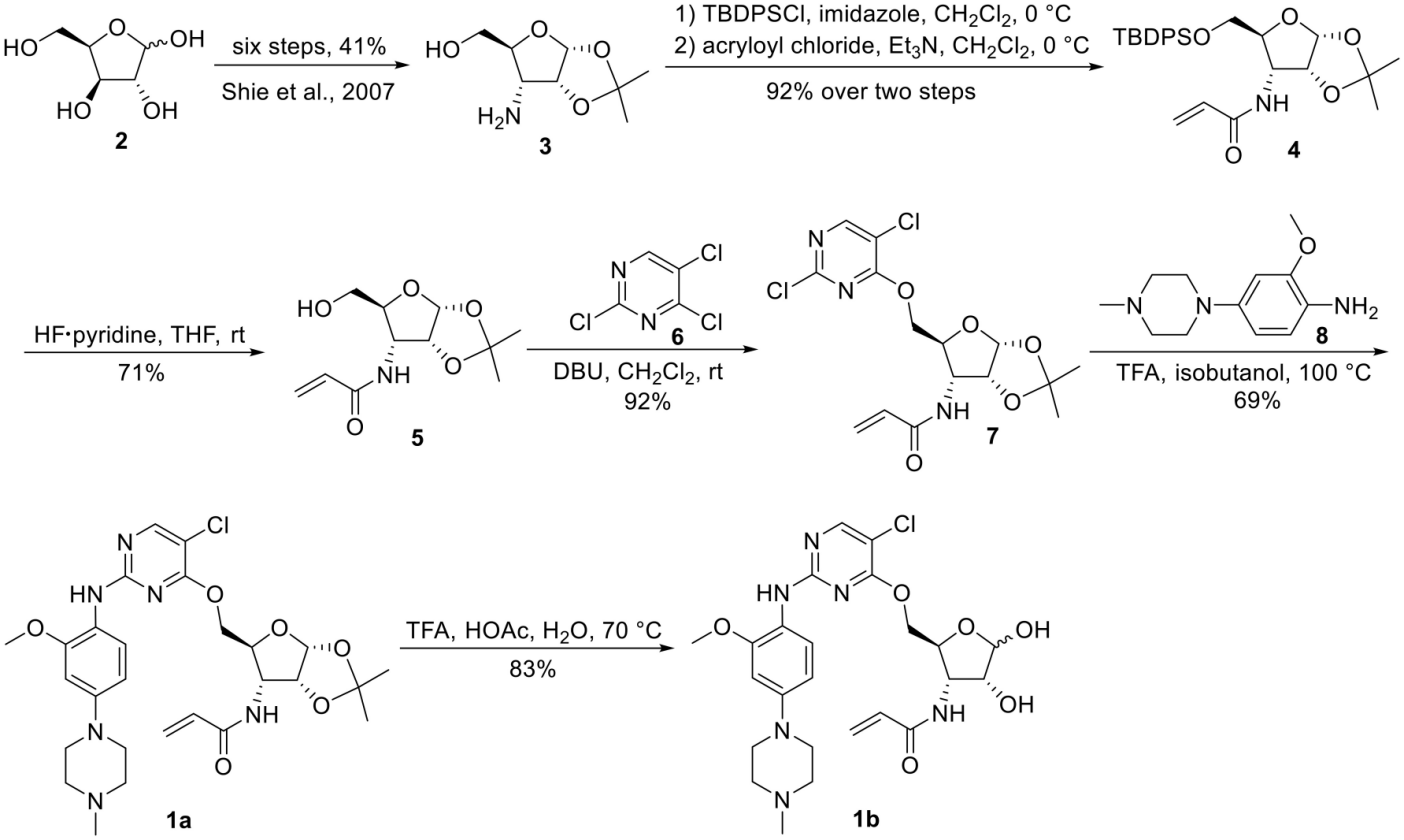
Synthesis of 3-*N*-acryloyl-5-*O*-anilinopyrimidine ribose derivatives **1a** and **1b**.

The synthesis of 3-*N*-acryloyl-5-*O*-anilinopyrimidine ribose derivatives **1a** and **1b** commenced with 3-amino ribose derivative **3** that can be readily prepared from d-xylose **2** in 41% yield over six steps (Scheme 1; Shie et al., 2007). Protection of the primary hydroxyl group in **3** with TBDPSCl followed by condensation with acryloyl chloride using Et_3_N as base gave ribose derivative **4** in 92% yield (two steps). Treatment of **4** with HF·pyridine afforded alcohol **5** in 71% yield. When **5** was reacted with 2,4,5-trichloropyrimidine **6** in the presence of K_2_CO_3_ or DIPEA, almost no desired product was observed. Exhilaratingly, nucleophilic reaction of **5** with **6** employing stronger base (DBU) as promoter proceeded smoothly to provide ribose derivative **7** in excellent yield (92%). Subjection of **7** to the known aniline derivative **8** (Han et al., 2014) under the promotion of TFA in isobutanol at 100°C delivered **1a** in 69% yield. Removal of the 1,2-*O*-isopropylidene group in **1a** with TFA in acetic acid and water at 70°C produced **1b** in 83% yield.

**Scheme 2:**
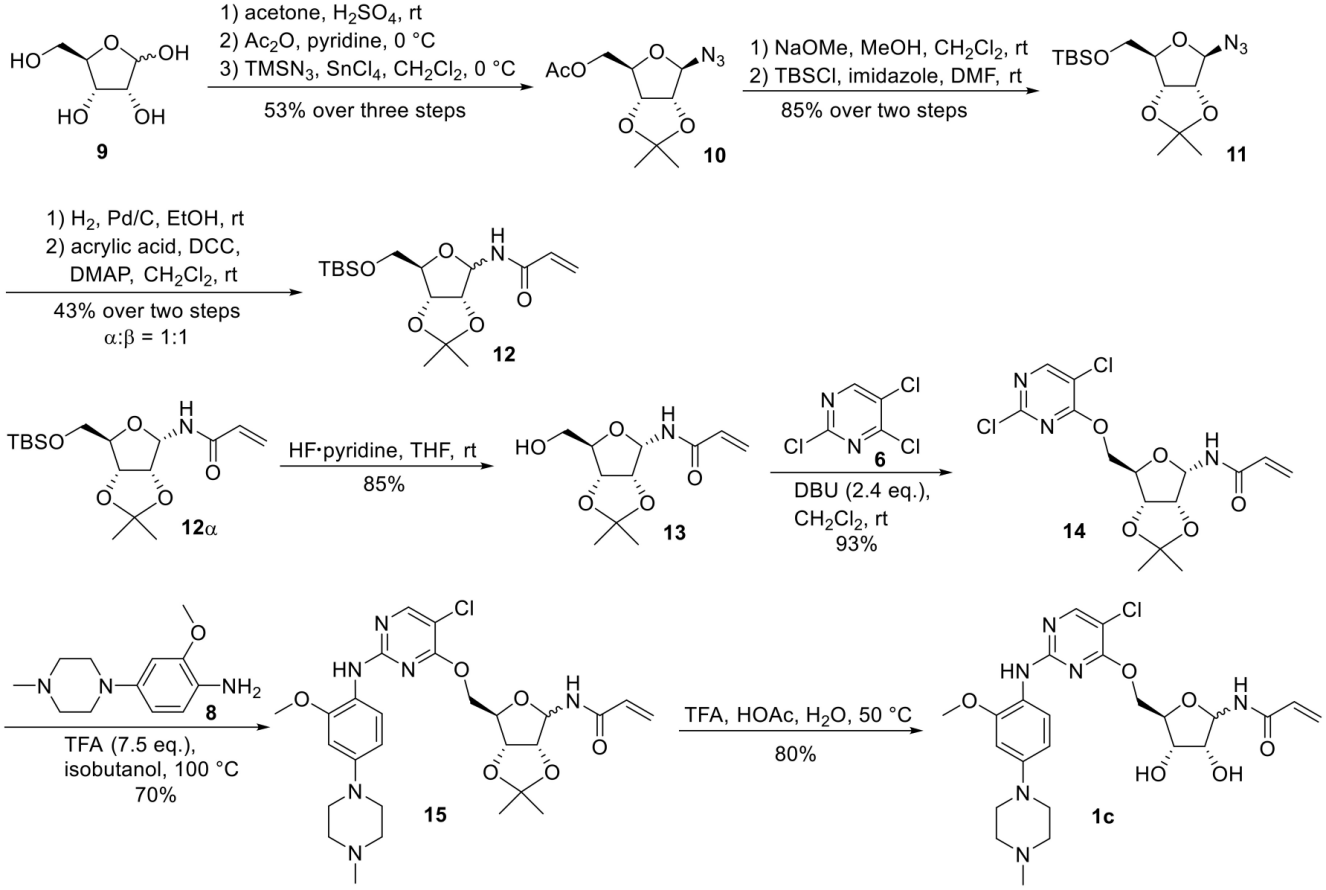
Synthesis of 1-*N*-acryloyl-5-*O*-anilinopyrimidine ribose derivative **1c**.

For the synthesis of 1-*N*-acryloyl-5-*O*-anilinopyrimidine ribose derivative **1c**, β-ribosyl azide **10** was conveniently prepared from d-ribose **9** in three steps and 53% yield according to the procedures described in the literature (Scheme 2; Bonache et al., 2009). The acetyl group in **10** was then replaced with TBS group to give compound **11** in 85% yield. Hydrogenolysis of the azide group in **11** over Pd/C followed by condensation with acrylic acid in the presence of DCC and DMAP afforded a mixture of ribose derivative **12** in 43% yield (α/β = 1:1; α-anomer: δ_H_ = 6.00 ppm, δ_C_ = 81.4 ppm; β-anomer: δ_H_ = 5.95 ppm, δ_C_ = 87.2 ppm), which were easily separated by silica gel column chromatography (Numao et al., 1981; Bonache et al., 2009). After removal of the TBS group in **12**α, the resulting alcohol **13** reacted with **6** in the presence of DBU to produce ribose derivative **14** in an excellent 93% yield. TFA-promoted reaction of **14** with aniline **8** led to **15** in 70% yield as a mixture of α/β anomers probably arising from the anomerization of the 1-*N*-acryloyl ribose derivative under strong acidic conditions (Boschelli et al., 1989). Finally, acidic cleavage of the isopropylidene group of **15** in the mixture of TFA/HOAc/water provided **1c** in 80% yield.

**Scheme 3:**
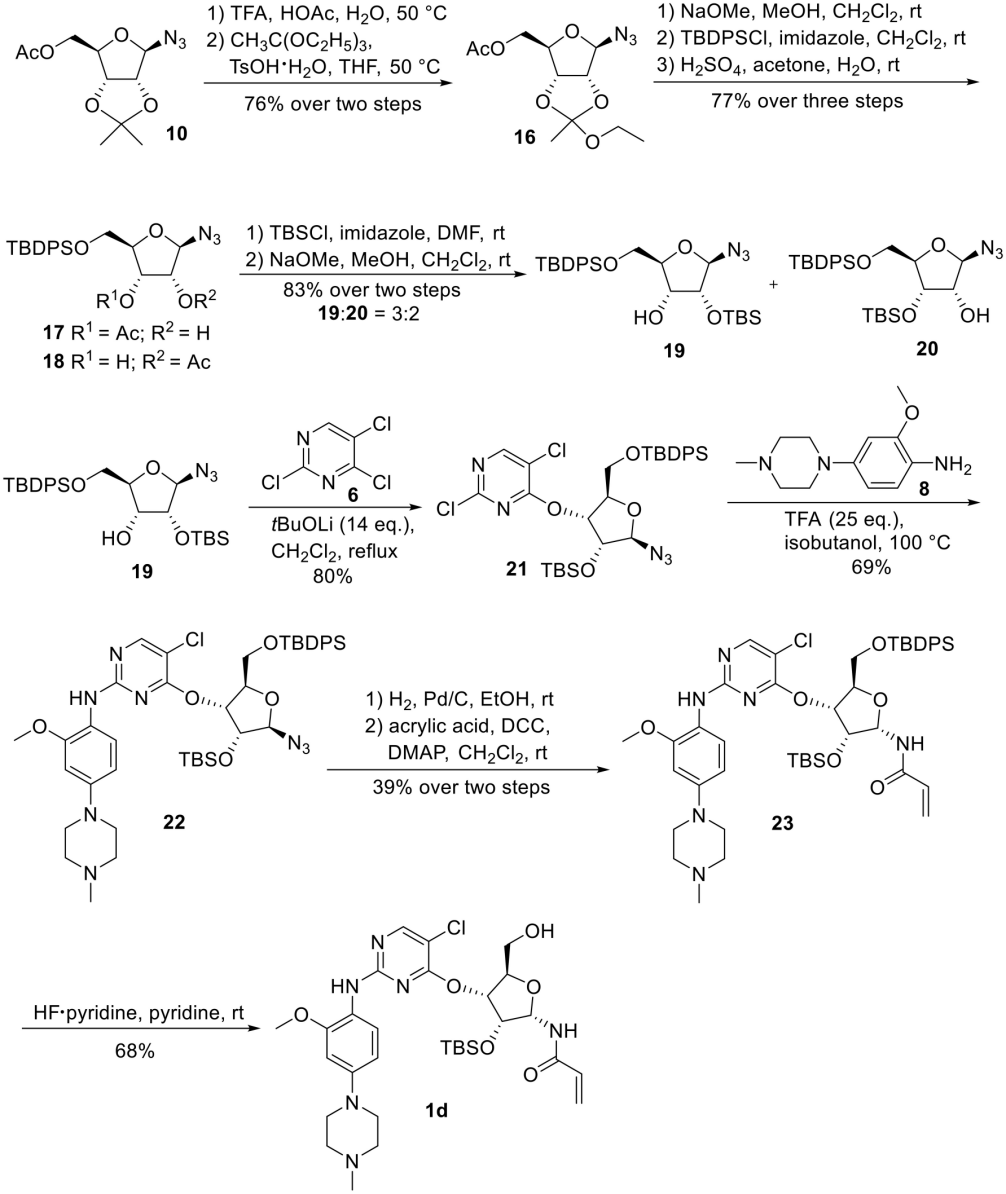
Synthesis of 1-*N*-acryloyl-3-*O*-anilinopyrimidine ribose derivative **1d**.

Synthetic work toward 1-*N*-acryloyl-3-*O*-anilinopyrimidine ribose derivative **1d** started from replacement of the 2,3-isopropylidene group of azide **10** with 2,3-orthoester group by treatment with TFA and subsequent protection with triethyl orthoacetate under the catalysis of TsOH·H_2_O, affording azide **16** in 76% yield over two steps (Scheme 3). Substitution of the acetyl group in **16** with TBDPS group and subsequent acidic cleavage of the orthoester group led to an inseparable mixture of 2-acetyl and 3-acetyl ribose derivatives **17** and **18** (77% yield over three steps). Treatment of the mixture of **17** and **18** with TBSCl followed by removal of the acetyl groups gave alcohols **19** and **20** in 83% yield, allowing for the separation of 3-hydroxyl ribose derivative **19** from 2-hydroxyl ribose derivative **20** (**19**:**20** = 3:2). Nucleophilic attack of **19** on **6** required stronger basic conditions to promote the reaction due to the steric hindrance of the silyl groups on **19**. As such, excess *t*BuOLi in dichloromethane under reflux was employed for this conversion, providing ribose derivative **21** in 80% yield. TFA-promoted coupling of **21** with aniline **8** generated ribose derivative **22** (69%), which was then subjected to hydrogenolysis over Pd/C and subsequent condensation with acrylic acid to afford ribose derivative **23** as single anomer in moderate yield (39% over two steps). Exposure of **23** to HF·pyridine in pyridine resulted in cleavage of the TBDPS group without affecting the TBS group, providing **1d** in 68% yield.

To determine whether the Michael acceptor played a significant role in the inhibitory activity of ribose-modified pyrimidine derivatives against EGFR tyrosine kinase, 1-azide-5-*O*-anilinopyrimidine ribose derivative **24**, 1-azide-3-*O*-anilinopyrimidine ribose derivative **25**, and 5-*O*-anilinopyrimidine ribose derivative **26** were readily synthesized following the similar procedures described for **1a**-**1d** (Table 1; see Supplementary Material for details).

**Table 1:** *In vitro* inhibitory activities of ribose-modified pyrimidine derivatives **1a**–**1d** and **24**–**26** against EGFR tyrosine kinase.

** 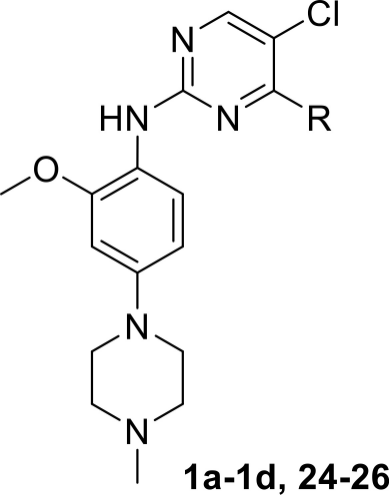 **			
**Compound**	**R**	**EGFR tyrosine kinase IC_50_ (μM)^a^**	
		**EGFR**	**EGFR^T790M/L858R^**
**1a**	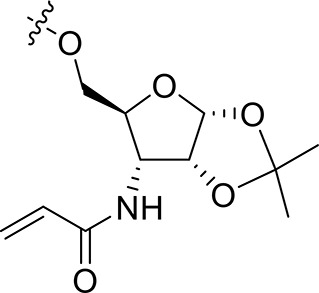	>10	0.6204 ± 0.1729
**1b**	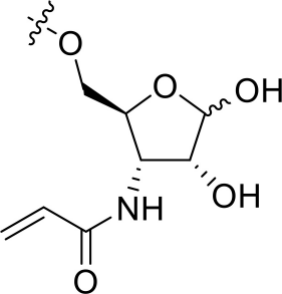	>10	2.6435 ± 1.8606
**1c**	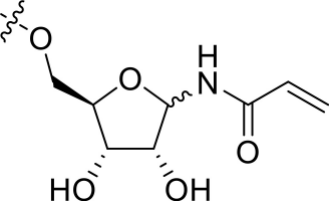	>10	>10
**1d**	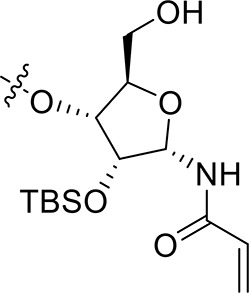	>10	>10
**24**	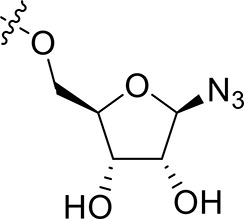	>10	>10
**25**	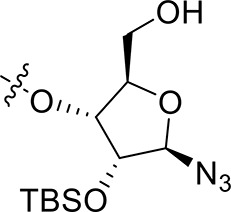	>10	>10
**26**	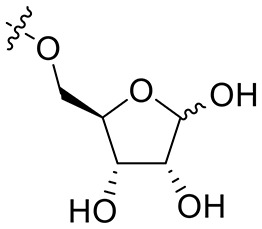	>10	>10
Osimertinib^b^		0.1586 ± 0.0428	0.0015 ± 0.0001
Afatinib^b^		0.00697 ± 0.00052	0.00374 ± 0.00035

a *Kinase activity assays were examined by using the ELISA-based EGFR-TK assay. Data are averages of at least two independent determinations and reported as the mean ± SD (standard deviation)*.

b *Reported data*.

As shown in Table 1, compounds **1a** and **1b** containing 3-*N*-acryloyl-5-*O*-anilinopyrimidine ribosyl moiety potently inhibited EGFR L858R/T790M mutant with IC_50_ values of 0.62 and 2.64 μM, revealing specific inhibitory activity for EGFR L858R/T790M over WT EGFR, although they are not comparable to the positive controls osimertinib (IC_50_ = 1.5 nM for EGFR L858R/T790M) and afatinib (IC_50_ = 3.7 nM for EGFR L858R/T790M). In contrast, other compounds (**1c**, **1d**, and **24**–**26**) bearing 5-*O*-anilinopyrimidine ribosyl moiety, 1-*N*-acryloyl-5-*O*-anilinopyrimidine ribosyl moiety, 1-*N*-acryloyl-3-*O*-anilinopyrimidine ribosyl moiety, or their 1-azide counterparts, showed no inhibitory activities against EGFR tyrosine kinases.

**Figure 3:**
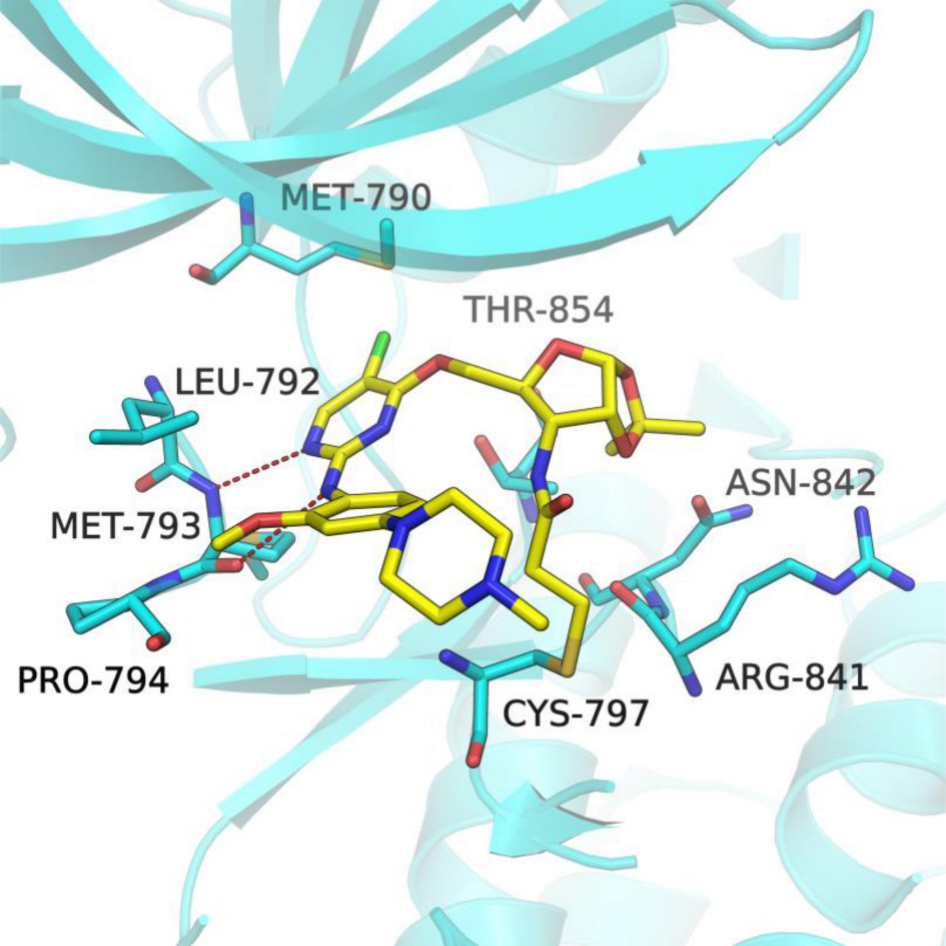
Docking pose of compound **1a** in complex with the EGFR^T790M/L858R^ (PDB ID 3IKA). The EGFR kinase is shown as cartoon (cyan) with the bound inhibitor in a stick representation (yellow). Key residues are represented as cyan sticks. The expected hydrogen bonds are indicated by red dashes.

In order to better understand the mechanism of this type of compounds binding to EGFR T790M, molecular docking was adopted to predict the binding mode of the representative compound **1a**. The docking procedure was validated in advance by re-docking the co-crystallized ligand WZ4002 (Zhou et al., 2009) into the ATP binding site of EGFR L858R/T790M structure (PDB ID: 3IKA). The root mean square deviation (RMSD) between the crystallographic and docked conformation of WZ4002 is 0.57 Å (Figure S1), demonstrating that the present docking procedure was feasible in generating the binding conformation accurately. As expected based upon co-crystal structure of the anilinopyrimidine-derived inhibitor WZ4002, the anilinopyrimidine core of compound **1a** forms a bidentate hydrogen bonding interaction with the “hinge” residue Met793 (Figure 3). The chlorine substituent on the pyrimidine ring could form hydrophobic contact with the mutant gatekeeper residue, Met790. The aniline ring is oriented to form hydrophobic interactions with Leu792 and Pro794 in the hinge region. Moreover, the acrylamide group attached to the sugar ring of compound **1a** could form a covalent bond with Cys797 to achieve irreversible binding. The sugar ring acts like a linker to tune the orientation of the electrophilic acrylamide moiety that can covalently alkylate the conserved cysteine residue Cys797. For the 1,2-*O*-isopropylidene moiety in compound **1a**, it could form favorable vdW interactions with residues ARG841, ASN842, and Thr854. Therefore, compound **1b** without that protecting group on the sugar ring, displayed less potent bioactivity against EGFR T790M/L858R compared with compound **1a**. Lacking of the Michael receptor, compounds **24**–**26** are unable to form covalent bond with Cys797 and thus displayed sharply decreased inhibitory activity against EGFR T790M/L858R. Compound **1c** displayed no inhibitory activity of EGFR probably because of the long distance between the Michael receptor and Cys797. Although compound **1d** also has an acrylamide group attached to the sugar ring, it showed no inhibitory activity probably due to the conformational alteration of compound **1d** caused by the TBS protecting group. Briefly, it could be concluded that the distance between the Michael receptor and the pyrimidine scaffold has a significant effect on the inhibitory potency of this type of compounds. Employing the ribosyl moiety as a chiral building block for modulating the distance between the Michael receptor and the pyrimidine scaffold could pave a new avenue for future design of EGFR inhibitors against EGFR mutants.

## Conclusion

In summary, we have described a DBU- or *t*BuOLi-promoted coupling of ribosyl alcohols with 2,4,5-trichloropyrimidine as key step for the synthesis of a series of ribose-modified anilinopyrimidine derivatives as EGFR TKIs. Preliminary biological evaluation indicated that compound **1a** displayed potent inhibitory activity against EGFR L858R/T790M with an IC_50_ value of 0.62 μM, and good selectivity for EGFR L858R/T790M over WT EGFR. Molecular docking studies revealed that the inhibitory activities of this type of compounds are largely influenced by the distance between the Michael receptor and the pyrimidine scaffold. As a novel type of EGFR inhibitor, the ribose-modified anilinopyrimidine derivative **1a** might be used as a promising lead compound for further development of selective EGFR inhibitors to overcome EGFR L858R/T790M resistance mutation.

## Author contributions

YY and YX designed and guided this study. XH conducted the chemical synthesis. YT, LT, LZ, and HX performed the kinase activity assays. DW, XW, and SL performed the molecular docking studies. XH, YY, and YX analyzed the data and wrote the manuscript with input from all authors.

### Conflict of interest statement

The authors declare that the research was conducted in the absence of any commercial or financial relationships that could be construed as a potential conflict of interest.
