# Supplementary material for: Design, Synthesis, and Evaluation of Ribose-Modified Anilinopyrimidine Derivatives as EGFR Tyrosine Kinase Inhibitors
Source: Front Chem. 2017 Nov 15;5:101. doi: 10.3389/fchem.2017.00101 (PMC5715404; doi:10.3389/fchem.2017.00101)
Supplement: Supplementary file 1 [file DataSheet1.PDF]

Supporting Information

Design, Synthesis and Evaluation of Ribose-modified Anilinopyrimidine Derivatives as EGFR Tyrosine Kinase Inhibitors

Xiuqin Hu[1], Disha Wang[1], Yi Tong[1], Linjiang Tong[2], Xia Wang[1], Lili Zhu[1], Hua Xie[2], Shiliang Li[1], You Yang[1]* and Yufang Xu[1]*

[1] Shanghai Key Laboratory of New Drug Design, School of Pharmacy, East China University of Science and Technology, 130 Meilong Road, Shanghai 200237, China.

[2] Division of Anti-tumor Pharmacology, State Key Laboratory of Drug Research, Shanghai Institute of Materia Medica, Chinese Academy of Sciences, Shanghai 201203, China.

*Correspondence: yangyou@ecust.edu.cn; yfxu@ecust.edu.cn

Contents

	page
1. Experimental details and characterization data of new compounds	S2
2. Validation of the docking procedure	S12
3. References	S13
4. NMR spectra of new compounds	S14

1. Experimental details and characterization data of new compounds

1.1. Synthesis of 1,2-*O*-isopropylidene-3-*N*-acryloyl-3-deoxy-5-*O*-*tert*-butyldiphenylsilyl-α-D-ribofuranoside 4

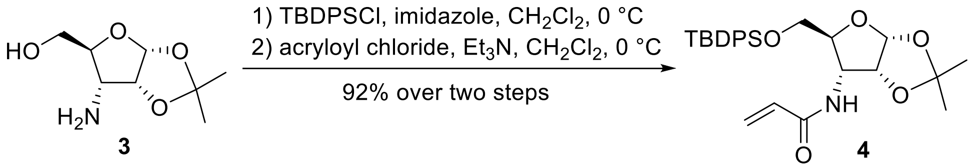

To a solution of compound **3**[1] (3.46 g, 18.30 mmol), imidazole (2.50 g, 36.60 mmol) in anhydrous CH_2Cl_2 (100 mL) at 0 ℃, was added TBDPSCl (5.24 mL, 20.13 mmol) under argon. After stirring at 0 ℃ for 2 h, the mixture was diluted with dichloromethane and washed with water and saturated aqueous $NaHCO_3$. The organic layer was dried over Na_2SO_4, filtered, and concentrated *in vacuo* to give the corresponding silyl ether for the next step without further purification.

To a solution of the resulting silyl ether in CH_2Cl_2 (60 mL) and Et_3N (60 mL) at 0 ℃, was added acryloyl chloride (2.70 mL, 27.45 mmol) dropwise. The reaction solution was stirred for 5 h at room temperature. The mixture was poured into saturated aqueous $NaHCO_3$ and extracted with CH_2Cl_2. The organic layer was washed with brine, dried over Na_2SO_4, and concentrated *in vacuo*. The residue was purified by silica gel chromatography (petroleum ether/EtOAc: 5/1) to afford **4** (8.09 g, 92 % over two steps) as a pale yellow syrup: ^{1}H NMR (400 MHz, $CDCl_3$) δ 7.71–7.68 (m, 4 H), 7.43–7.33 (m, 6 H), 6.30 (dd, *J* = 1.2, 17.2 Hz, 1 H), 6.08 (dd, *J* = 10.4, 17.2 Hz, 1 H), 5.86 (d, *J* = 3.6 Hz, 1 H, H-1), 5.78 (d, *J* = 8.8 Hz, 1 H, N*H*), 5.67 (dd, *J* = 1.2, 10.4 Hz, 1 H), 4.64 (t, *J* = 4.0 Hz, 1 H), 4.56 (m, 1 H), 3.91–3.86 (m, 2 H), 3.78 (dd, *J* = 4.8, 12.0 Hz, 1 H), 1.54 (s, 3 H), 1.35 (s, 3 H), 1.05 (s, 9 H); ^{13}C NMR (100 MHz, $CDCl_3$) δ 165.2, 135.9, 135.8, 133.5, 133.3, 130.6, 129.8, 129.7, 127.8, 127.3, 112.6, 104.7, 81.0, 79.4, 63.2, 51.9, 26.9, 26.6, 19.4; HRMS (ESI) *m/z* calcd for $C_{27}H_{35}O_5NSiNa$ $[M + Na]^+$ 504.2182, found 504.2191.

1.2. Synthesis of 1,2-*O*-isopropylidene-3-*N*-acryloyl-3-deoxy-α-D-ribofuranoside 5

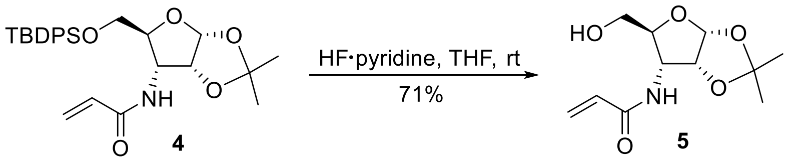

To a solution of compound **4** in THF (15 mL) at room temperature, was added HF·pyridine (9.30 mL). After stirring at room temperature for overnight, the mixture was poured into saturated aqueous $NaHCO_3$ and extracted with EtOAc. The combined organic layers were washed with brine, dried over Na_2SO_4, and concentrated *in vacuo*. The residue was purified by silica gel column chromatography (petroleum ether/EtOAc: 5/1) to afford **5** (0.81 g, 71%) as a pale yellow syrup: ^{1}H NMR (400 MHz, $CDCl_3$) δ 6.32 (dd, *J* = 1.2, 16.8 Hz, 1 H), 6.25 (d, *J* = 8.0 Hz, 1 H, N*H*), 6.14 (dd, *J* = 10.4, 17.2 Hz, 1 H), 5.86 (d, *J* = 4.0 Hz, 1 H, H-1), 5.71 (dd, *J* = 1.2, 10.4 Hz, 1 H), 4.63 (t, *J* = 4.4 Hz, 1 H), 4.28 (m, 1 H), 3.80 (m, 2 H), 3.68 (m, 1 H), 3.52 (br s, 1 H), 1.53 (s, 3 H), 1.34 (s, 3 H); ^{13}C NMR (100 MHz, $CDCl_3$) δ 166.5, 129.8, 128.3, 112.8, 104.3, 80.5, 79.1, 60.7, 51.9, 26.6, 26.5; HRMS (ESI) *m/z* calcd for $C_{11}H_{17}O_5NNa$ $[M + Na]^+$ 266.1004, found 266.0987.

1.3. Synthesis of 2,3-*O*-isopropylidene-5-*O*-*tert*-butyldimethylsilyl-β-D-ribofuranosyl azide 11

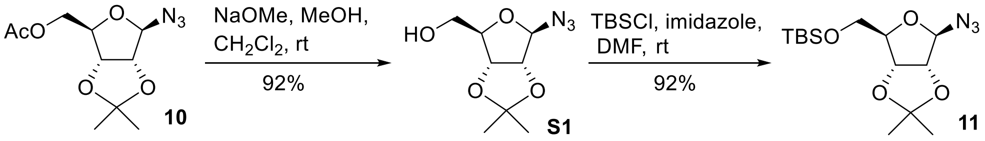

To a solution of compound **10**[2] (1.07 g, 4.16 mmol) in anhydrous CH_2Cl_2 (10 mL) and MeOH (10 mL) at room temperature, was added NaOMe (113 mg, 2.08 mmol). After stirring at room temperature for 1 h, the reaction mixture was diluted with methanol and neutralized with Amberlite IR120 H^+ resin. After filtration, the filtrate was concentrated *in vacuo* to give a residue, which was purified by silica gel column chromatography (petroleum ether/EtOAc: 4/1) to afford **S1** (0.82 g, 92%) as a colorless syrup: ^{1}H NMR (400 MHz, $CDCl_3$) δ 5.52 (s, 1 H, H-1), 4.74 (d, *J* = 6.0 Hz, 1 H), 4.50 (d, *J* = 6.0 Hz, 1 H), 4.38 (t, *J* = 4.4 Hz, 1 H), 3.75 (dd, *J* = 4.0, 12.0 Hz, 1 H), 3.66 (dd, *J* = 5.2, 12.0 Hz, 1 H), 1.48 (s, 3 H), 1.30 (s, 3 H); ^{13}C NMR (100 MHz,

$CDCl_3$) δ 113.2, 98.1, 88.7, 86.0, 81.8, 63.7, 26.6, 25.1; ESI-MS (ESI) *m/z* calcd for $C_8H_{13}O_4N_3Na$ [M + Na]$^+$ 238.1, found 238.1.

To a solution of **S1** (790 mg, 3.67 mmol), imidazole (1.87 g, 27.53 mmol) in anhydrous DMF (20 mL) at room temperature, was added TBSCl (2.20 g, 14.69 mmol) under argon. After stirring at room temperature for 2 h, the mixture was concentrated in vacuo to give a residue, which was purified by silica gel column chromatography (petroleum ether/EtOAc: 10/1) to afford **11** (1.11 g, 92%) as a colorless syrup: ^{1}H NMR (400 MHz, $CDCl_3$) δ 5.48 (s, 1 H, H-1), 4.72 (dd, *J* = 0.8, 6.0 Hz, 1 H), 4.45 (d, *J* = 6.0 Hz, 1 H), 4.30 (m, 1 H), 3.72 (dd, *J* = 5.2, 10.8 Hz, 1 H), 3.66 (dd, *J* = 7.4, 10.4 Hz, 1 H), 1.49 (s, 3 H), 1.32 (s, 3 H), 0.91 (s, 9 H), 0.08 (s, 6 H); ^{13}C NMR (100 MHz, $CDCl_3$) δ 113.0, 97.1, 88.1, 85.7, 82.3, 63.4, 26.8, 26.0, 25.3, 18.5, –5.3; ESI-MS (ESI) *m/z* calcd for $C_{14}H_{27}O_4N_3SiNa$ [M + Na]$^+$ 352.2, found 352.2.

1.4. Synthesis of 2,3-*O*-isopropylidene-5-*O*-*tert*-butyldimethylsilyl-D-ribofuranosyl acrylamide 12

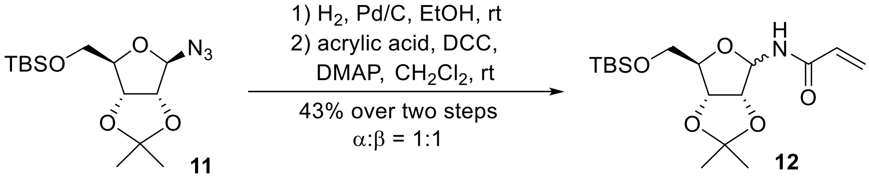

A mixture of compound **11** (1.11 g, 3.37 mmol) and Pd/C (0.51 g, 10%) in EtOH (34 mL) was stirred under an atmosphere of H_2 at room temperature for 4 h. The mixture was filtered through celite, washed with EtOH and concentrated *in vacuo* to afford the corresponding amine for the next step without further purification. To a solution of the resulting amine in CH_2Cl_2 (50 mL) at room temperature, was added DCC (1.39 g, 6.74 mmol), DMAP (0.82 g, 6.74 mmol), and acrylic acid (1.16 mL, 16.85 mmol). After stirring at room temperature for 3 h, the mixture was concentrated *in vacuo* to give a residue, which was purified by silica gel column chromatography (petroleum ether/EtOAc: 8/1) to afford **12** (518 mg, 43% over two steps, α/β = 1:1) as a colorless syrup.

12α: ^{1}H NMR (400 MHz, $CDCl_3$) δ 6.50 (d, *J* = 9.2 Hz, 1 H, N*H*), 6.32 (dd, *J* = 1.2, 17.2 Hz, 1 H), 6.13 (dd, *J* = 10.4, 17.2 Hz, 1 H), 6.00 (dd, *J* = 4.0, 9.2 Hz, 1 H, H-1), 5.68 (dd, *J* = 1.2, 10.4 Hz, 1 H), 4.81 (d, *J* = 6.0 Hz, 1 H), 4.62 (dd, *J* = 4.4, 6.0 Hz, 1 H), 4.09 (t, *J* = 2.4 Hz, 1 H), 3.78 (dd, *J* = 3.2, 11.2 Hz, 1 H), 3.67 (dd, *J* = 2.8, 11.2 Hz, 1 H), 1.53 (s, 3 H), 1.36 (s, 3 H), 0.89 (s, 9 H), 0.10 (s, 3 H), 0.07 (s, 3 H); ^{13}C NMR (100 MHz, $CDCl_3$) δ 165.1, 130.8, 127.8, 112.6, 82.5, 82.4, 81.4 (C-1), 80.0, 65.6, 26.4, 26.0, 24.8, 18.2, –5.4, –5.6; HRMS (ESI) *m/z* calcd for $C_{17}H_{32}O_5NSi$ [M + H]$^+$ 358.2050, found 358.2047. **12β**: ^{1}H NMR (400 MHz, $CDCl_3$) δ 6.92 (d, *J* = 9.2 Hz, 1 H, N*H*), 6.30 (dd, *J* = 1.2, 16.8 Hz, 1 H), 6.00 (dd, *J* = 10.4, 16.8 Hz, 1 H), 5.95 (d, *J* = 9.2 Hz, 1 H, H-1), 5.65 (dd, *J* = 1.2, 10.4 Hz, 1 H), 4.71 (d, *J* = 6.0 Hz, 1 H), 4.52 (d, *J* = 5.6 Hz, 1 H), 4.30 (br s, 1 H), 3.82 (dd, *J* = 1.6, 11.2 Hz, 1 H), 3.75 (dd, *J* = 2.0, 11.6 Hz, 1 H), 1.51 (s, 3 H), 1.31 (s, 3 H), 0.93 (s, 9 H), 0.14 (s, 3 H), 0.13 (s, 3 H); ^{13}C NMR (100 MHz, $CDCl_3$) δ 164.6, 130.7, 127.7, 112.8, 87.4, 87.2 (C-1), 86.8, 82.2, 65.5, 26.9, 26.1, 25.3, 18.7, –5.1, –5.2; HRMS (ESI) *m/z* calcd for $C_{17}H_{31}O_5NSiNa$ [M + Na]$^+$ 380.1869, found 380.1862.

1.5. Synthesis of 2,3-*O*-isopropylidene-α-D-ribofuranosyl acrylamide 13

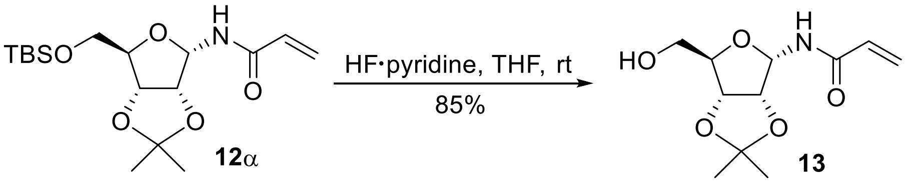

To a solution of **12α** (253 mg, 0.71 mmol) in THF (15 mL) at room temperature, was added HF·pyridine (1.28 mL). After stirring at room temperature for overnight, the mixture was poured into saturated aqueous $NaHCO_3$ and extracted with EtOAc. The combined organic layers were washed with brine, dried over Na_2SO_4, and concentrated *in vacuo*. The residue was purified by silica gel column chromatography (petroleum ether/EtOAc: 2/1) to afford **13** (147 mg, 85%) as a colorless syrup: ^{1}H NMR (400 MHz, $CDCl_3$) δ 6.71 (d, *J* = 8.8 Hz, 1 H, N*H*), 6.32 (dd, *J* = 1.2, 17.2 Hz, 1 H), 6.15 (dd, *J* = 10.4, 17.2 Hz, 1 H), 5.93 (dd, *J* = 4.0, 8.8 Hz, 1 H, H-1), 5.72 (dd, *J* = 1.2, 10.4 Hz, 1 H), 4.80 (dd, *J* = 1.2, 6.4 Hz, 1 H), 4.71 (dd, *J* = 4.4, 6.0 Hz, 1 H),

4.12 (t, J = 3.2 Hz, 1 H), 3.78 (m, 1 H), 3.71 (t, J = 4.8 Hz, 1 H), 3.64 (m, 1 H), 1.53 (s, 3 H), 1.36 (s, 3 H); ^{13}C NMR (100 MHz, $CDCl_3$) δ 165.8, 130.6, 128.3, 113.1, 83.0, 82.1, 80.9, 79.7, 63.6, 26.4, 24.8; HRMS (ESI) *m/z* calcd for $C_{11}H_{17}O_5NNa$ $[M + Na]^+$ 266.1004, found 266.0968.

1.6. Synthesis of 5-*O*-acetyl-2,3-*O*-ethylorthoacetyl-β-D-ribofuranosyl azide 16

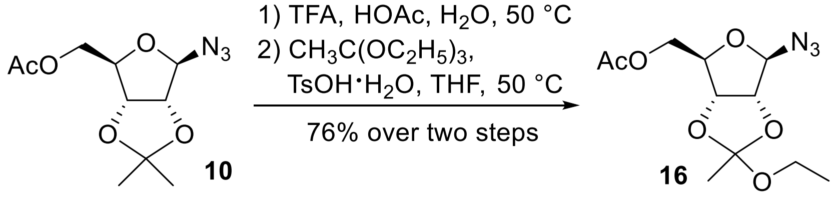

A solution of compound **10** (6.05 g, 23.53 mmol) in TFA/acetic acid/water (1/20/4, v/v/v, 130 mL) was stirred at 50 ℃ for 5 h. The solution was concentrated *in vacuo* to give the corresponding diol for the next step without further purification. To a solution of the above diol in THF (130 mL), was added triethyl orthoacetate (6.47 mL, 35.28 mmol) and $TsOH·H_2O$ (0.45 g, 2.35 mmol). After stirring at 50 ℃ for 5h, the mixture was poured into saturated aqueous $NaHCO_3$ and extracted with EtOAc. The combined organic layers were washed with brine, dried over Na_2SO_4, and concentrated *in vacuo*. The residue was purified by silica gel column chromatography (petroleum ether/EtOAc: 8/1) to afford **16** (5.13 g, 76% over two steps) as a white syrup: HRMS (ESI) *m/z* calcd for $C_{11}H_{17}O_6N_3Na$ $[M + Na]^+$ 310.1015, found 310.1015.

1.7. Synthesis of 5-*O*-*tert*-butyldiphenylsilyl-3-*O*-acetyl-β-D-ribofuranosyl azide 17 and 5-*O*-*tert*-butyldimethylsilyl-2-*O*-acetyl-β-D-ribofuranosyl azide 18

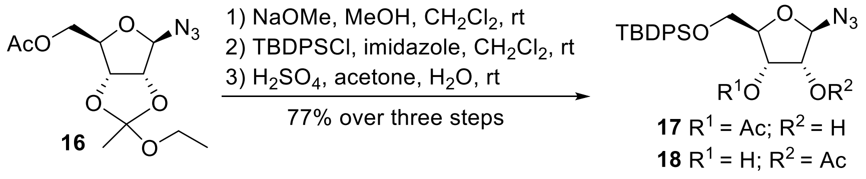

To a solution of compound **16** (5.04 g, 17.55 mmol) in anhydrous CH_2Cl_2 (40 mL) and MeOH (30 mL) at room temperature, was added NaOMe (0.29 g, 5.27 mmol). After stirring at room temperature for 1 h, the reaction mixture was diluted

with CH_2Cl_2, washed with brine, and dried over Na_2SO_4. Filtration and concentrated *in vacuo* gave the corresponding alcohol for the next step without further purification. To a solution of the resulting alcohol, imidazole (2.39 g, 35.11 mmol) in anhydrous CH_2Cl_2 (80 mL) at room temperature, was added TBDPSCl (5 mL, 19.31 mmol) under argon. After stirring at room temperature for 2.5 h, the reaction mixture was diluted with CH_2Cl_2, washed with brine, and dried over Na_2SO_4. Filtration and concentrated *in vacuo* gave the corresponding silyl ether for the next step without further purification. A solution of the resulting silyl ether in H_2SO_4/acetone/water (1/38/8, v/v/v, 61.3 mL) was stirred at room temperature for 50 min. The mixture was poured into saturated aqueous $NaHCO_3$ and extracted with EtOAc. The combined organic layers were washed with brine, dried over Na_2SO_4, and concentrated *in vacuo*. The residue was purified by silica gel column chromatography (petroleum ether/EtOAc: 8/1) to afford a mixture of **17** and **18** (6.15 g, 77% over three steps) as a white powder: HRMS (ESI) *m/z* calcd for $C_{23}H_{29}O_5N_3SiNa$ $[M + Na]^+$ 478.1774, found 478.1772.

1.8. Synthesis of 5-*O*-*tert*-butyldiphenylsilyl-2-*O*-*tert*-butyldimethylsilyl-β-D-ribofuranosyl azide 19 and 5-*O*-*tert*-butyldiphenylsilyl-3-*O*-*tert*-butyldimethylsilyl-β-D-ribofuranosyl azide 20

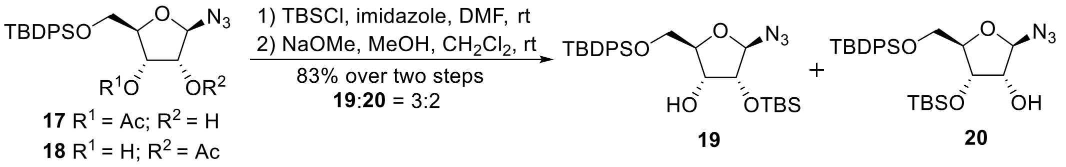

To a solution of **17**/**18** (1.88 g, 4.13 mmol), imidazole (2.11 g, 30.98 mmol) in anhydrous DMF (40 mL) at room temperature, was added TBSCl (2.49 g, 16.52 mmol) under argon. After stirring at room temperature for overnight, the reaction mixture was diluted with CH_2Cl_2, washed with brine, and dried over Na_2SO_4. Filtration and concentrated *in vacuo* gave the corresponding silyl ether for the next step without further purification. To a solution of the resulting silyl ether in anhydrous CH_2Cl_2 (15 mL) and MeOH (25 mL) at room temperature, was added NaOMe (130

mg, 2.40 mmol). After stirring at room temperature for overnight, the reaction mixture was neutralized with Amberlite IR120 H^+ resin. After filtration, the filtrate was concentrated *in vacuo* to give a residue, which was purified by silica gel column chromatography (petroleum ether/EtOAc: 50/1) to afford **19** and **20** (1.80 g, 83% over two steps, **19**:**20** = 3:2) as a yellow syrup. **19**: ^{1}H NMR (400 MHz, $CDCl_3$) δ 7.73–7.68 (m, 4 H), 7.45–7.37 (m, 6 H), 5.18 (d, *J* = 3.2 Hz, 1 H, H-1), 4.22 (dd, *J* = 4.8, 10.8 Hz, 1 H), 4.05 (m, 2 H), 3.85 (dd, *J* = 2.8, 11.6 Hz, 1 H), 3.73 (dd, *J* = 3.6, 11.2 Hz, 1 H), 2.51 (d, *J* = 6.0 Hz, 1 H), 1.08 (s, 9 H), 0.93 (s, 9 H), 0.16 (s, 3 H), 0.15 (s, 3 H); ^{13}C NMR (100 MHz, $CDCl_3$) δ 135.9, 135.8, 133.3, 133.1, 130.0, 129.9, 128.0, 127.9, 95.2, 85.7, 76.9, 71.5, 64.0, 27.0, 25.9, 19.4, 18.2, –4.4, –4.8; HRMS (ESI) *m/z* calcd for $C_{27}H_{41}O_4N_3Si_2Na$ $[M + Na]^+$ 550.2533, found 550.2534. **20**: ^{1}H NMR (400 MHz, $CDCl_3$) δ 7.73–7.69 (m, 4 H), 7.46–7.37 (m, 6 H), 5.34 (d, *J* = 2.0 Hz, 1 H, H-1), 4.45 (t, *J* = 5.6 Hz, 1 H), 4.00 (m, 1 H), 3.88–3.82 (m, 2 H), 3.65 (dd, *J* = 4.0, 11.6 Hz, 1 H), 2.76 (d, *J* = 4.0 Hz, 1 H), 1.08 (s, 9 H), 0.89 (s, 9 H), 0.10 (s, 3 H), 0.05 (s, 3 H); ^{13}C NMR (100 MHz, $CDCl_3$) δ 135.9, 135.8, 133.3, 132.9, 130.0, 129.9, 128.0, 127.9, 95.3, 84.9, 75.7, 71.2, 62.9, 27.0, 25.8, 19.4, 18.1, –4.6, –4.7; HRMS (ESI) *m/z* calcd for $C_{27}H_{41}O_4N_3Si_2Na$ $[M + Na]^+$ 550.2533, found 550.2540.

1.9. Synthesis of 5-*O*-[5-chloro-2-*N*-(2-methoxy-4-(4-methylpiperazin-1-yl) phenyl)pyrimidin-4-yl]-β-D-ribofuranosyl azide 24

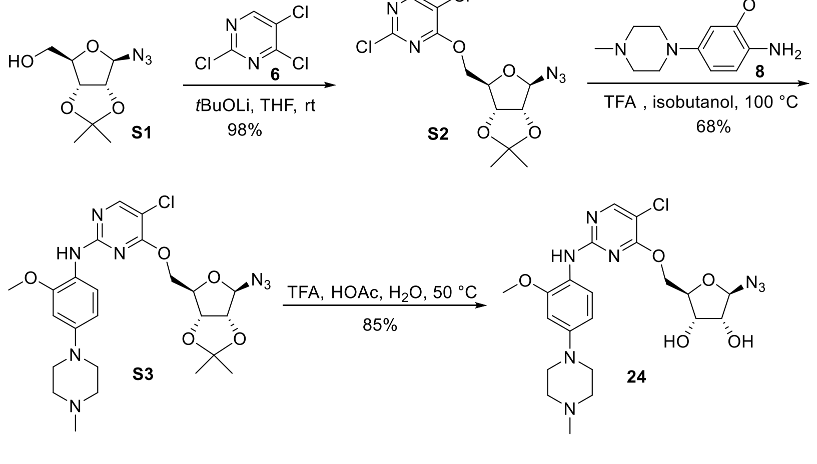

To a solution of compound **S1** (0.72 g, 3.35 mmol) in THF (20 mL) at room temperature, was added *t*BuOLi (1.12 g, 14.02 mmol) and 2,4,5-trichloropyrimidine **6**

(0.48 mL, 4.31 mmol). After stirring at room temperature for 2 h, the reaction mixture was diluted with saturated aqueous NH_4Cl, and extracted with CH_2Cl_2. The organic layer was washed with brine, dried over Na_2SO_4, and concentrated *in vacuo*. The residue was purified by silica gel chromatography (petroleum ether/EtOAc: 10/1) to give **S2** (1.19 g, 98%) as a pale yellow syrup: ^{1}H NMR (500 MHz, $CDCl_3$) δ 8.36 (s, 1 H), 5.57 (s, 1 H), 4.82 (dd, *J* = 1.0, 7.5 Hz, 1 H), 4.68 (m, 1 H), 4.62–4.56 (m, 3 H), 1.51 (s, 3 H), 1.34 (s, 3 H); ^{13}C NMR (125 MHz, $CDCl_3$) δ 165.0, 158.9, 157.5, 117.0, 113.7, 97.3, 85.6, 84.7, 81.9, 67.9, 26.7, 25.2; ESI-MS (ESI) *m/z* calcd for $C_{12}H_{14}O_4N_5Cl_2$ $[M + H]^+$ 362.0, found 362.3.

To a solution of compound **S2** (0.73 g, 2.02 mmol) and aniline derivative **8** (0.57 g, 2.57 mmol) in isobutanol (20 mL), was added TFA (0.93 mL, 15.22 mmol). The mixture was heated to 100 ℃ and stirred for 5 h. After cooling down to room temperature, the mixture was quenched with Et_3N (8 mL) and concentrated *in vacuo* to give a residue, which was purified by silica gel column chromatography (CH_2Cl_2/MeOH: 30/1) to give **S3** (0.75 g, 68%) as a pale yellow powder: ^{1}H NMR (400 MHz, $CDCl_3$) δ 8.13 (s, 1 H), 8.09 (d, *J* = 8.8 Hz, 1 H), 7.40 (br s, 1 H), 6.54 (m, 2 H), 5.57 (s, 1 H), 4.86 (d-like, *J* = 5.6 Hz, 1 H), 4.68 (t, *J* = 6.0 Hz, 1 H), 4.58–4.52 (m, 3 H), 3.88 (s, 3 H), 3.17 (br s, 4 H), 2.60 (br s, 4 H), 2.36 (s, 3 H), 1.50 (s, 3 H), 1.33 (s, 3 H); ^{13}C NMR (100 MHz, $CDCl_3$) δ 163.9, 157.8, 156.9, 149.3, 145.8, 123.4, 119.8, 113.5, 109.6, 106.5, 101.4, 97.2, 85.7, 84.9, 82.3, 66.3, 55.9, 54.0, 48.5, 44.0, 26.7, 25.2; HRMS (ESI) *m/z* calcd for $C_{24}H_{32}O_5N_8Cl$ $[M + H]^+$ 547.2184, found 547.2185.

A solution of compound **S3** (85 mg, 0.16 mmol) in TFA/acetic acid/water (1/20/4, v/v/v, 4 mL) was stirred at 50 ℃ for 5 h. Concentration *in vacuo* and elution through silica gel column chromatography (CH_2Cl_2/MeOH: 12/1) provided **24** (69 mg, 85%) as a pale yellow syrup: ^{1}H NMR (400 MHz, CD_3OD) δ 8.05 (s, 1 H), 7.95 (d, *J* = 8.8 Hz, 1 H), 6.65 (d, *J* = 2.4 Hz, 1 H), 6.54 (dd, *J* = 2.8, 8.8 Hz, 1 H), 5.23 (s, 1 H), 4.75 (dd, *J* = 2.8, 12.0 Hz, 1 H), 4.41 (dd, *J* = 4.0, 12.0 Hz, 1 H), 4.33 (dd, *J* = 4.4, 6.8 Hz, 1 H), 4.28 (m, 1 H), 3.88 (dd, *J* = 2.0, 4.4 Hz, 1 H), 3.86 (s, 3 H), 3.75 (br s, 2 H), 3.58 (br s, 2 H), 3.26 (br s, 2 H), 3.04 (br s, 2 H), 2.94 (s, 3 H); ^{13}C NMR (100 MHz,

CD_3OD) δ 165.7, 159.2, 157.0, 151.6, 147.6, 123.7, 122.2, 109.6, 107.1, 102.4, 96.3, 82.6, 76.3, 71.8, 67.4, 56.4, 54.8, 48.7, 43.5; HRMS (ESI) *m/z* calcd for $C_{21}H_{28}O_5N_8Cl$ [M + H]$^+$ 507.1871, found 507.1870.

1.10. Synthesis of 3-*O*-[5-chloro-2-*N*-(2-methoxy-4-(4-methylpiperazin-1-yl) phenyl)pyrimidin-4-yl]-2-*O*-*tert*-butyldimethylsilyl-β-D-ribofuranosyl azide 25

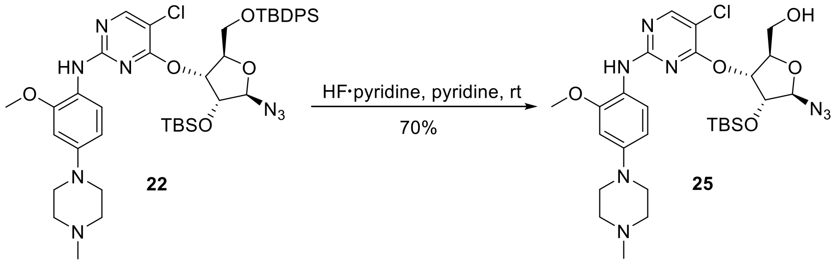

To a solution of compound **22** (100 mg, 0.12 mmol) in pyridine (3 mL) at room temperature, was added HF·pyridine (0.22 mL). After stirring at room temperature for overnight, the mixture was poured into saturated aqueous $NaHCO_3$ and extracted with CH_2Cl_2. The combined organic layers were washed with brine, dried over Na_2SO_4, and concentrated *in vacuo*. The residue was purified by silica gel column chromatography (CH_2Cl_2/MeOH: 20/1) to afford **25** (52 mg, 70%) as a pale yellow syrup: ^{1}H NMR (400 MHz, $CDCl_3$) δ 8.12 (s, 1 H), 8.02 (d, *J* = 8.4 Hz, 1 H), 7.35 (br s, 1 H), 6.55 (m, 2 H), 5.40 (dd, *J* = 4.4, 7.2 Hz, 1 H, H-3), 5.23 (d, *J* = 2.0 Hz, 1 H, H-1), 4.46 (m, 1 H), 4.36 (dd, *J* = 1.6, 4.4 Hz, 1 H), 3.94 (dd, *J* = 2.3, 12.4 Hz, 1 H), 3.87 (s, 3 H), 3.72 (dd, *J* = 3.6, 12.4 Hz, 1 H), 3.23 (t, *J* = 5.2 Hz, 4 H), 2.69 (t, *J* = 4.8 Hz, 4 H), 2.42 (s, 3 H), 0.79 (s, 9 H), –0.05 (s, 3 H), –0.22 (s, 3 H); ^{13}C NMR (100 MHz, $CDCl_3$) δ 163.7, 157.9, 157.0, 149.6, 147.6, 121.8, 120.5, 108.6, 106.2, 100.7, 96.0, 81.9, 75.0, 74.0, 62.0, 55.8, 55.1, 49.8, 45.9, 25.6, 18.0, –4.9, –5.3; HRMS (ESI) *m/z* calcd for $C_{27}H_{42}O_5N_8ClSi$ [M + H]$^+$ 621.2736, found 621.2741.

1.11. Synthesis of 5-*O*-[5-chloro-2-*N*-(2-methoxy-4-(4-methylpiperazin-1-yl) phenyl)pyrimidin-4-yl]-D-ribofuranose 26

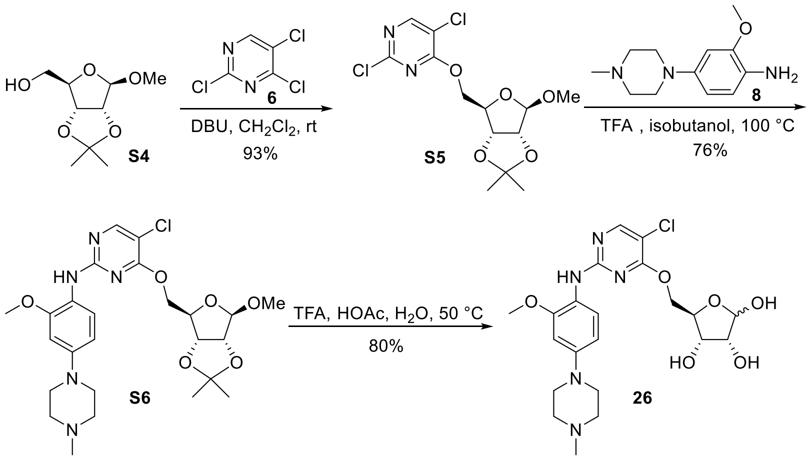

To a solution of compound **S4**[3] (0.72 g, 3.53 mmol) in CH_2Cl_2 (20 mL) at room temperature, was added DBU (1.07 g, 7.06 mmol) and 2,4,5-trichloropyrimidine **6** (0.61 mL, 5.30 mmol). After stirring at room temperature for 2 h, the reaction mixture was diluted with saturated aqueous NH_4Cl, and extracted with CH_2Cl_2. The organic layer was washed with brine, dried over Na_2SO_4, and concentrated *in vacuo*. The residue was purified by silica gel chromatography (petroleum ether/EtOAc: 20/1) to give **S5** (1.15 g, 93%) as a pale yellow syrup: ^1H NMR (400 MHz, $CDCl_3$) δ 8.34 (s, 1 H), 5.01 (s, 1 H), 4.78 (d-like, J = 5.6 Hz, 1 H), 4.65 (d-like, J = 6.0 Hz, 1 H), 4.60–4.47 (m, 3 H), 3.31 (s, 3 H), 1.49 (s, 3 H), 1.33 (s, 3 H); ^{13}C NMR (100 MHz, $CDCl_3$) δ 165.2, 157.5, 157.3, 117.0, 112.9, 109.8, 85.4, 83.8, 81.9, 68.6, 55.3, 26.6, 25.1; HRMS (ESI) *m/z* calcd for $C_{13}H_{16}O_5N_2Cl_2Na$ $[M + Na]^+$ 373.0334, found 373.0357.

To a solution of compound **S5** (1.0 g, 2.86 mmol) and aniline derivative **8** (1.26 g, 5.72 mmol) in isobutanol (20 mL), was added TFA (1.59 mL, 21.45 mmol). The mixture was heated to 100 ℃ and stirred for 5 h. After cooling down to room temperature, the mixture was quenched with Et_3N (10 mL) and concentrated *in vacuo* to give a residue, which was purified by silica gel column chromatography (CH_2Cl_2/MeOH: 30/1) to give **S6** (1.16 g, 76%) as a pale yellow powder: ^1H NMR (400 MHz, $CDCl_3$) δ 8.13 (d, J = 8.8 Hz, 1 H), 8.11 (s, 1 H), 7.38 (br s, 1 H), 6.53 (m,

2 H), 5.02 (s, 1 H), 4.81 (d-like, *J* = 6.0 Hz, 1 H), 4.65 (t, *J* = 6.0 Hz, 1 H), 4.60 (t, *J* = 6.8 Hz, 1 H), 4.51–4.40 (m, 2 H), 3.88 (s, 3 H), 3.35 (s, 3 H), 3.23 (t, *J* = 4.8 Hz, 4 H), 2.71 (t, *J* = 4.8 Hz, 4 H), 2.44 (s, 3 H), 1.49 (s, 3 H), 1.33 (s, 3 H); ^{13}C NMR (100 MHz, $CDCl_3$) δ 164.1, 157.9, 156.7, 149.2, 146.8, 122.7, 119.8, 112.7, 109.7, 108.9, 106.4, 101.0, 85.4, 84.0, 82.1, 67.0, 55.8, 55.1, 54.9, 49.6, 45.5, 26.6, 25.2; HRMS (ESI) *m/z* calcd for $C_{25}H_{35}O_6N_5Cl$ $[M + H]^+$ 536.2276, found 536.2266.

A solution of compound **S6** (200 mg, 0.37 mmol) in TFA/acetic acid/water (1/20/4, v/v/v, 10 mL) was stirred at 50 ℃ for 5 h. Concentration *in vacuo* and elution through silica gel column chromatography (CH_2Cl_2/MeOH: 12/1) provided **26** (142 mg, 80%) as a pale yellow syrup: ^{1}H NMR (400 MHz, CD_3OD) δ 7.99 (s, 1 H), 7.98 (s, 2 H), 7.92 (m, 3 H), 6.59 (d, *J* = 2.0 Hz, 2 H), 6.48 (d, *J* = 8.4 Hz, 2 H), 5.30 (d, *J* = 4.0 Hz, 1 H), 5.18 (d, *J* = 0.8 Hz, 2 H), 4.62 (dd, *J* = 2.8, 11.6 Hz, 2 H), 4.50 (dd, *J* = 4.4, 12.8 Hz, 1 H), 4.39–4.32 (m, 4 H), 4.26–4.18 (m, 4 H), 4.14–4.08 (m, 2 H), 3.92 (dd, *J* = 1.2, 4.4 Hz, 2 H), 3.83 (s, 9 H), 3.70–3.56 (m, 12 H), 3.24–3.04 (m, 12 H), 2.94 (s, 9 H); HRMS (ESI) *m/z* calcd for $C_{21}H_{29}O_6N_5Cl$ $[M + H]^+$ 482.1806, found 482.1801.

2. Validation of the docking procedure

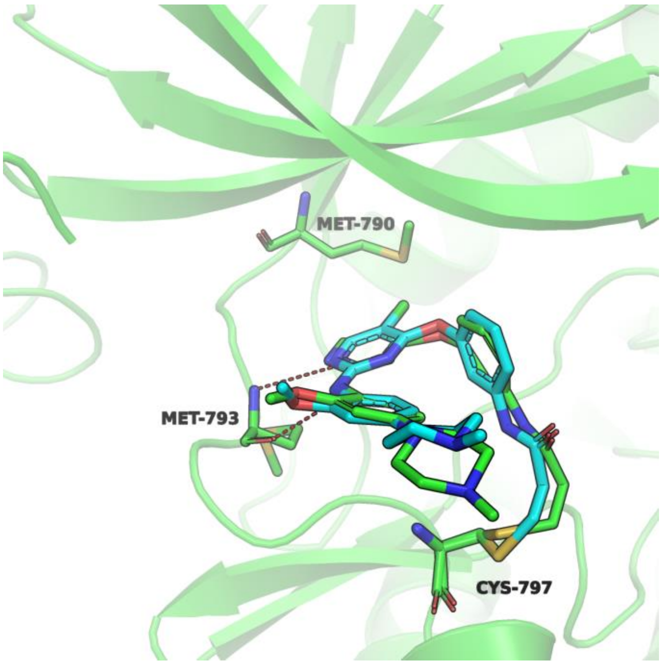

Figure S1. Validation of the docking procedure by comparing the crystal pose

(green) with the docked one (cyan). Key residues are represented as green sticks. Hydrogen bonds are indicated by red dashes.

3. References

(1) Shie, J.-J., Fang, J.-M., Wang, S.-Y., Tsai, K.-C., Cheng, Y.-S. E., Yang, A.-S., et al. (2007). Synthesis of tamiflu and its phosphonate congeners possessing potent anti-influenza activity. *J. Am. Chem. Soc.* 129, 11892–11893. doi: 10.1021/ja073992i

(2) Bonache, M. A., Nuti, F., Le Chevalier Isaad, A., Real-Fernandez, F., Chelli, M., Rovero, P., et al. (2009). Synthesis of new ribosylated Asn building blocks as useful tools for glycopeptide and glycoprotein synthesis. *Tetrahedron Lett.* 50, 4151–4153. doi:10.1016/j.tetlet.2009.04.124

(3) Leonard, N. J., and Carraway, K. L. (1966). 5-Amino-5-deoxyribose derivatives. Synthesis and use in the preparation of “Reversed” Nucleosides. *J. Heterocycl. Chem.* 3, 485–489. doi: 10.1002/jhet.5570030420

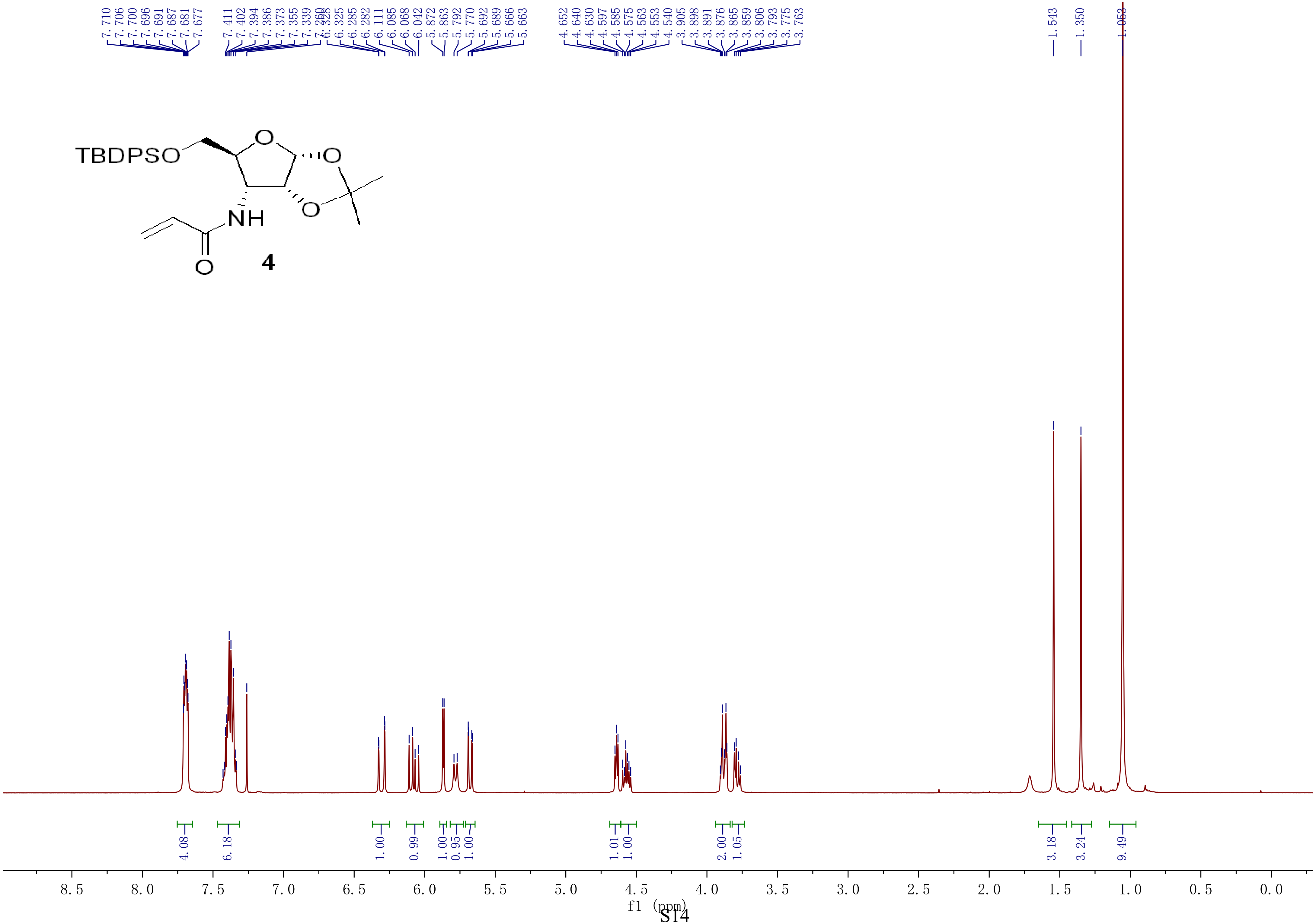
7.710
7.706
7.700
7.696
7.691
7.687
7.681
7.677
7.411
7.402
7.394
7.386
7.373
7.355
7.339
7.260
6.328
6.325
6.285
6.282
6.111
6.085
6.068
6.042
5.872
5.863
5.792
5.770
5.692
5.689
5.666
5.663
4.652
4.640
4.630
4.597
4.585
4.575
4.563
4.553
4.540
3.905
3.898
3.891
3.876
3.865
3.859
3.806
3.793
3.775
3.763
1.543
1.350
1.053
TBDPSO
NH
O
4
4.08
6.18
1.00
0.99
1.00
0.95
1.00
1.01
1.00
2.00
1.05
3.18
3.24
9.49
8.5
8.0
7.5
7.0
6.5
6.0
5.5
5.0
4.5
4.0
3.5
3.0
2.5
2.0
1.5
1.0
0.5
0.0
f1 (ppm)

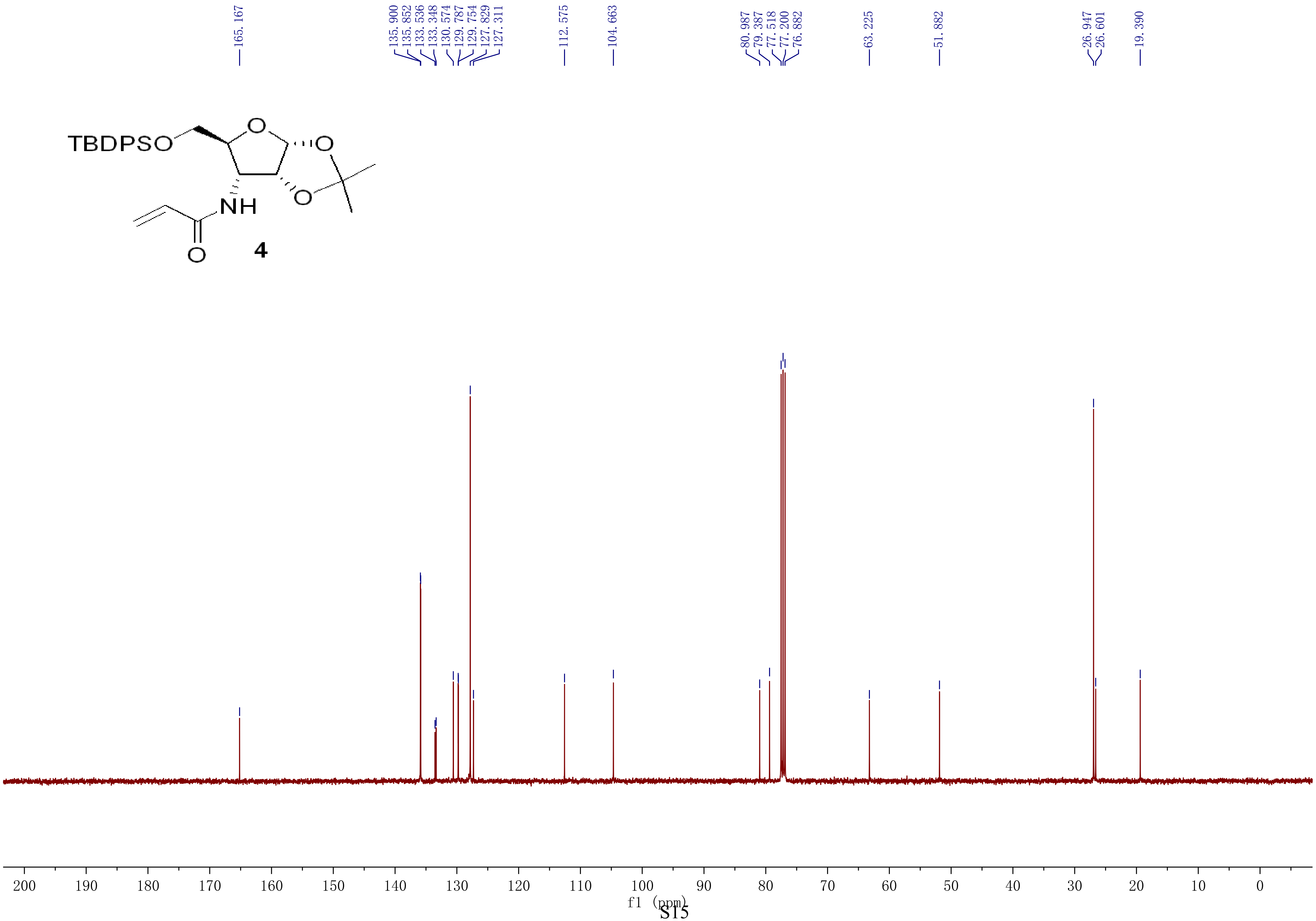
165.167
135.900
135.852
133.536
133.348
130.574
129.787
129.754
127.829
127.311
112.575
104.663
80.987
79.387
77.518
77.200
76.882
63.225
51.882
26.947
26.601
19.390
TBDPSO
NH
O
4
f1 (ppm)

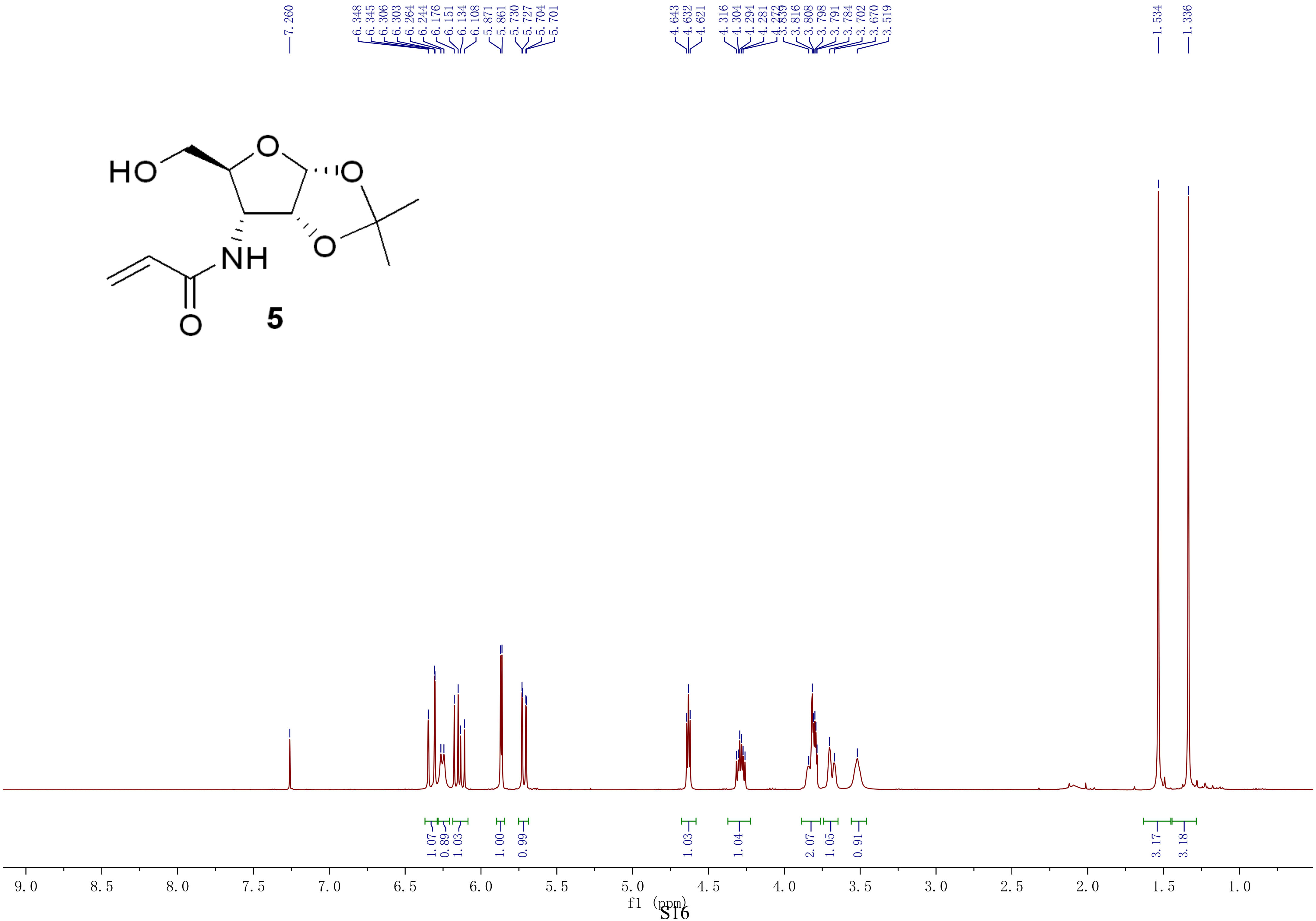
5
7.260
6.348
6.345
6.306
6.303
6.264
6.244
6.176
6.151
6.134
6.108
5.871
5.861
5.730
5.727
5.704
5.701
4.643
4.632
4.621
4.316
4.304
4.294
4.281
3.816
3.808
3.798
3.791
3.784
3.702
3.670
3.519
1.534
1.336
1.07
0.89
1.03
1.00
0.99
1.03
1.04
2.07
1.05
0.91
3.17
3.18
9.0
8.5
8.0
7.5
7.0
6.5
6.0
5.5
5.0
4.5
4.0
3.5
3.0
2.5
2.0
1.5
1.0
f1 (ppm)

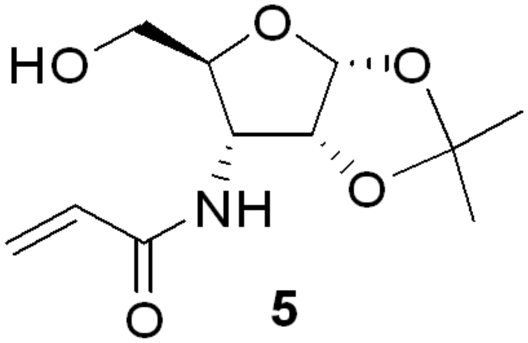
HO
O
O
O
NH
O

5

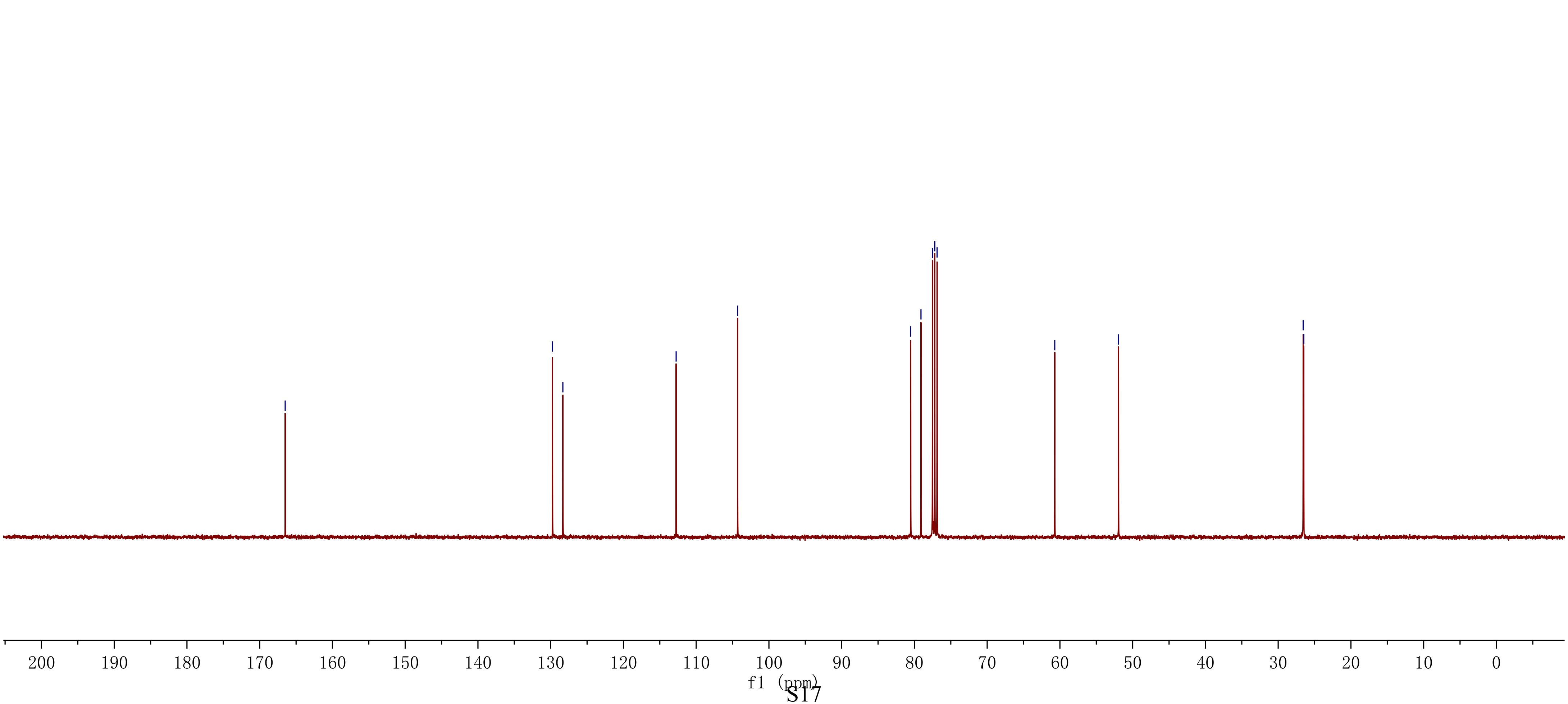
166.497
129.750
128.327
112.756
104.301
80.508
79.100
77.518
77.200
76.882
60.711
51.936
26.565
26.478
200 190 180 170 160 150 140 130 120 110 100 90 80 70 60 50 40 30 20 10 0
f1 (ppm)

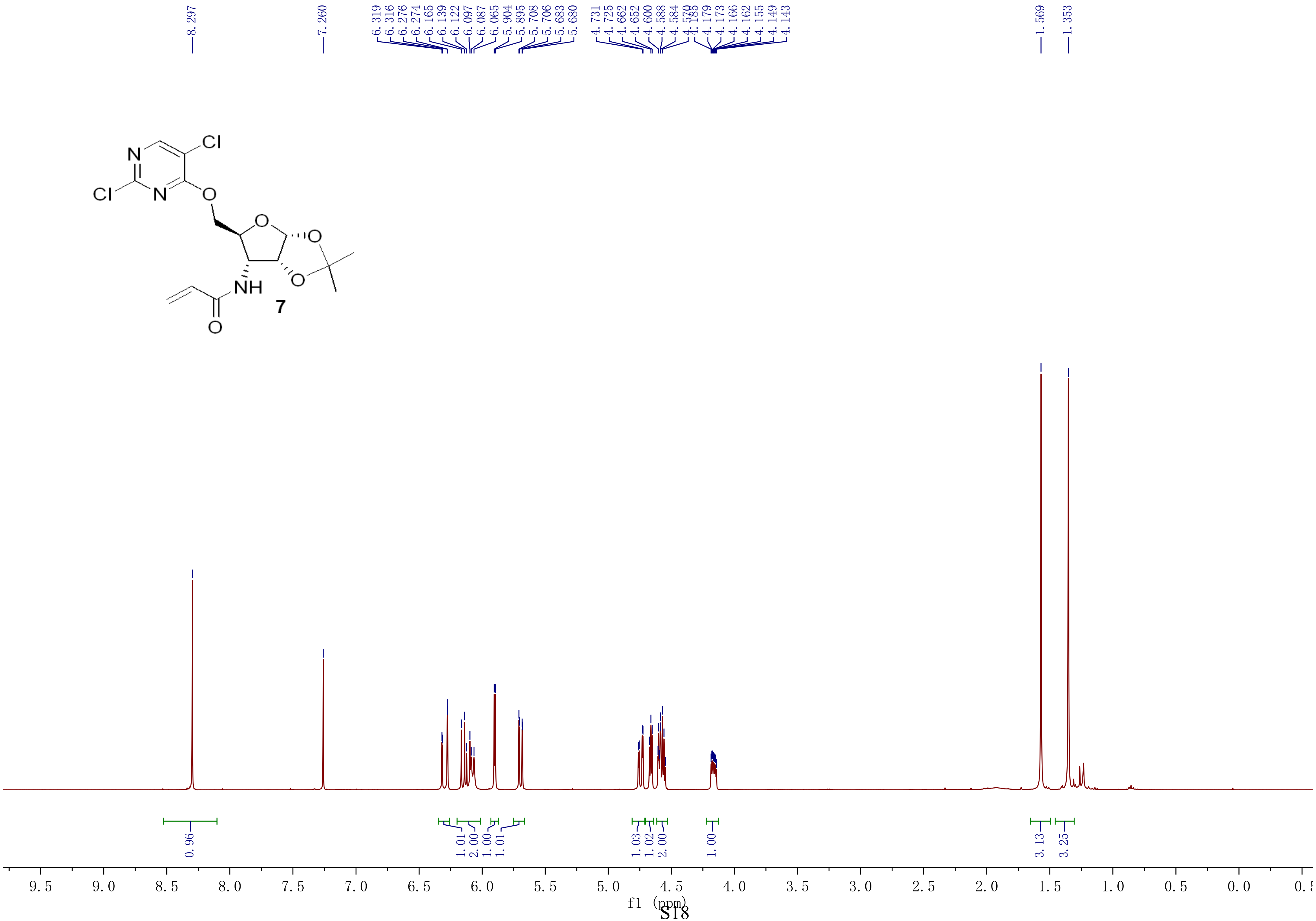
8.297
7.260
6.319
6.316
6.276
6.274
6.165
6.139
6.122
6.097
6.087
6.065
5.904
5.895
5.708
5.706
5.683
5.680
4.731
4.725
4.662
4.652
4.600
4.588
4.584
4.185
4.179
4.173
4.166
4.162
4.155
4.149
4.143
1.569
1.353
Cl
N
N
Cl
O
O
O
O
NH
O
7
0.96
1.01
2.00
1.00
1.01
1.03
1.02
2.00
1.00
3.13
3.25
9.5 9.0 8.5 8.0 7.5 7.0 6.5 6.0 5.5 5.0 4.5 4.0 3.5 3.0 2.5 2.0 1.5 1.0 0.5 0.0
f1 (ppm)

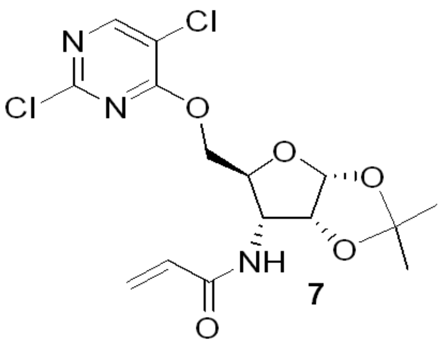
Cl
N
Cl
N
O
O
O
O
NH
O

7

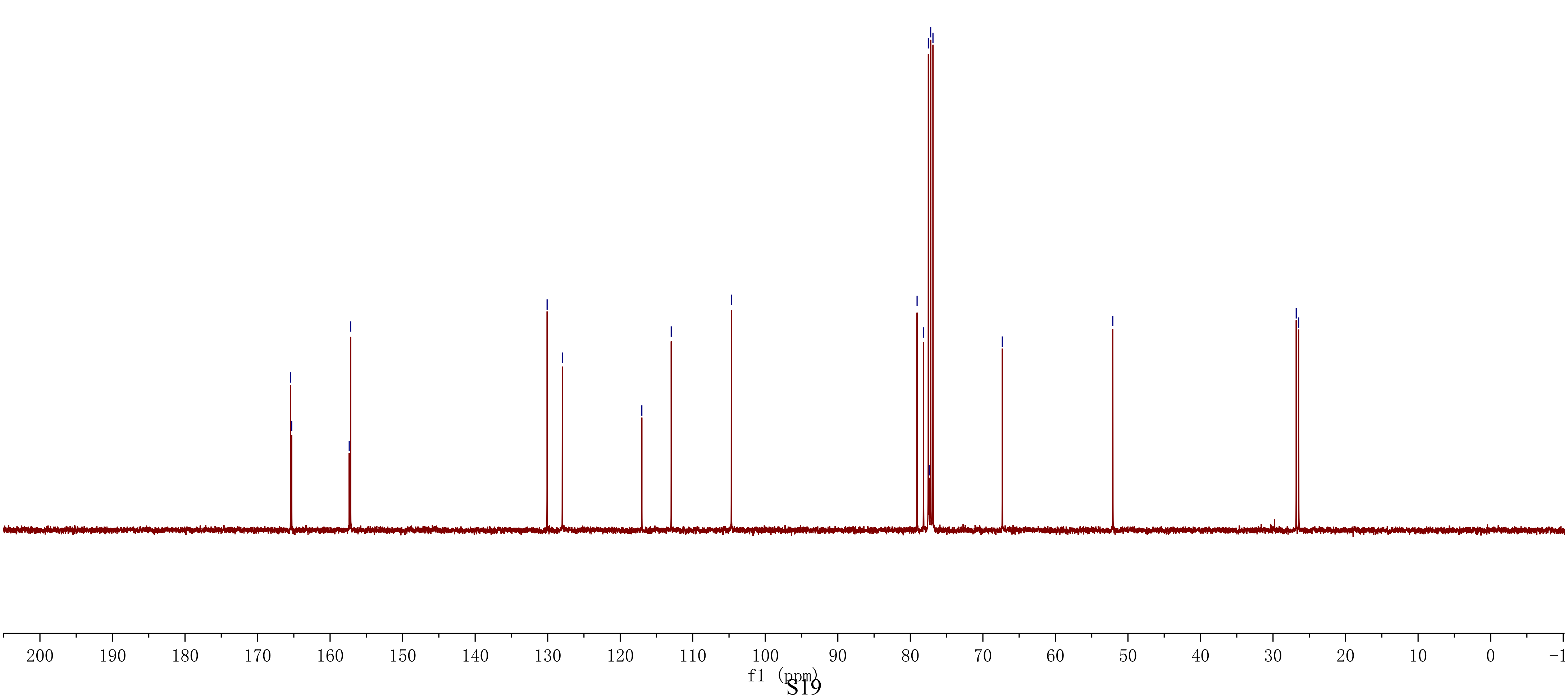
165.443
165.287
157.360
157.172
130.080
127.978
117.021
112.970
104.676
79.071
78.186
77.518
77.403
77.200
76.883
67.324
52.084
26.803
26.460
200 190 180 170 160 150 140 130 120 110 100 90 80 70 60 50 40 30 20 10 0 -1
f1 (ppm)

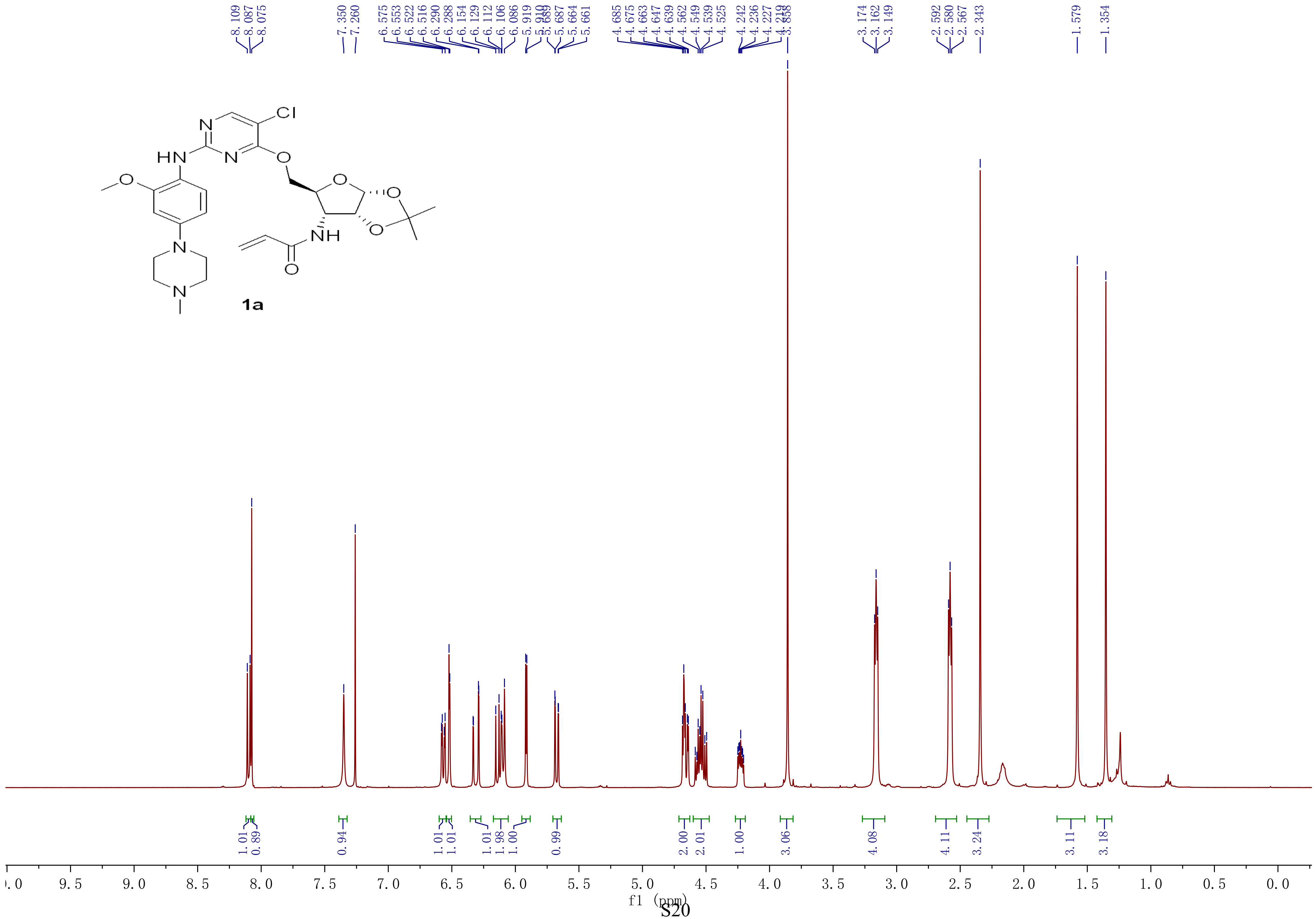

1a
f1 (ppm)

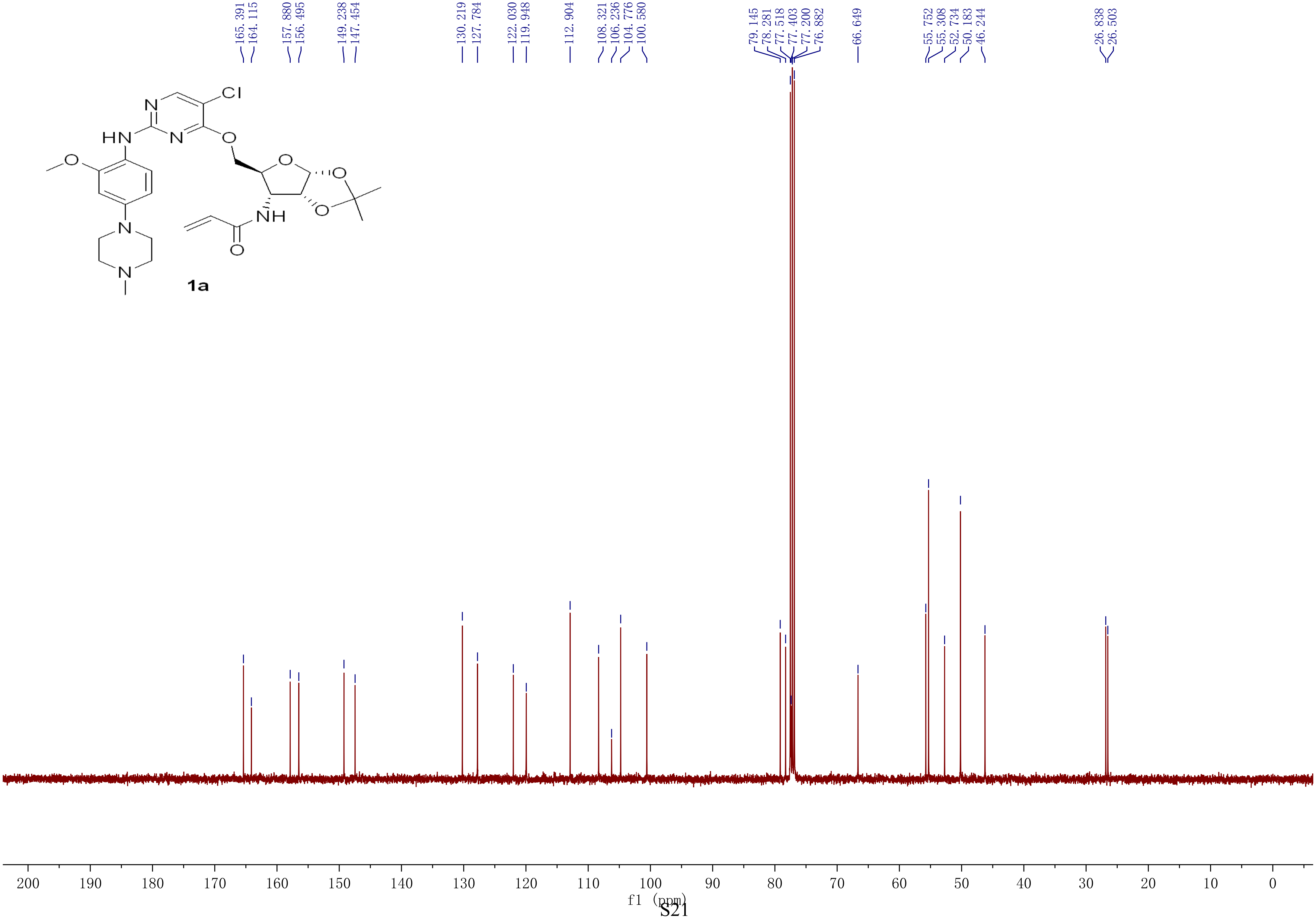
165.391
164.115
157.880
156.495
149.238
147.454
130.219
127.784
122.030
119.948
112.904
108.321
106.236
104.776
100.580
79.145
78.281
77.518
77.403
77.200
76.882
66.649
55.752
55.308
52.734
50.183
46.244
26.838
26.503
Cl
N
N
HN
O
O
O
O
O
NH
O
N
N
1a
200 190 180 170 160 150 140 130 120 110 100 90 80 70 60 50 40 30 20 10 0
f1 (ppm)

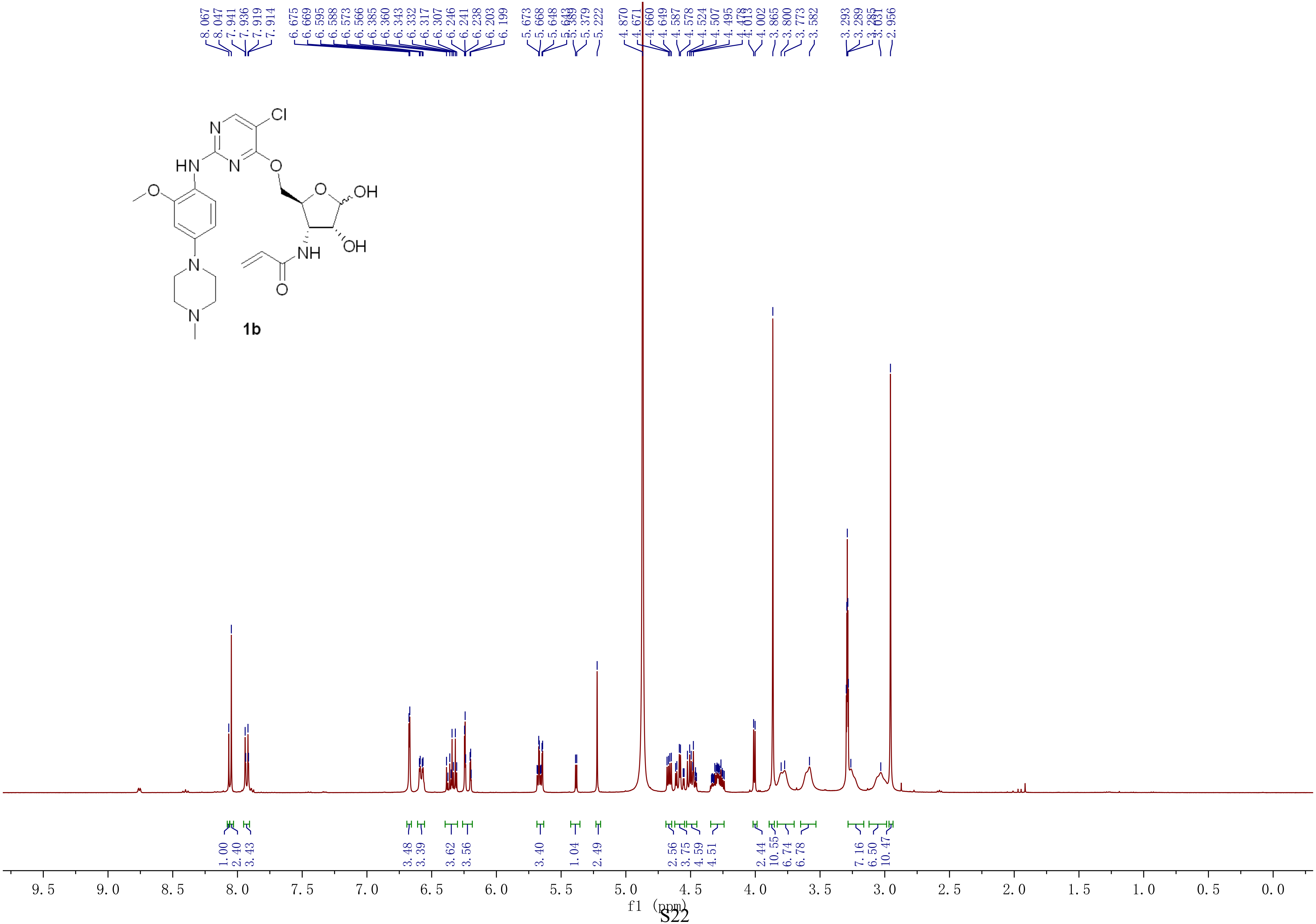
1b
f1 (ppm)

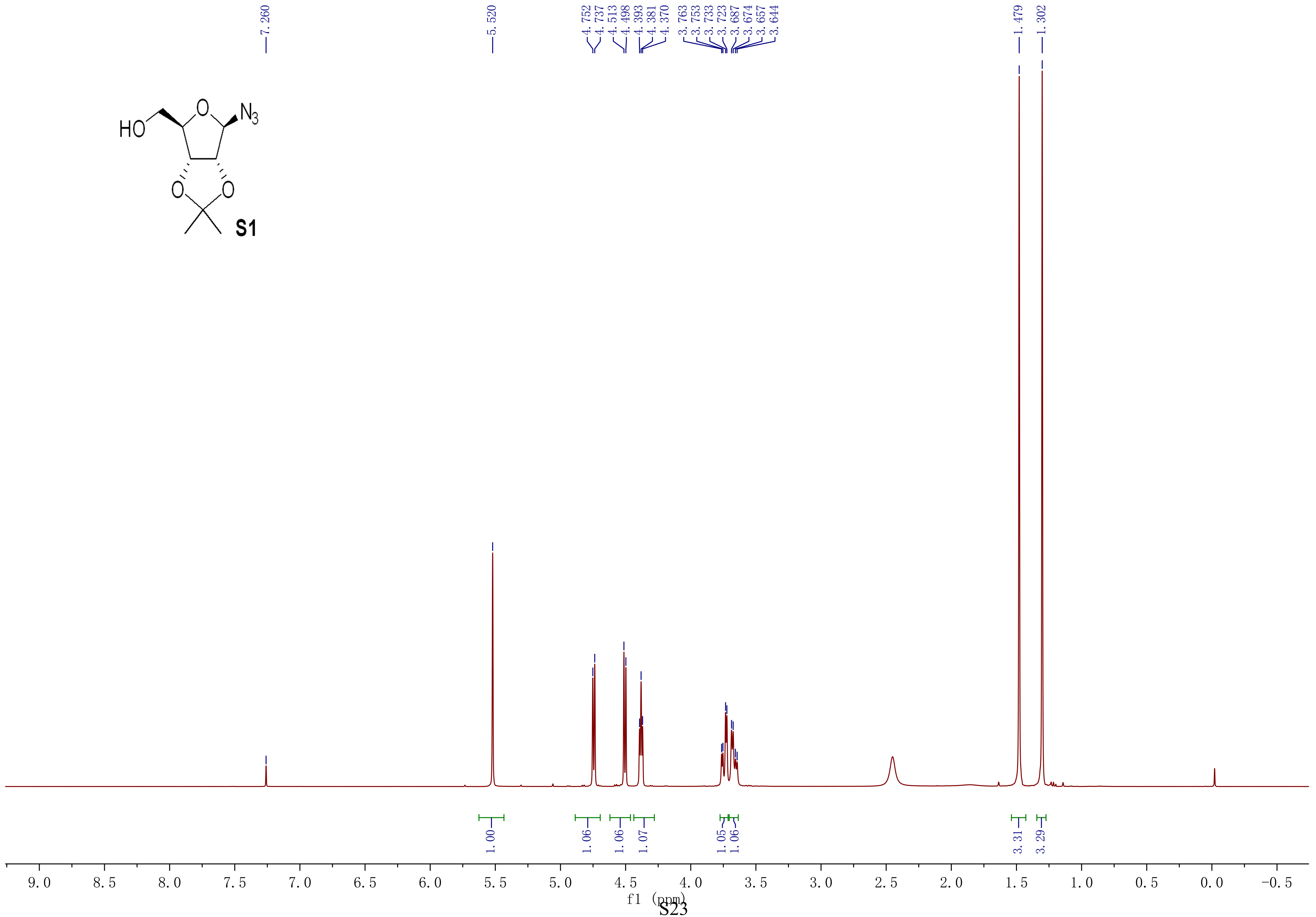

7.260
5.520
4.752
4.737
4.513
4.498
4.393
4.381
4.370
3.763
3.753
3.733
3.723
3.687
3.674
3.657
3.644
1.479
1.302
HO
O
N3
O
O
S1
1.00
1.06
1.06
1.07
1.05
1.06
3.31
3.29
9.0 8.5 8.0 7.5 7.0 6.5 6.0 5.5 5.0 4.5 4.0 3.5 3.0 2.5 2.0 1.5 1.0 0.5 0.0 −0.5
f1 (ppm)

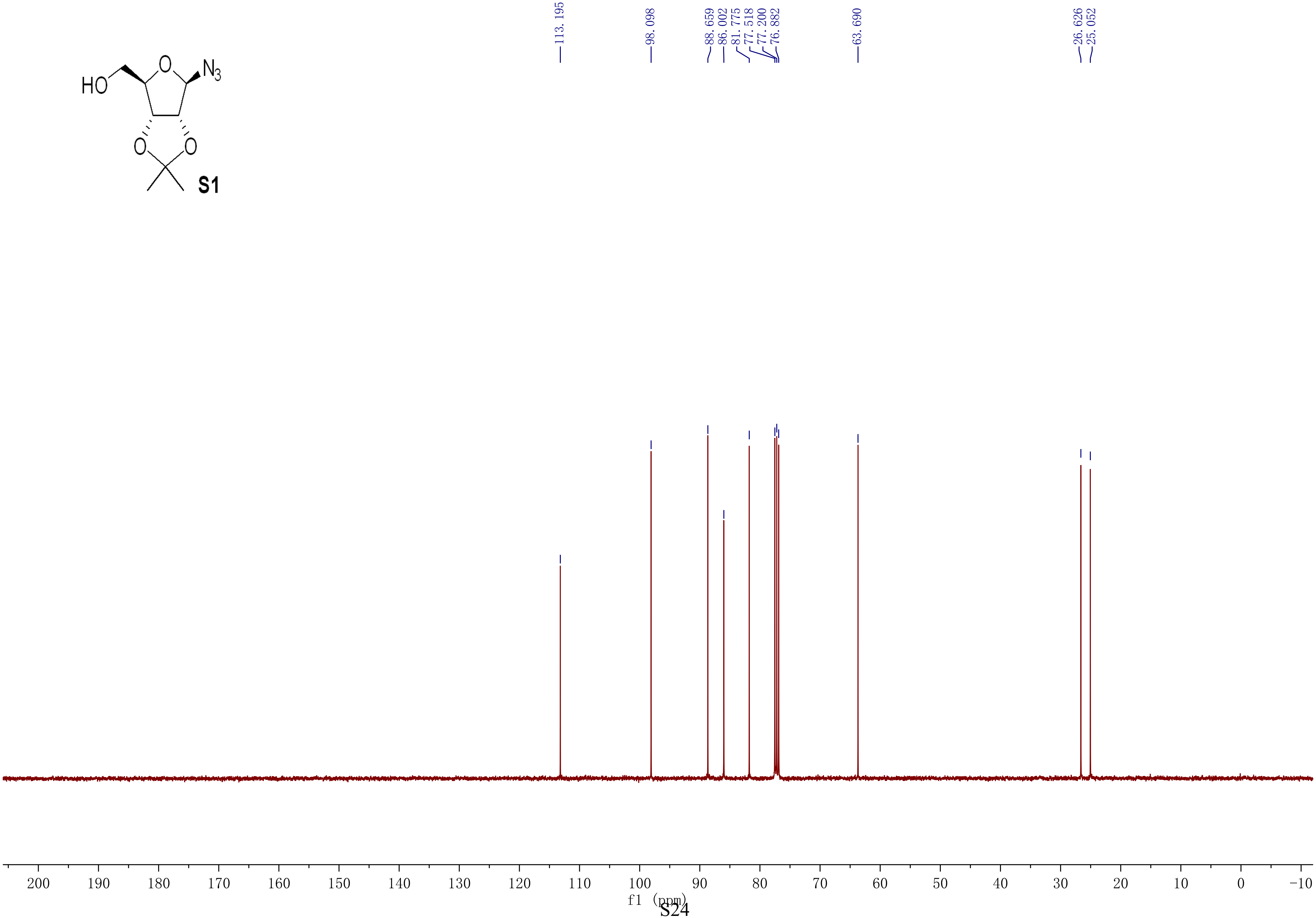
HO
O
N3
O
O
S1
113.195
98.098
88.659
86.002
81.775
77.518
77.200
76.882
63.690
26.626
25.052
200 190 180 170 160 150 140 130 120 110 100 90 80 70 60 50 40 30 20 10 0 −10
f1 (ppm)

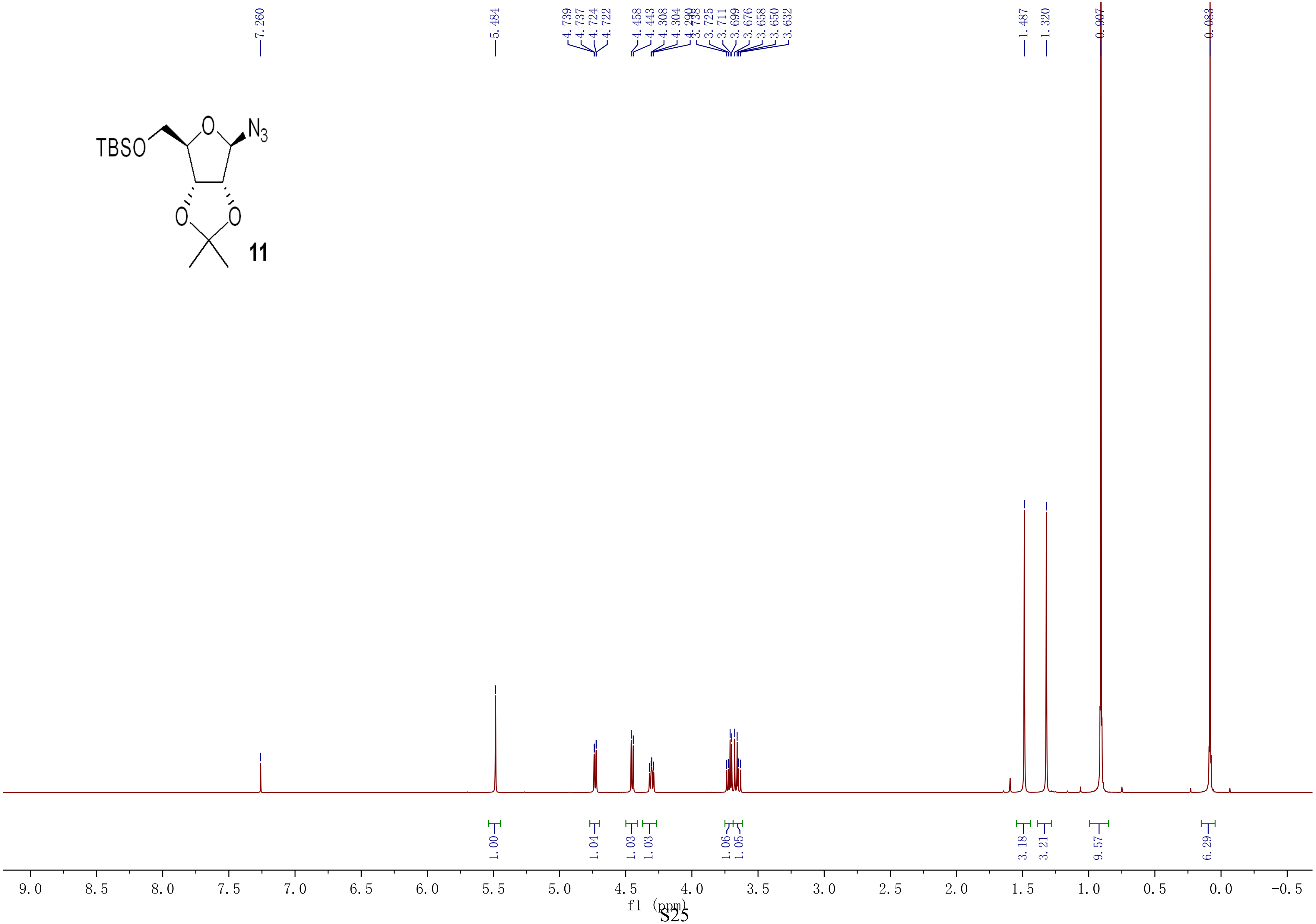
7.260
5.484
4.739
4.737
4.724
4.722
4.458
4.443
4.308
4.304
3.725
3.711
3.699
3.676
3.658
3.650
3.632
1.487
1.320
0.907
0.083
TBSO
O
N_3
O
O
11
1.00
1.04
1.03
1.03
1.06
1.05
3.18
3.21
9.57
6.29
9.0
8.5
8.0
7.5
7.0
6.5
6.0
5.5
5.0
4.5
4.0
3.5
3.0
2.5
2.0
1.5
1.0
0.5
0.0
-0.5
f1 (ppm)

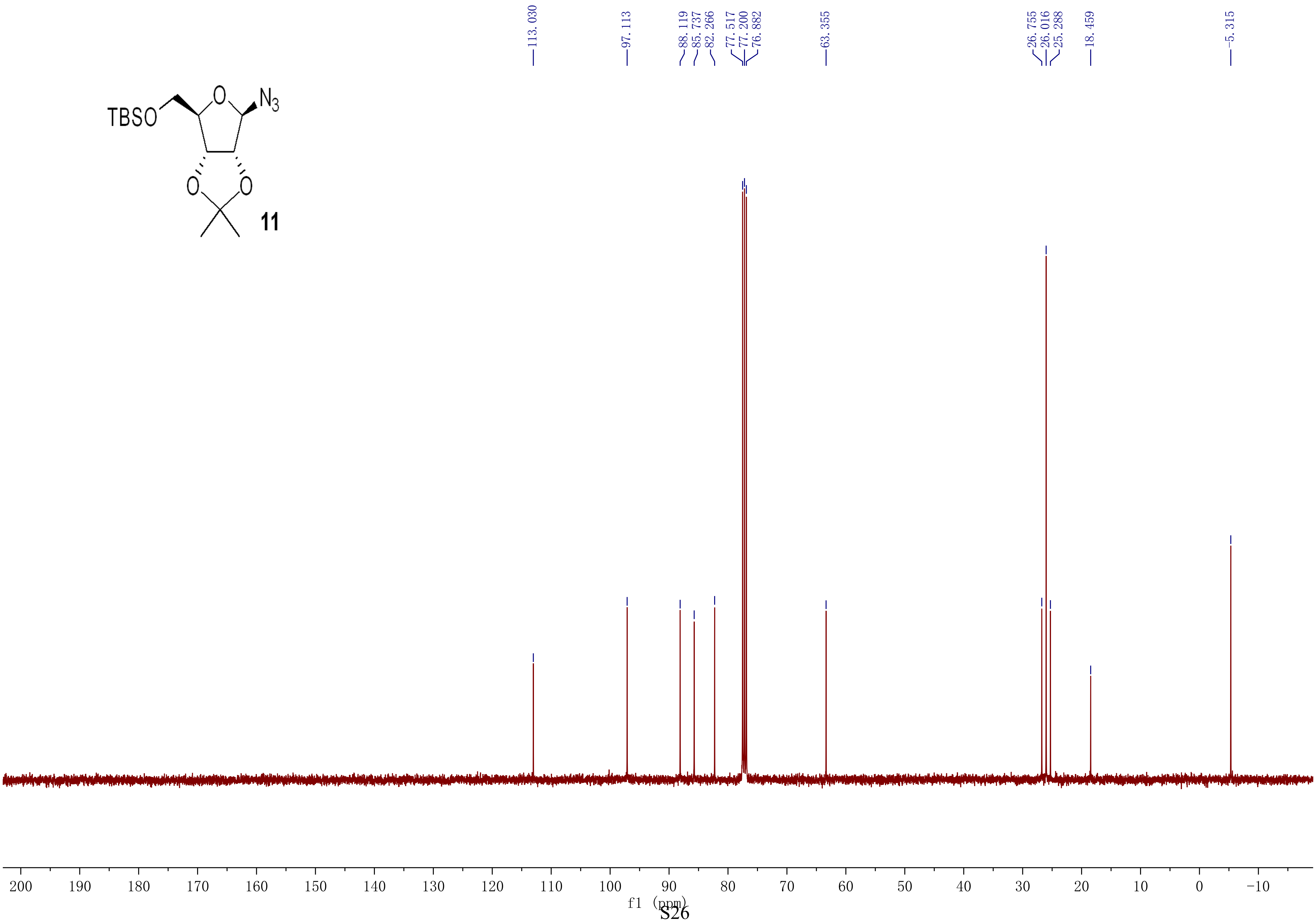
113.030
97.113
88.119
85.737
82.266
77.517
77.200
76.882
63.355
26.755
26.016
25.288
18.459
-5.315
TBSO
O
N3
O
O
11
200
190
180
170
160
150
140
130
120
110
100
90
80
70
60
50
40
30
20
10
0
-10
f1 (ppm)

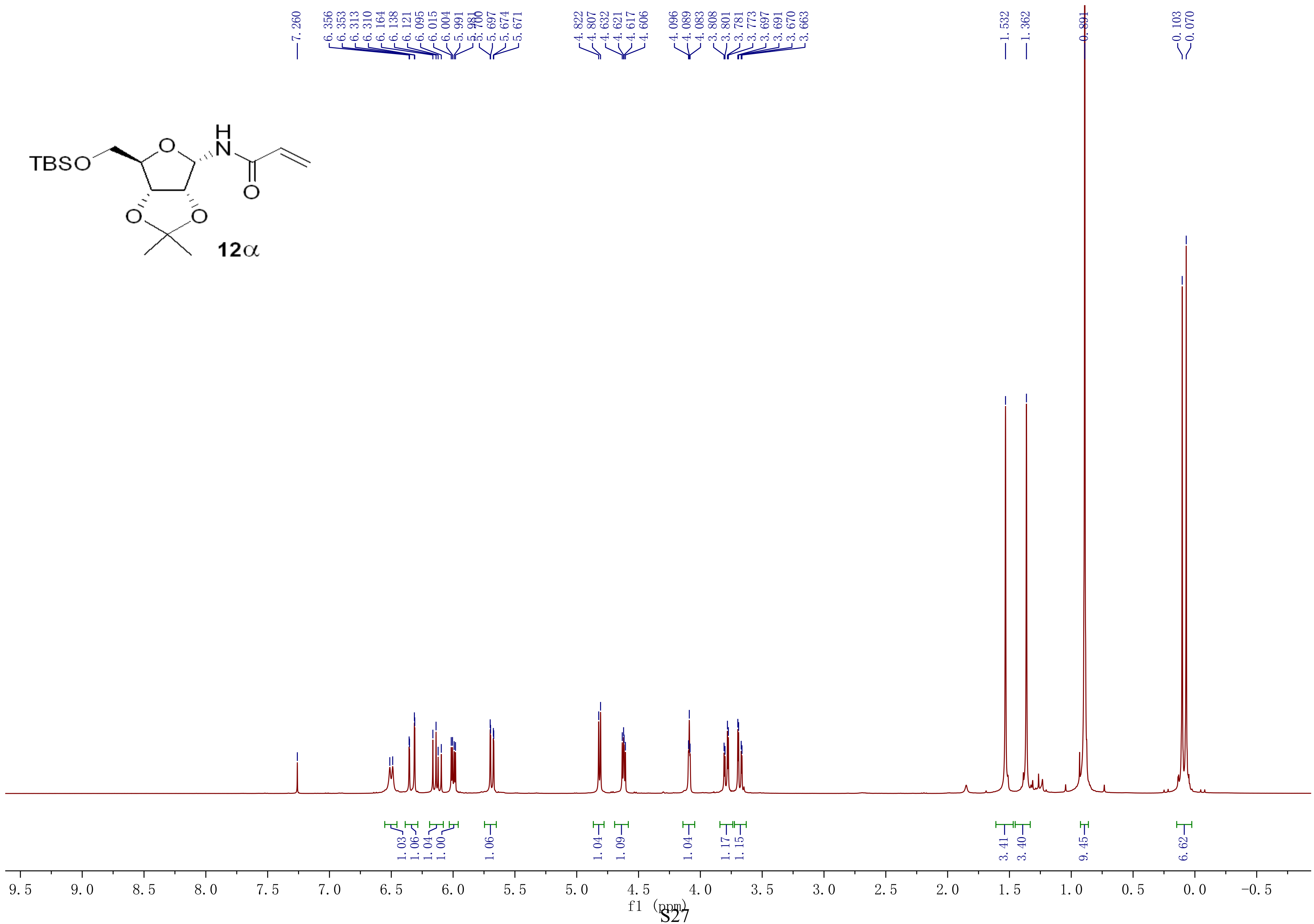
TBSO
12α
f1 (ppm)

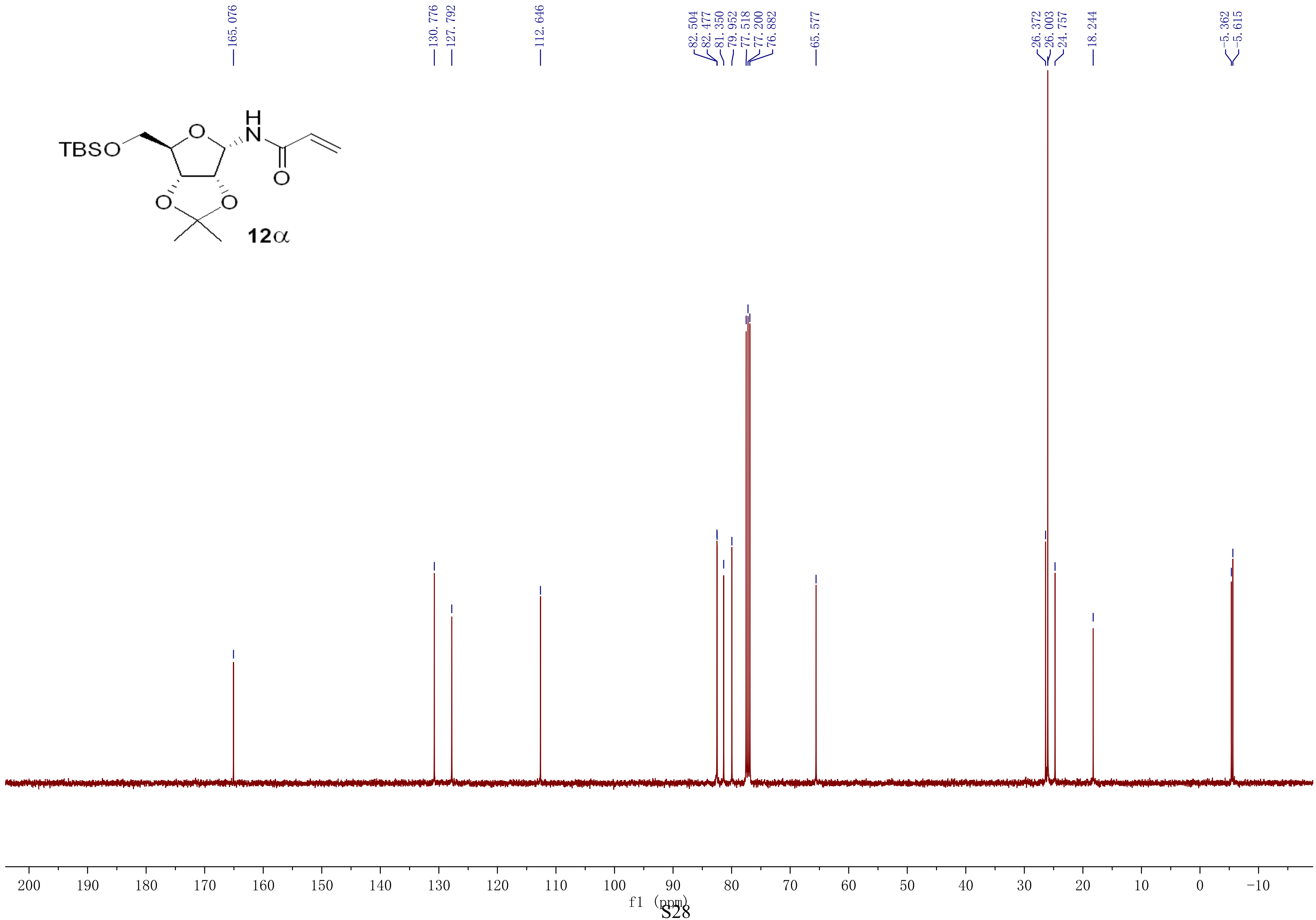
165.076
130.776
127.792
112.646
82.504
82.477
81.350
79.952
77.518
77.200
76.882
65.577
26.372
26.003
24.757
18.244
-5.362
-5.615
TBSO
H
N
O
12α
200 190 180 170 160 150 140 130 120 110 100 90 80 70 60 50 40 30 20 10 0 -10
f1 (ppm)

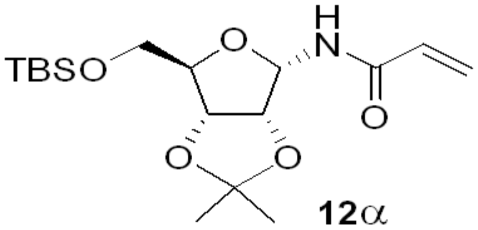
TBSO
H
N
O
O
O
O

12α

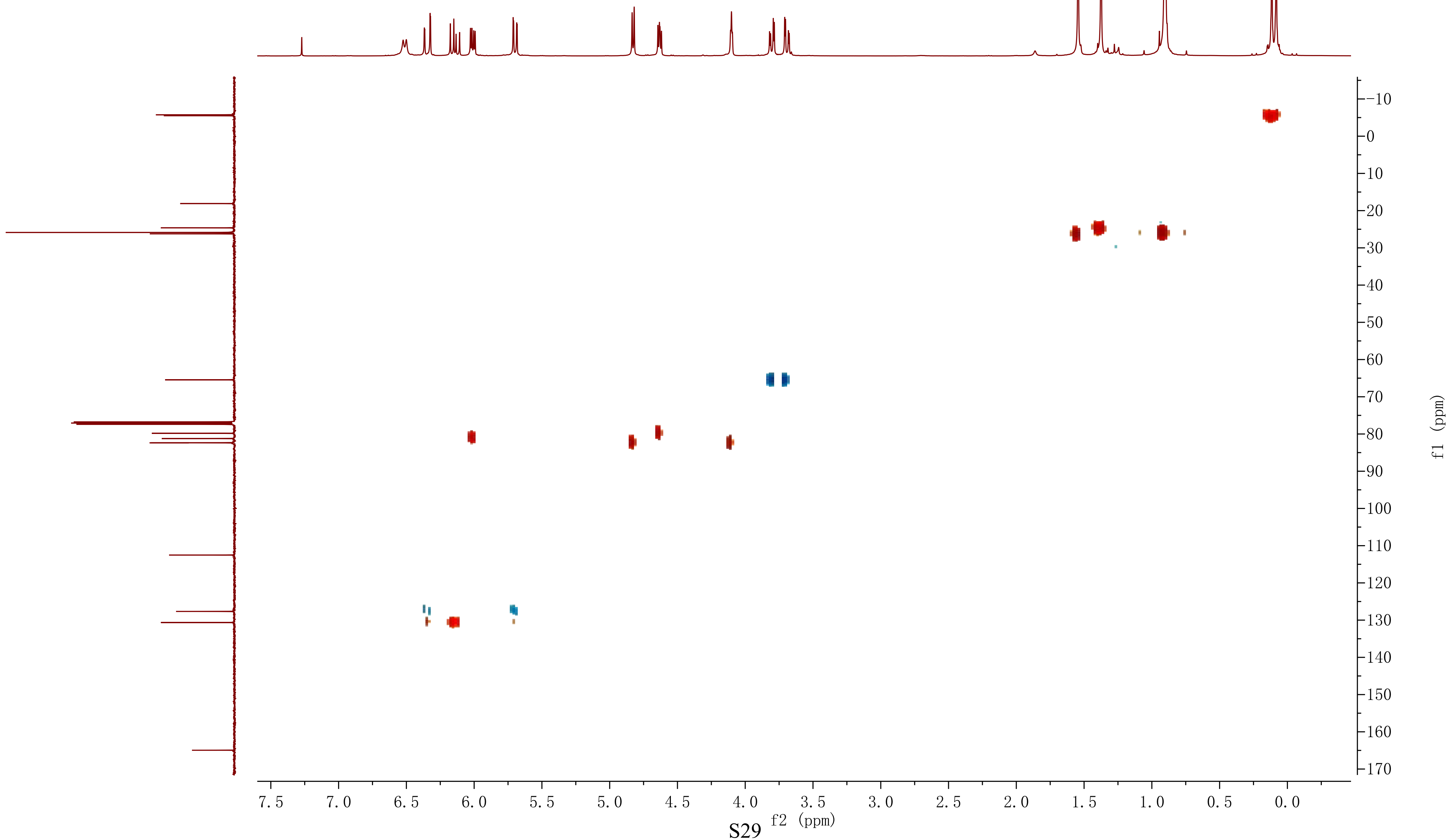
7.5
7.0
6.5
6.0
5.5
5.0
4.5
4.0
3.5
3.0
2.5
2.0
1.5
1.0
0.5
0.0
f2 (ppm)
-10
0
10
20
30
40
50
60
70
80
90
100
110
120
130
140
150
160
170
f1 (ppm)

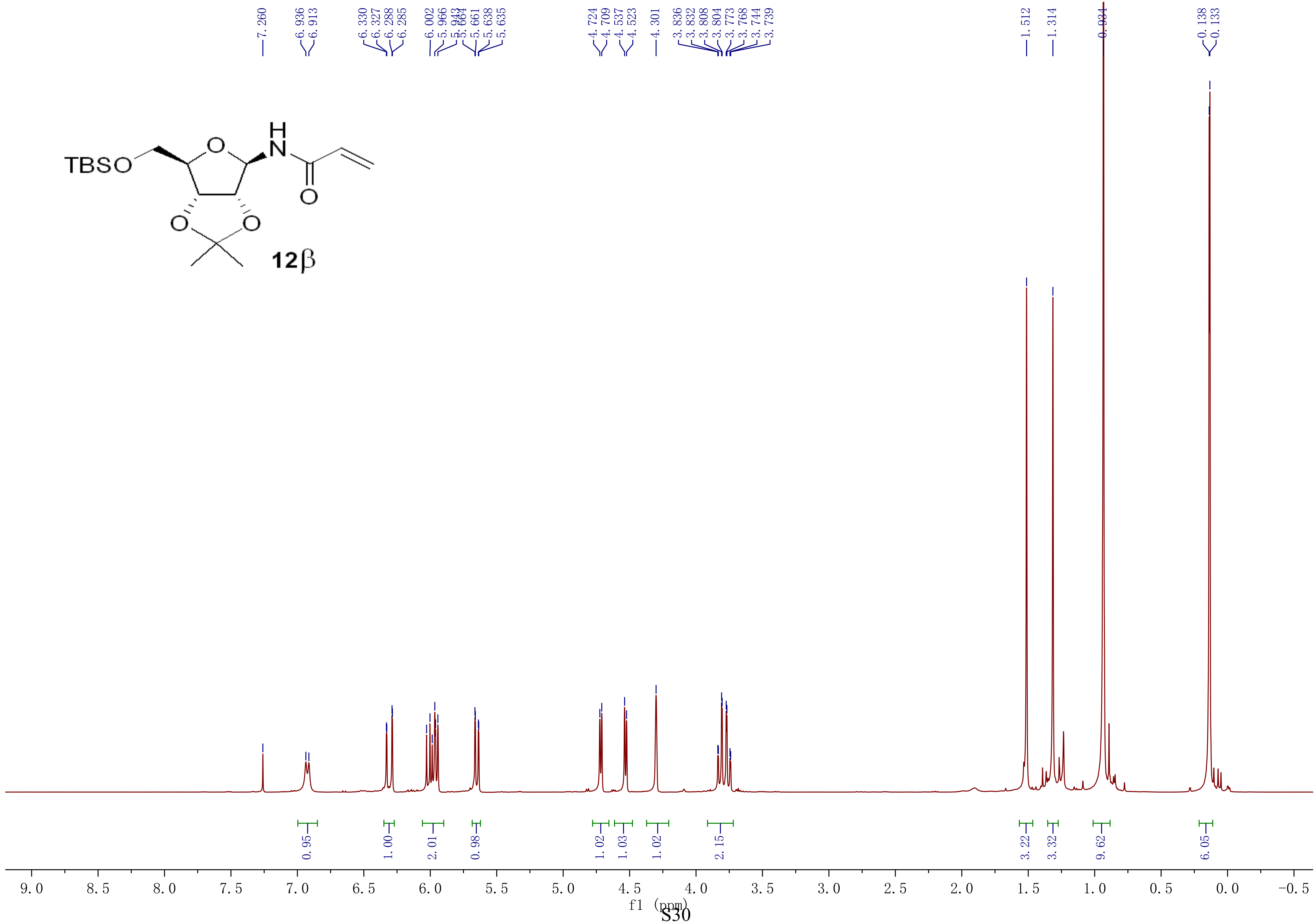

TBSO
H
N
O
12β
7.260
6.936
6.913
6.330
6.327
6.288
6.285
6.002
5.966
5.943
5.664
5.661
5.638
5.635
4.724
4.709
4.537
4.523
4.301
3.836
3.832
3.808
3.804
3.773
3.768
3.744
3.739
1.512
1.314
0.934
0.138
0.133
0.95
1.00
2.01
0.98
1.02
1.03
1.02
2.15
3.22
3.32
9.62
6.05
9.0 8.5 8.0 7.5 7.0 6.5 6.0 5.5 5.0 4.5 4.0 3.5 3.0 2.5 2.0 1.5 1.0 0.5 0.0 -0.5
f1 (ppm)

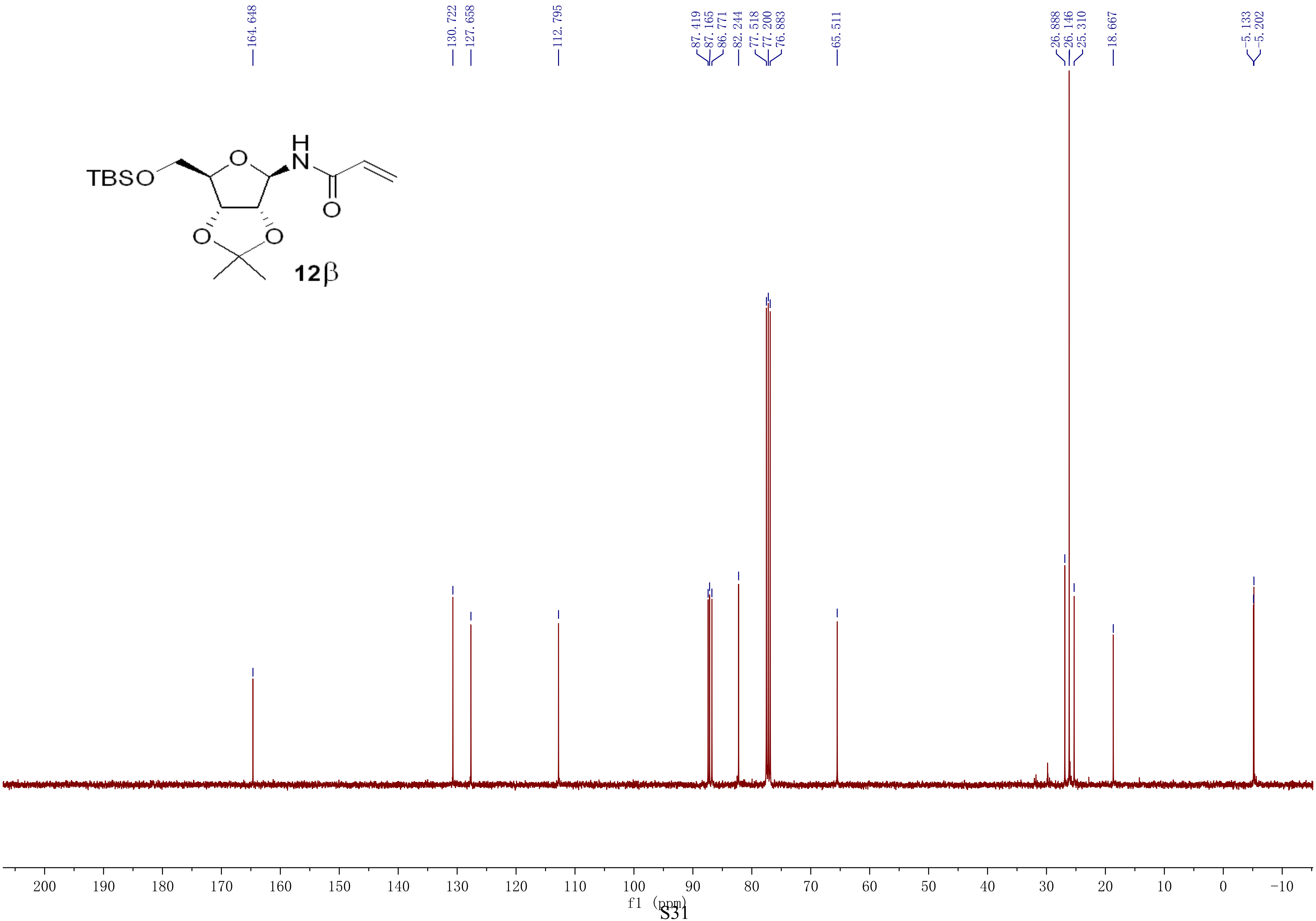

TBSO
O
N
H
O
O
O
12β
164.648
130.722
127.658
112.795
87.419
87.165
86.771
82.244
77.518
77.200
76.883
65.511
26.888
26.146
25.310
18.667
-5.133
-5.202
200 190 180 170 160 150 140 130 120 110 100 90 80 70 60 50 40 30 20 10 0 -10
f1 (ppm)

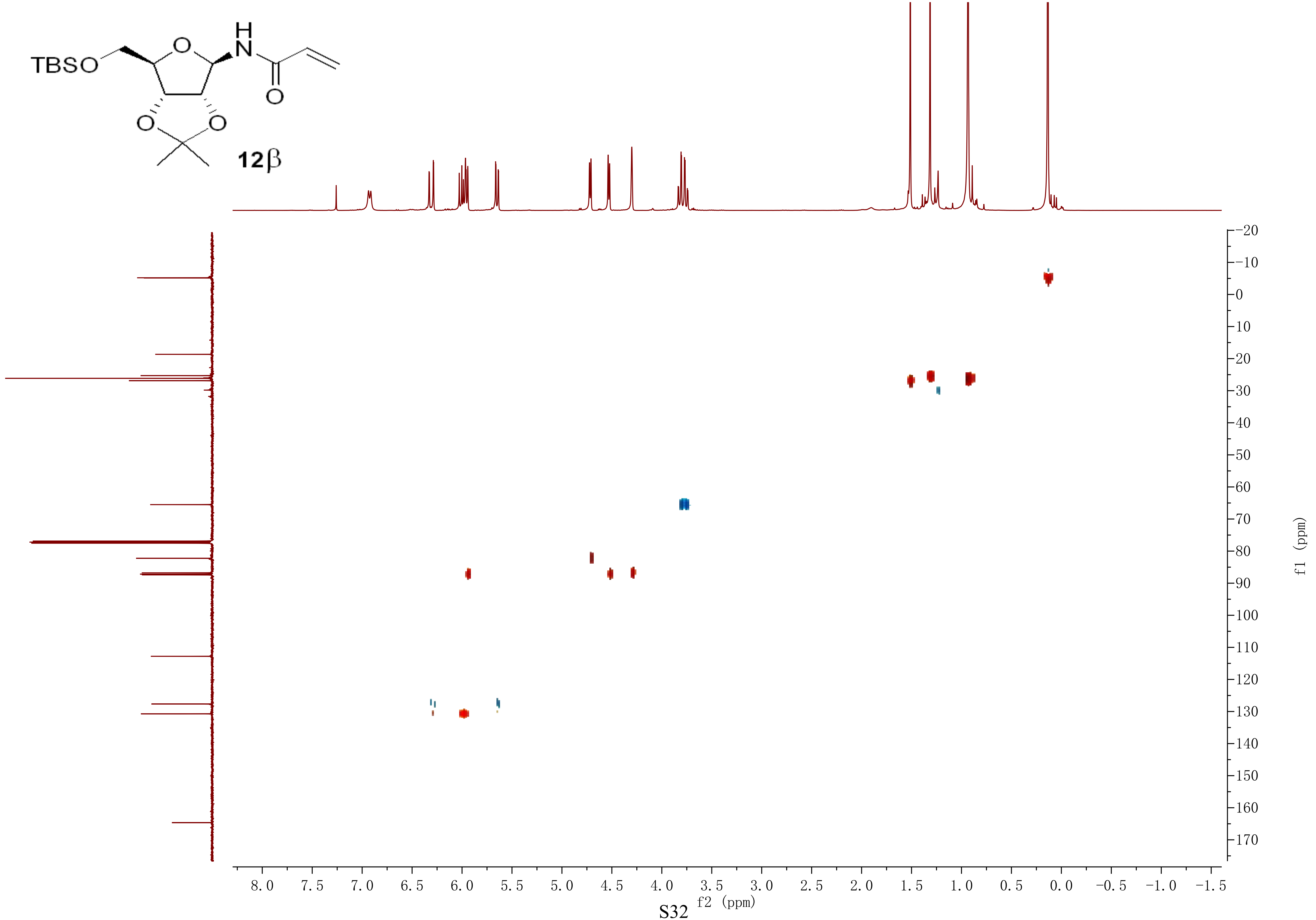

12β
f2 (ppm)
f1 (ppm)

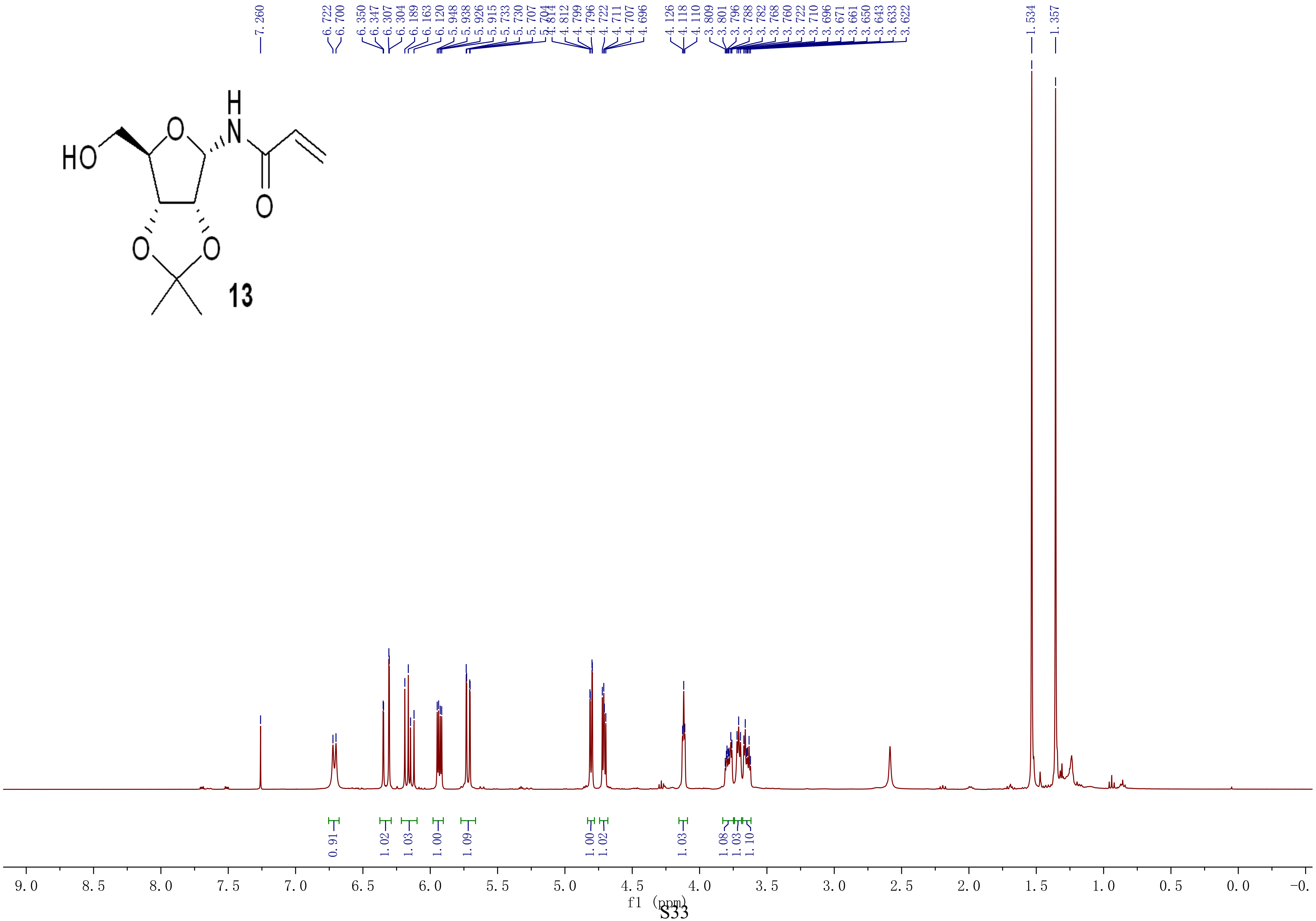

7.260
6.722
6.700
6.350
6.347
6.307
6.304
6.189
6.163
6.120
5.948
5.938
5.926
5.915
5.733
5.730
5.707
5.704
4.814
4.812
4.799
4.796
4.722
4.711
4.707
4.696
4.126
4.118
4.110
3.809
3.801
3.796
3.788
3.782
3.768
3.760
3.722
3.710
3.696
3.671
3.661
3.650
3.643
3.633
3.622
1.534
1.357
13
0.91
1.02
1.03
1.00
1.09
1.00
1.02
1.03
1.08
1.03
1.10
9.0
8.5
8.0
7.5
7.0
6.5
6.0
5.5
5.0
4.5
4.0
3.5
3.0
2.5
2.0
1.5
1.0
0.5
0.0
f1 (ppm)

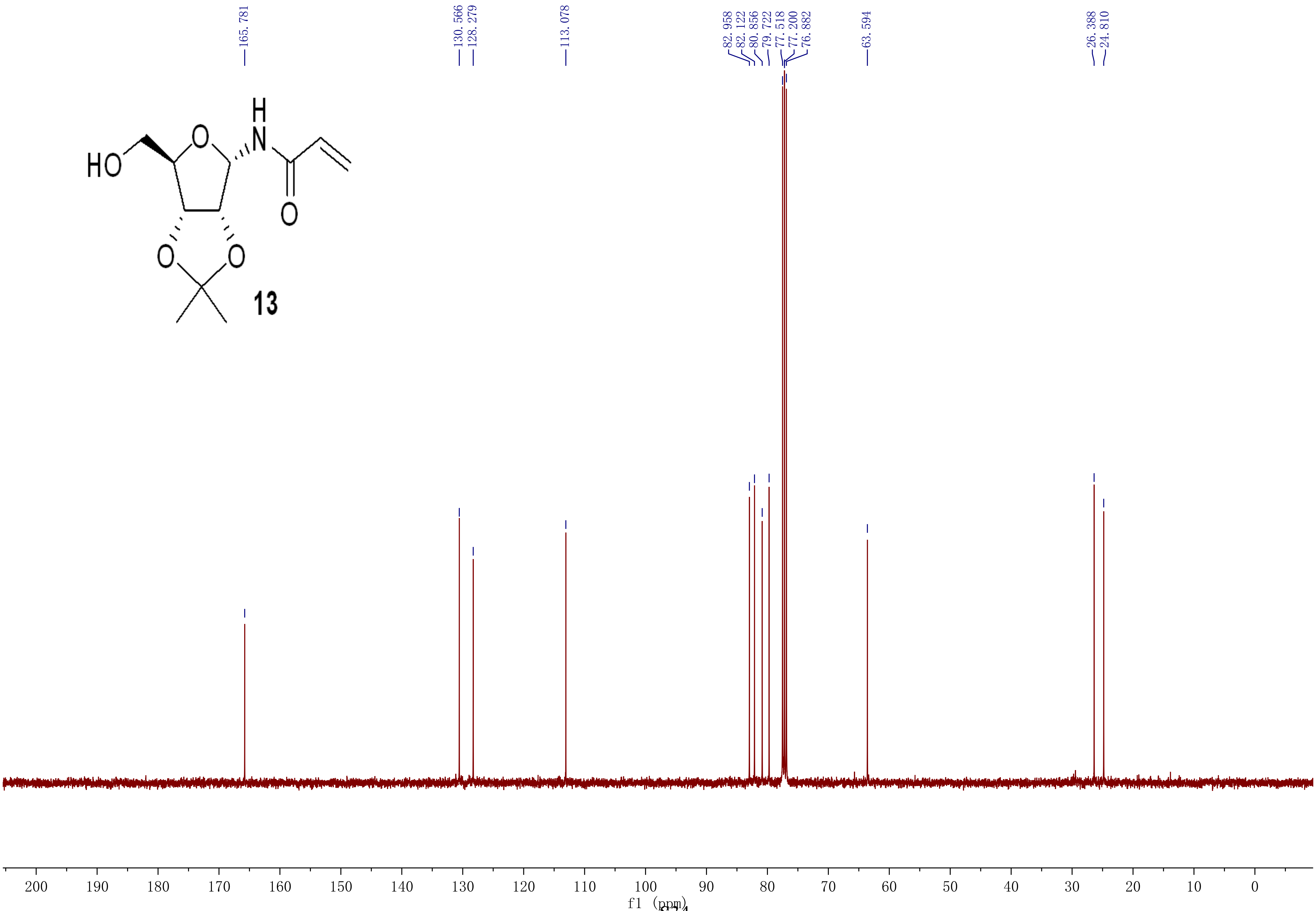
165.781
130.566
128.279
113.078
82.958
82.122
80.856
79.722
77.518
77.200
76.882
63.594
26.388
24.810
HO
O
H
N
O
O
O
13
200
190
180
170
160
150
140
130
120
110
100
90
80
70
60
50
40
30
20
10
0
f1 (ppm)

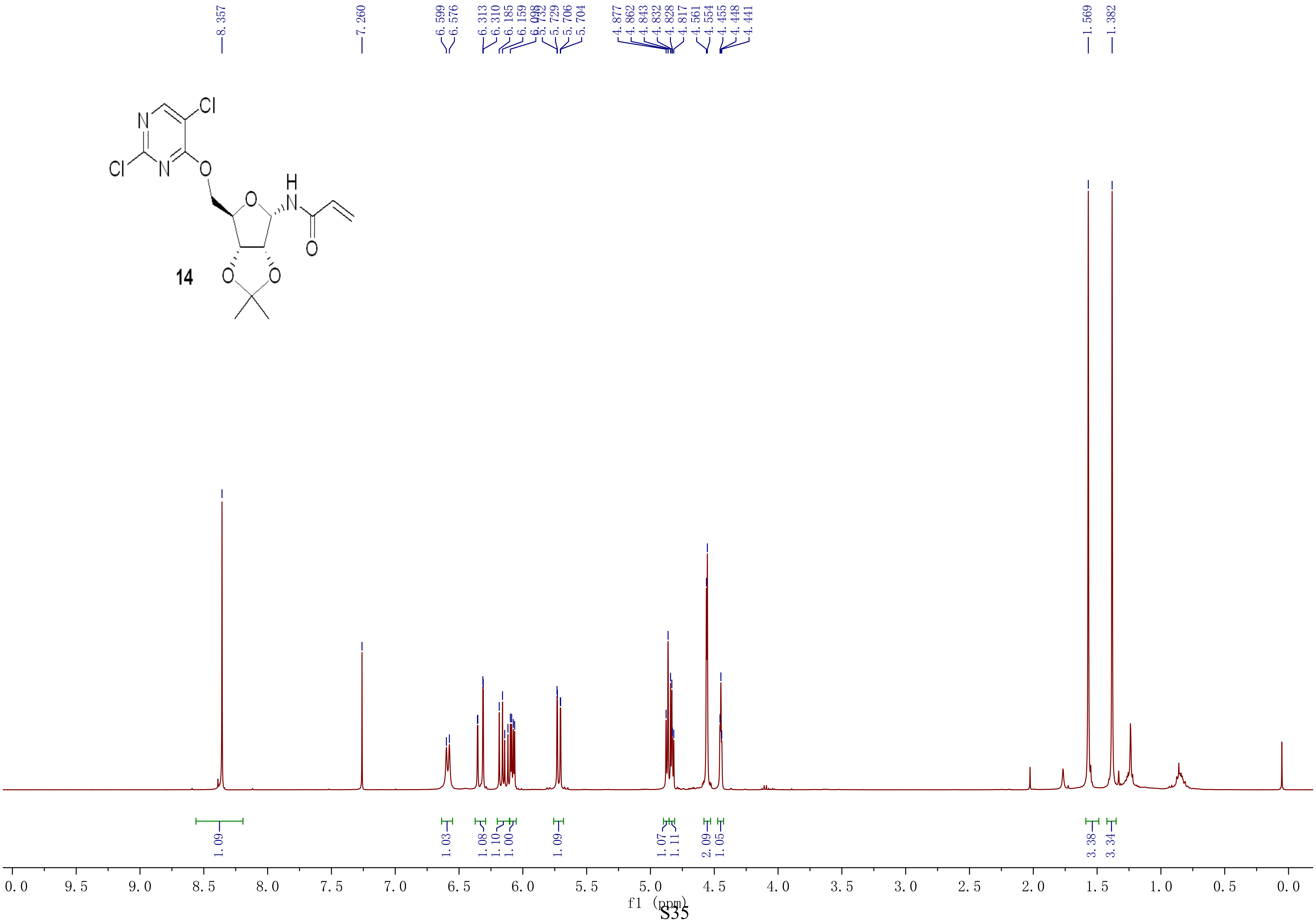

8.357
7.260
6.599
6.576
6.313
6.310
6.185
6.159
5.729
5.706
5.704
4.877
4.862
4.843
4.832
4.828
4.817
4.561
4.554
4.455
4.448
4.441
1.569
1.382
14
1.09
1.03
1.08
1.10
1.00
1.09
1.07
1.11
2.09
1.05
3.38
3.34
f1 (ppm)

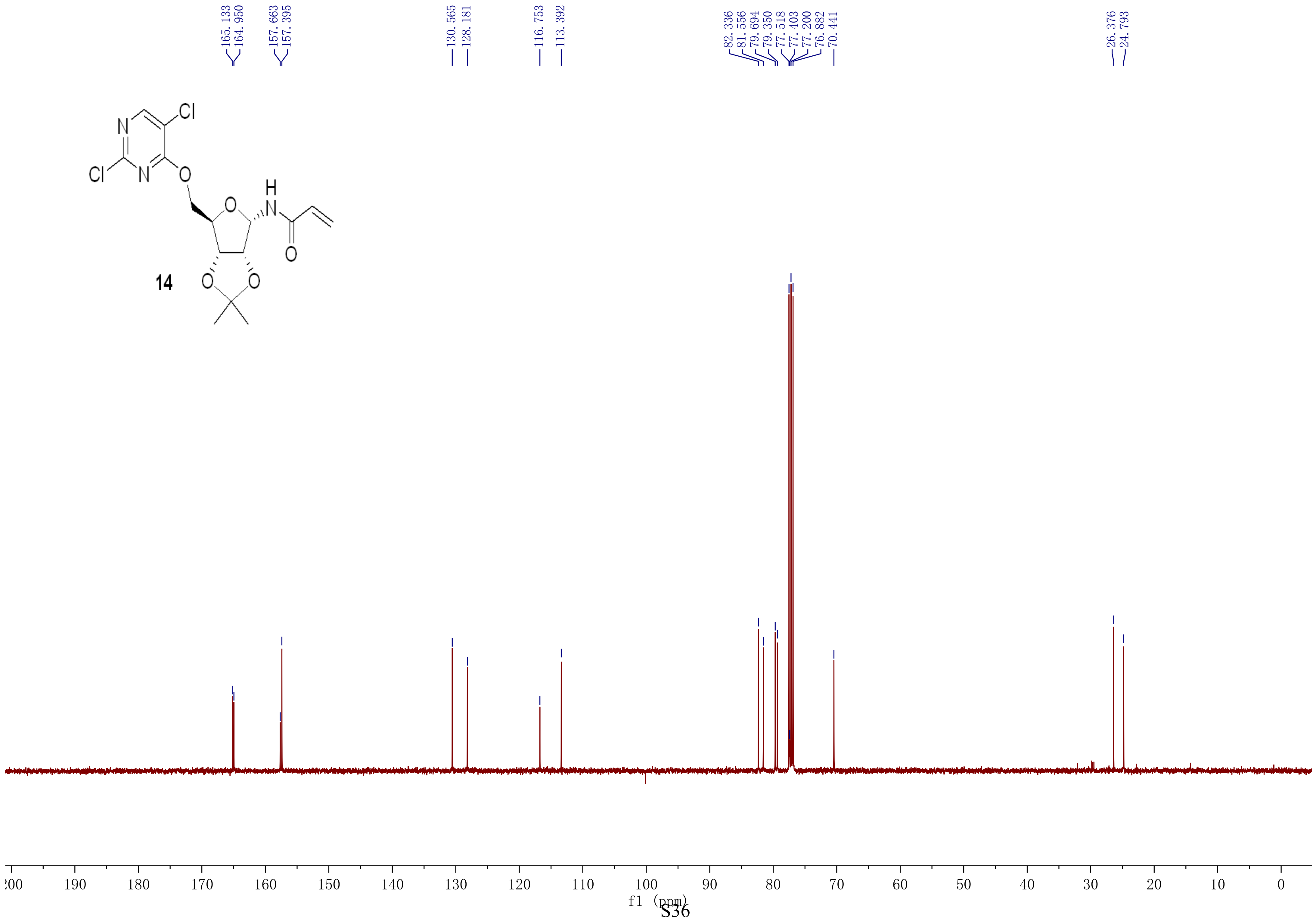

165.133
164.950
157.663
157.395
130.565
128.181
116.753
113.392
82.336
81.556
79.694
79.350
77.518
77.403
77.200
76.882
70.441
26.376
24.793
14
f1 (ppm)

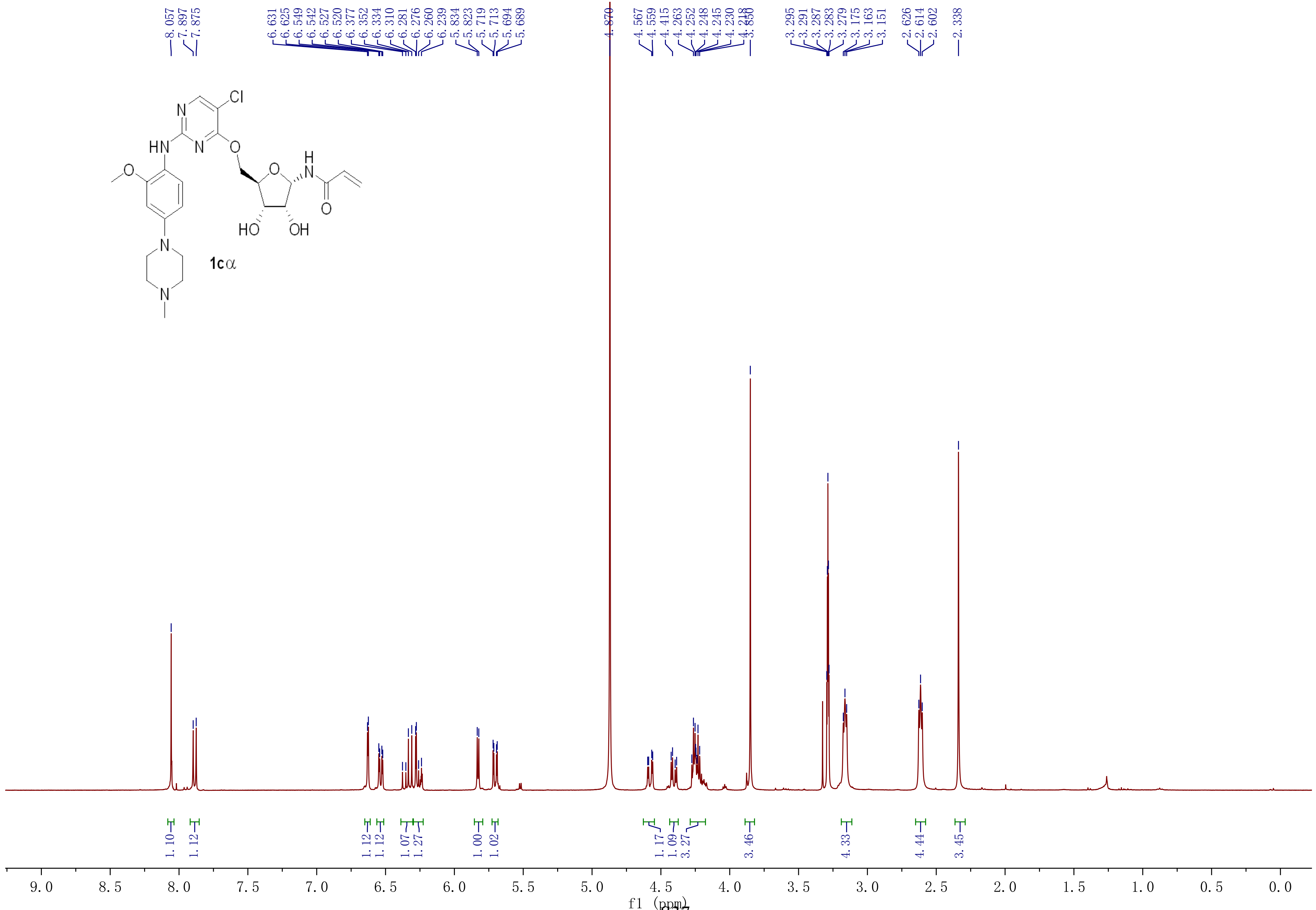
8.057
7.897
7.875
6.631
6.625
6.549
6.542
6.527
6.520
6.377
6.352
6.334
6.310
6.281
6.276
6.260
6.239
5.834
5.823
5.719
5.713
5.694
5.689
4.870
4.567
4.559
4.415
4.263
4.252
4.248
4.245
4.230
4.218
3.850
3.295
3.291
3.287
3.283
3.279
3.175
3.163
3.151
2.626
2.614
2.602
2.338
Cl
N
HN
N
O
O
H
N
O
HO
OH
N
N
1cα
1.10
1.12
1.12
1.12
1.07
1.27
1.00
1.02
1.17
1.09
3.27
3.46
4.33
4.44
3.45
9.0
8.5
8.0
7.5
7.0
6.5
6.0
5.5
5.0
4.5
4.0
3.5
3.0
2.5
2.0
1.5
1.0
0.5
0.0
f1 (ppm)

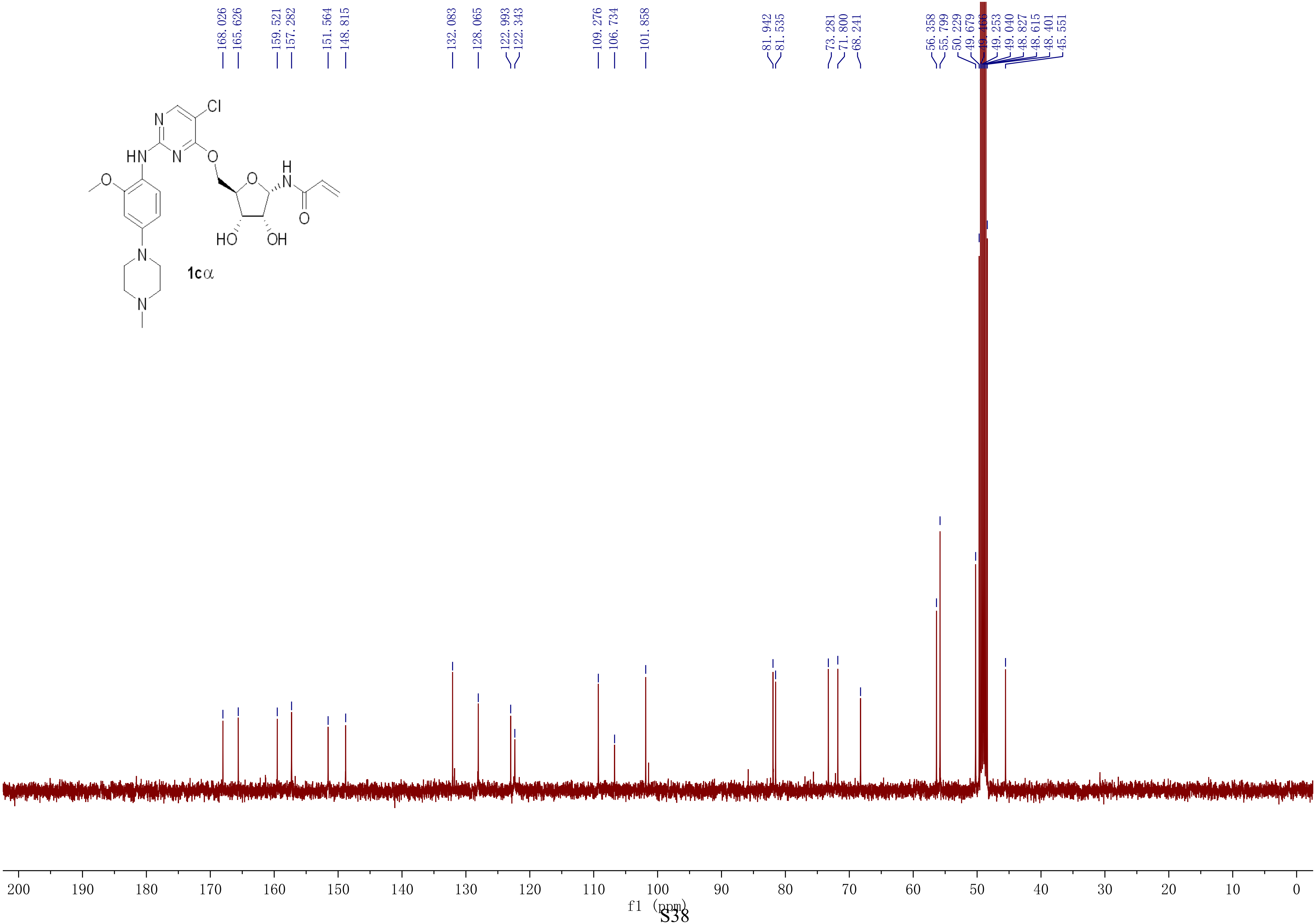
168.026
165.626
159.521
157.282
151.564
148.815
132.083
128.065
122.993
122.343
109.276
106.734
101.858
81.942
81.535
73.281
71.800
68.241
56.358
55.799
50.229
49.679
49.253
49.040
48.827
48.615
48.401
45.551
1cα
200 190 180 170 160 150 140 130 120 110 100 90 80 70 60 50 40 30 20 10 0
f1 (ppm)

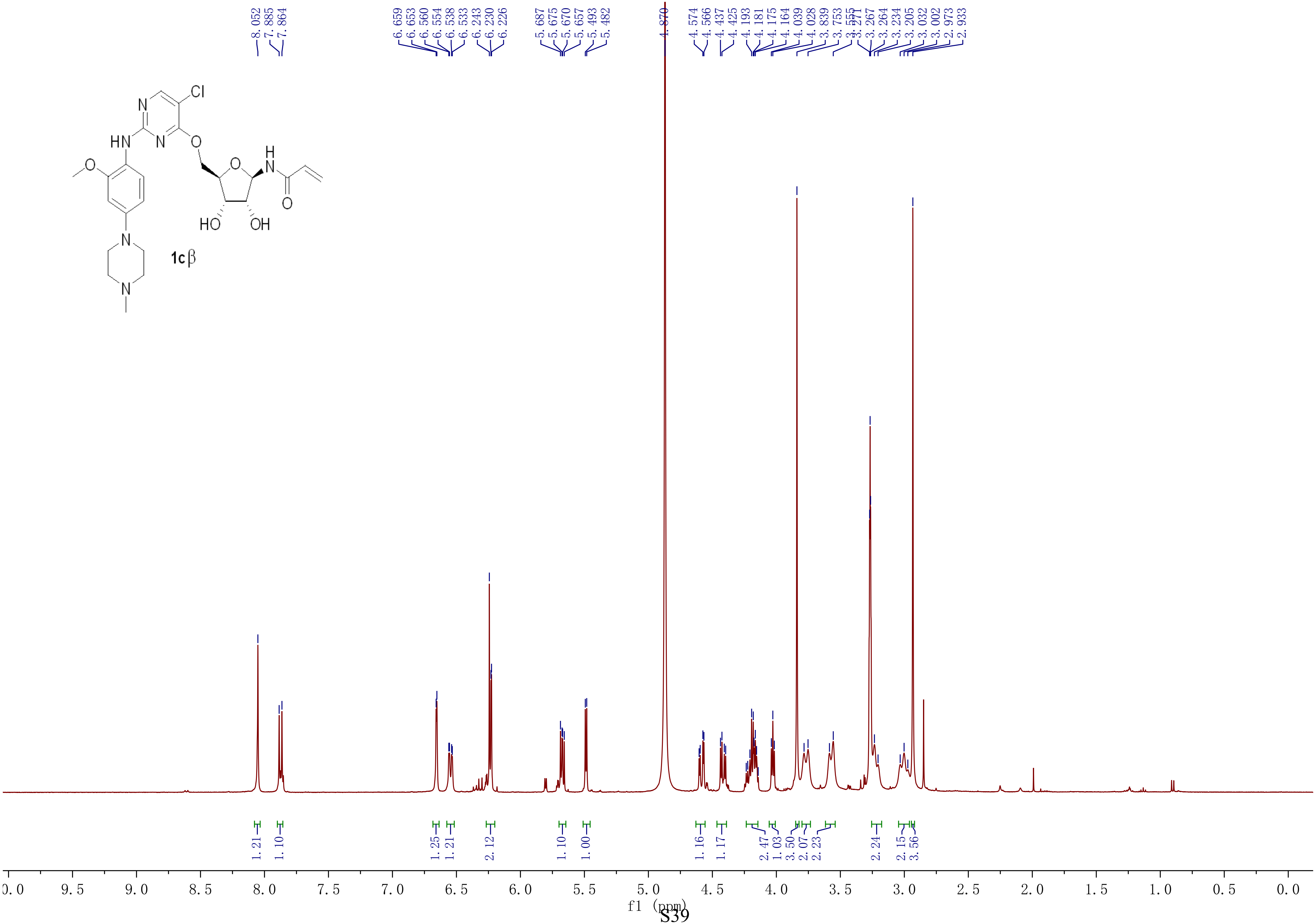

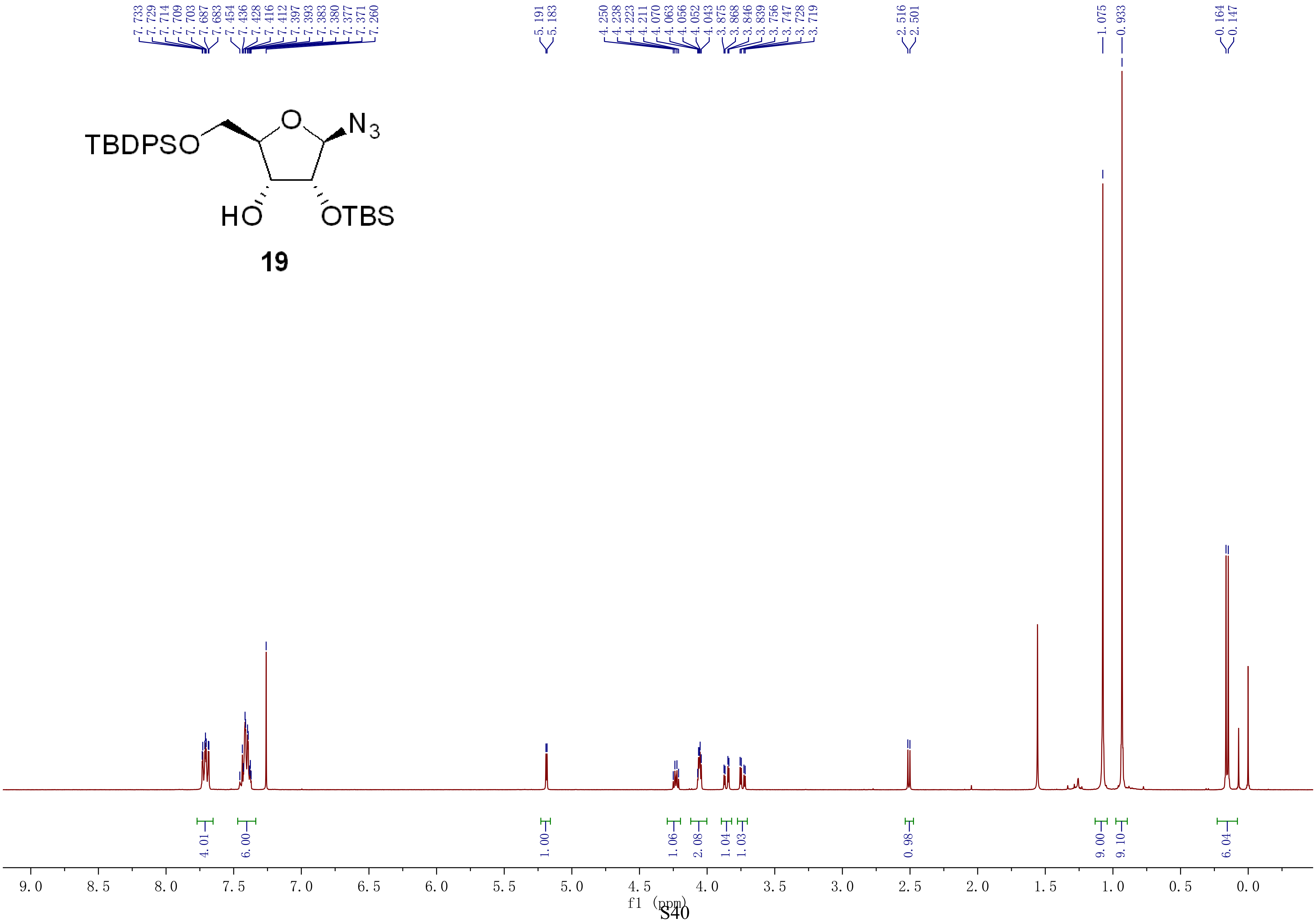

7.733
7.729
7.714
7.709
7.703
7.687
7.683
7.454
7.436
7.428
7.416
7.412
7.397
7.393
7.383
7.380
7.377
7.371
7.260
5.191
5.183
4.250
4.238
4.223
4.211
4.070
4.063
4.056
4.052
4.043
3.875
3.868
3.846
3.839
3.756
3.747
3.728
3.719
2.516
2.501
1.075
0.933
0.164
0.147
TBDPSO
O
N3
HO
OTBS
19
4.01
6.00
1.00
1.06
2.08
1.04
1.03
0.98
9.00
9.10
6.04
9.0
8.5
8.0
7.5
7.0
6.5
6.0
5.5
5.0
4.5
4.0
3.5
3.0
2.5
2.0
1.5
1.0
0.5
0.0
f1 (ppm)

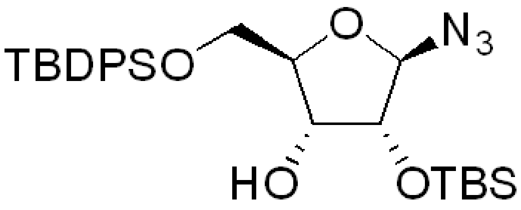
TBDPSO
O
N3
HO
OTBS

19

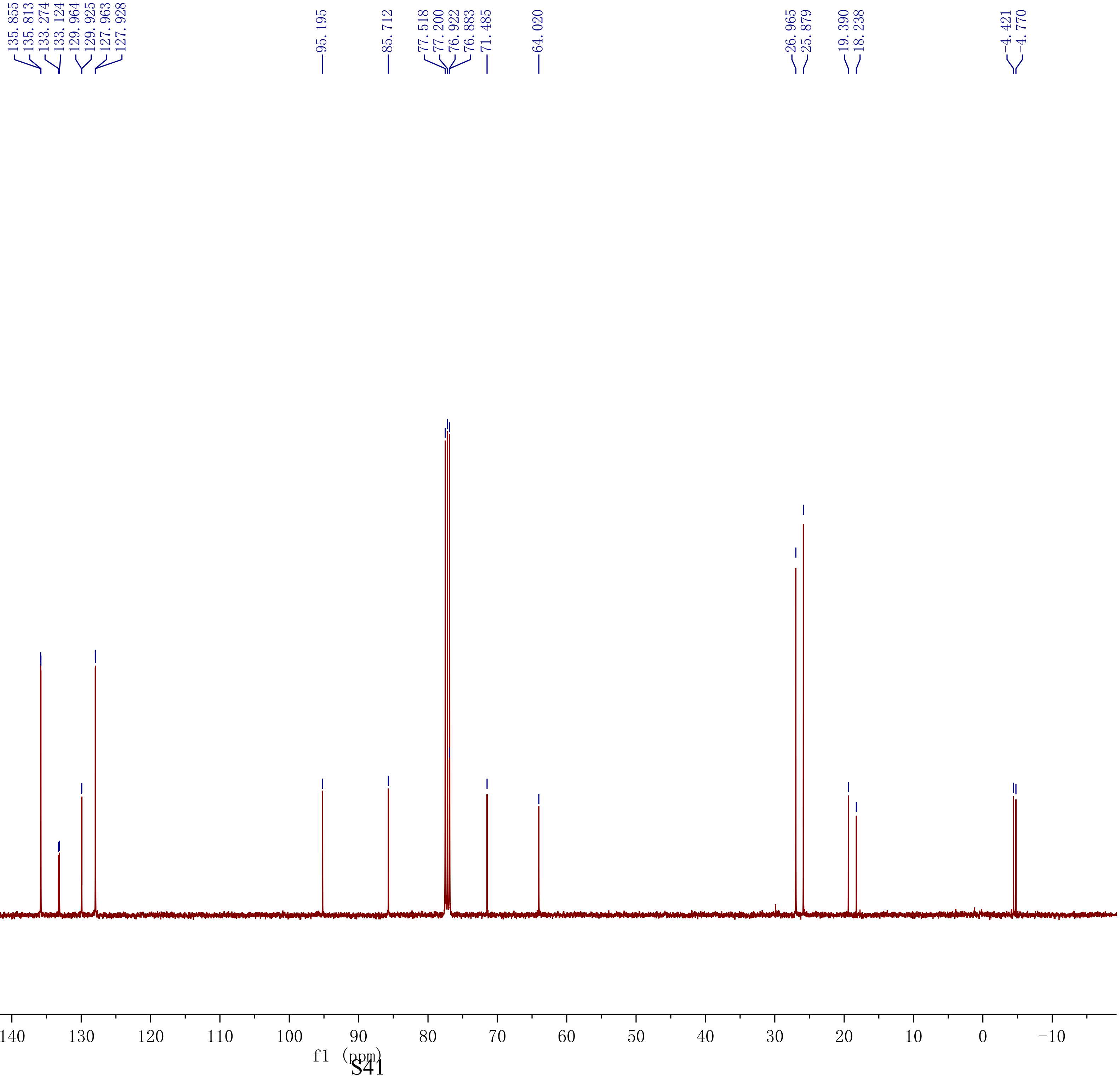
135.855
135.813
133.274
133.124
129.964
129.925
127.963
127.928
95.195
85.712
77.518
77.200
76.922
76.883
71.485
64.020
26.965
25.879
19.390
18.238
-4.421
-4.770
200 190 180 170 160 150 140 130 120 110 100 90 80 70 60 50 40 30 20 10 0 -10
f1 (ppm)

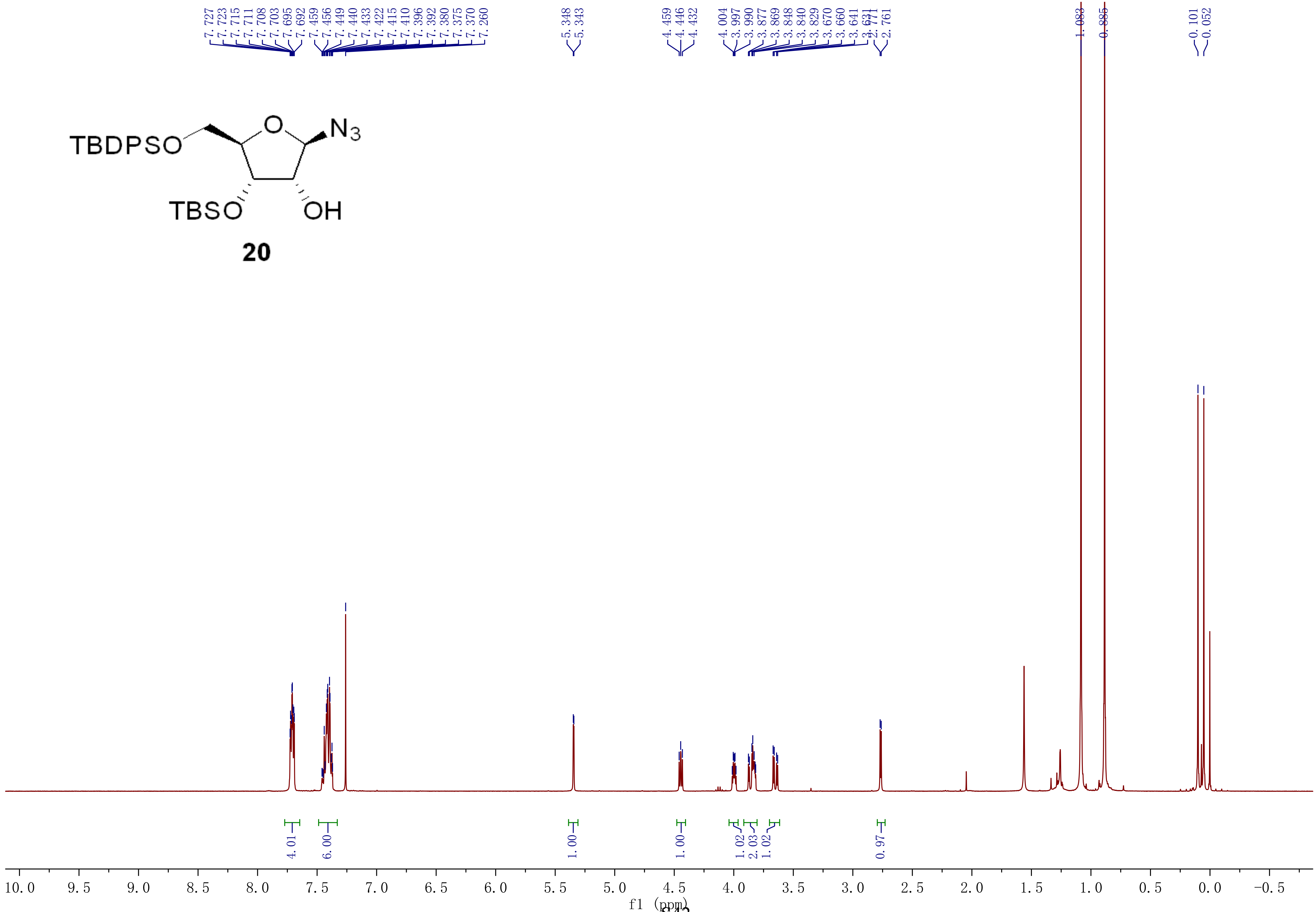
TBDPSO
N3
O
TBSO
OH
20
7.727
7.723
7.715
7.711
7.708
7.703
7.695
7.692
7.459
7.456
7.449
7.440
7.433
7.422
7.415
7.410
7.396
7.392
7.380
7.375
7.370
7.260
5.348
5.343
4.459
4.446
4.432
4.004
3.997
3.990
3.877
3.869
3.848
3.840
3.829
3.670
3.660
3.641
2.761
1.083
0.885
0.101
0.052
4.01
6.00
1.00
1.00
1.02
2.03
1.02
0.97
f1 (ppm)

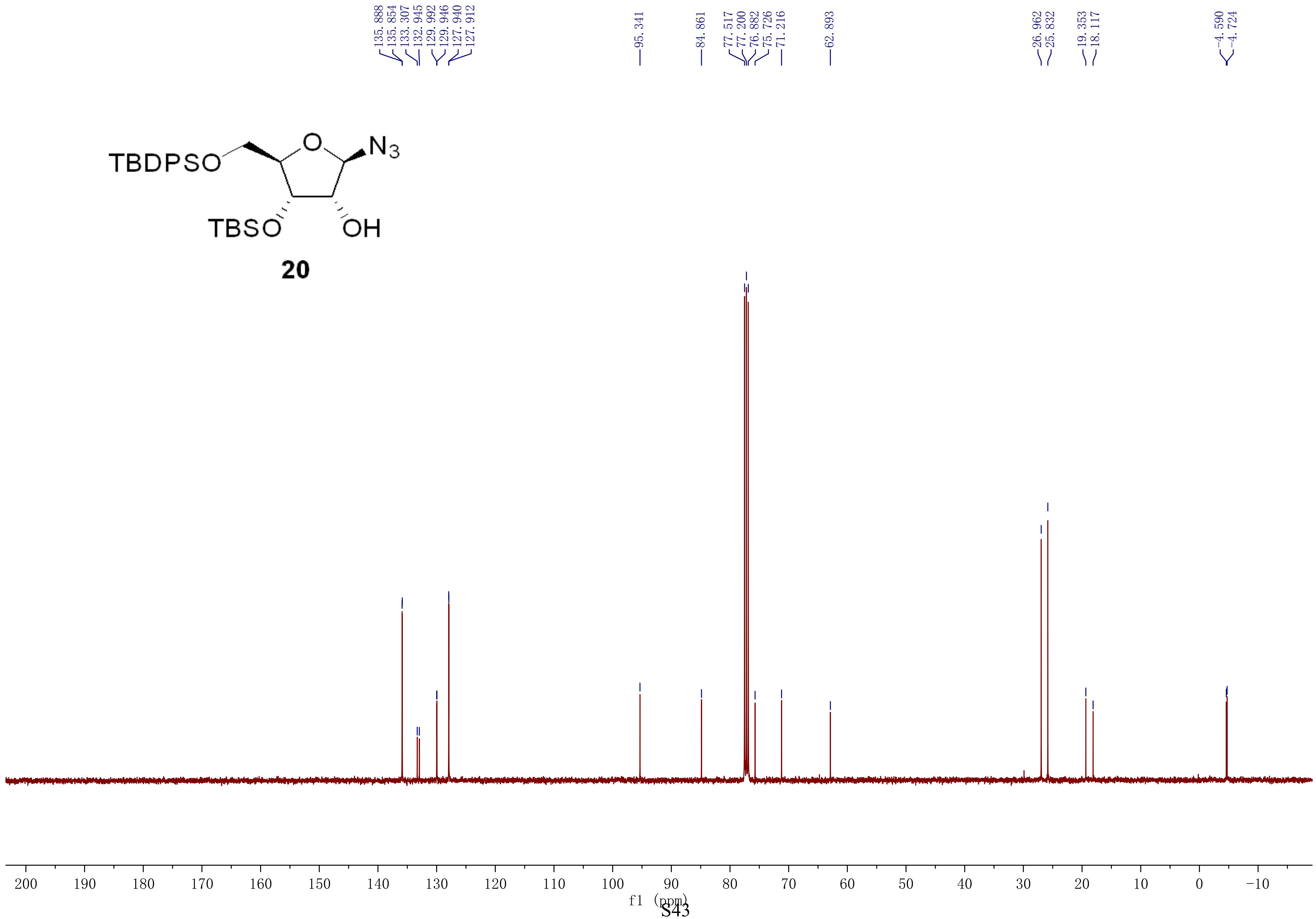
135.888
135.854
133.307
132.945
129.992
129.946
127.940
127.912
95.341
84.861
77.517
77.200
76.882
75.726
71.216
62.893
26.962
25.832
19.353
18.117
-4.590
-4.724
TBDPSO
N3
TBSO
OH
20
f1 (ppm)

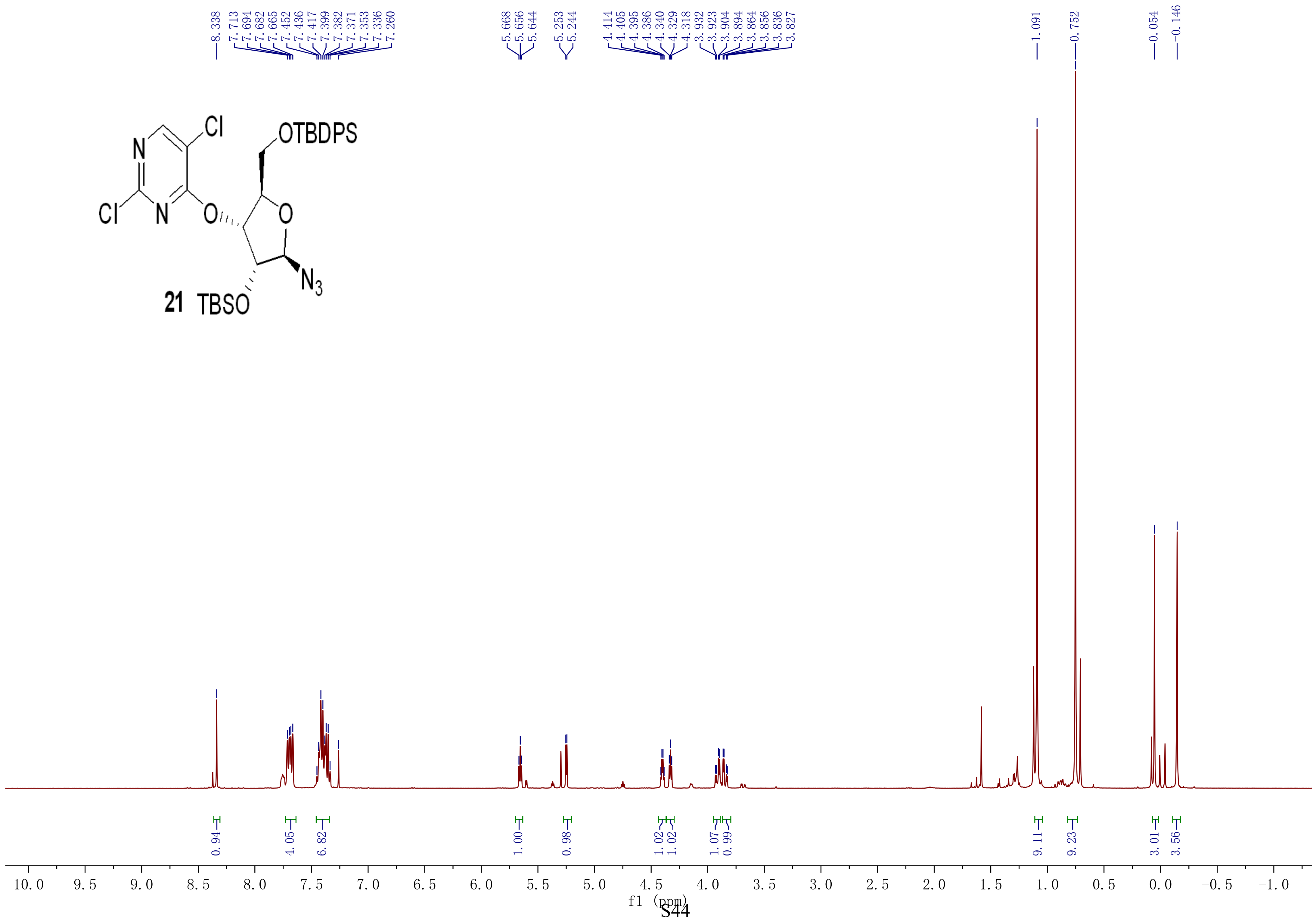
8.338
7.713
7.694
7.682
7.665
7.452
7.436
7.417
7.399
7.382
7.371
7.353
7.336
7.260
5.668
5.656
5.644
5.253
5.244
4.414
4.405
4.395
4.386
4.340
4.329
4.318
3.932
3.923
3.904
3.894
3.864
3.856
3.836
3.827
1.091
0.752
0.054
-0.146
OTBDPS
Cl
N
Cl
N
O
O
N3
21 TBSO
0.94
4.05
6.82
1.00
0.98
1.02
1.02
1.07
0.99
9.11
9.23
3.01
3.56
10.0 9.5 9.0 8.5 8.0 7.5 7.0 6.5 6.0 5.5 5.0 4.5 4.0 3.5 3.0 2.5 2.0 1.5 1.0 0.5 0.0 -0.5 -1.0
f1 (ppm)

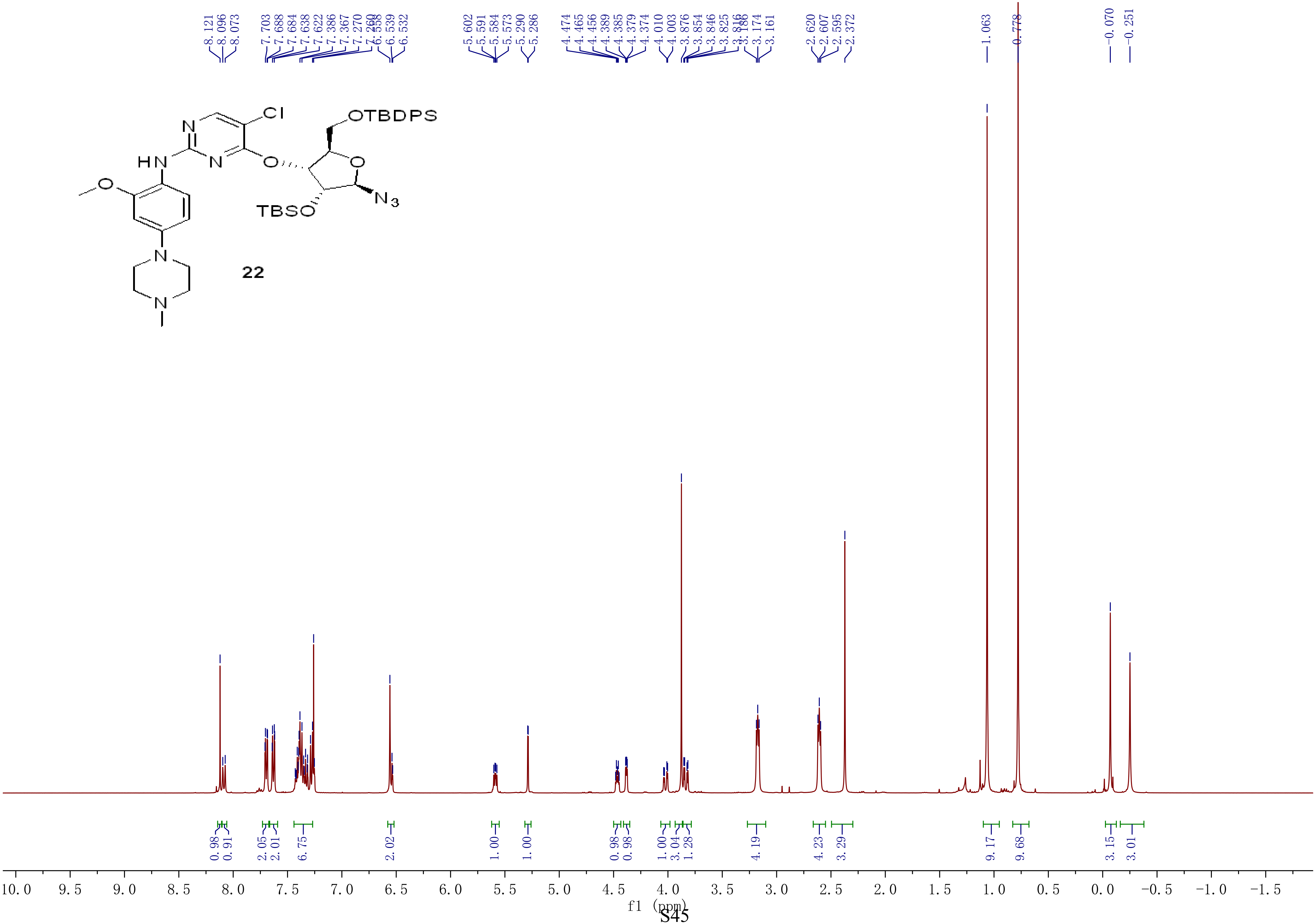

22

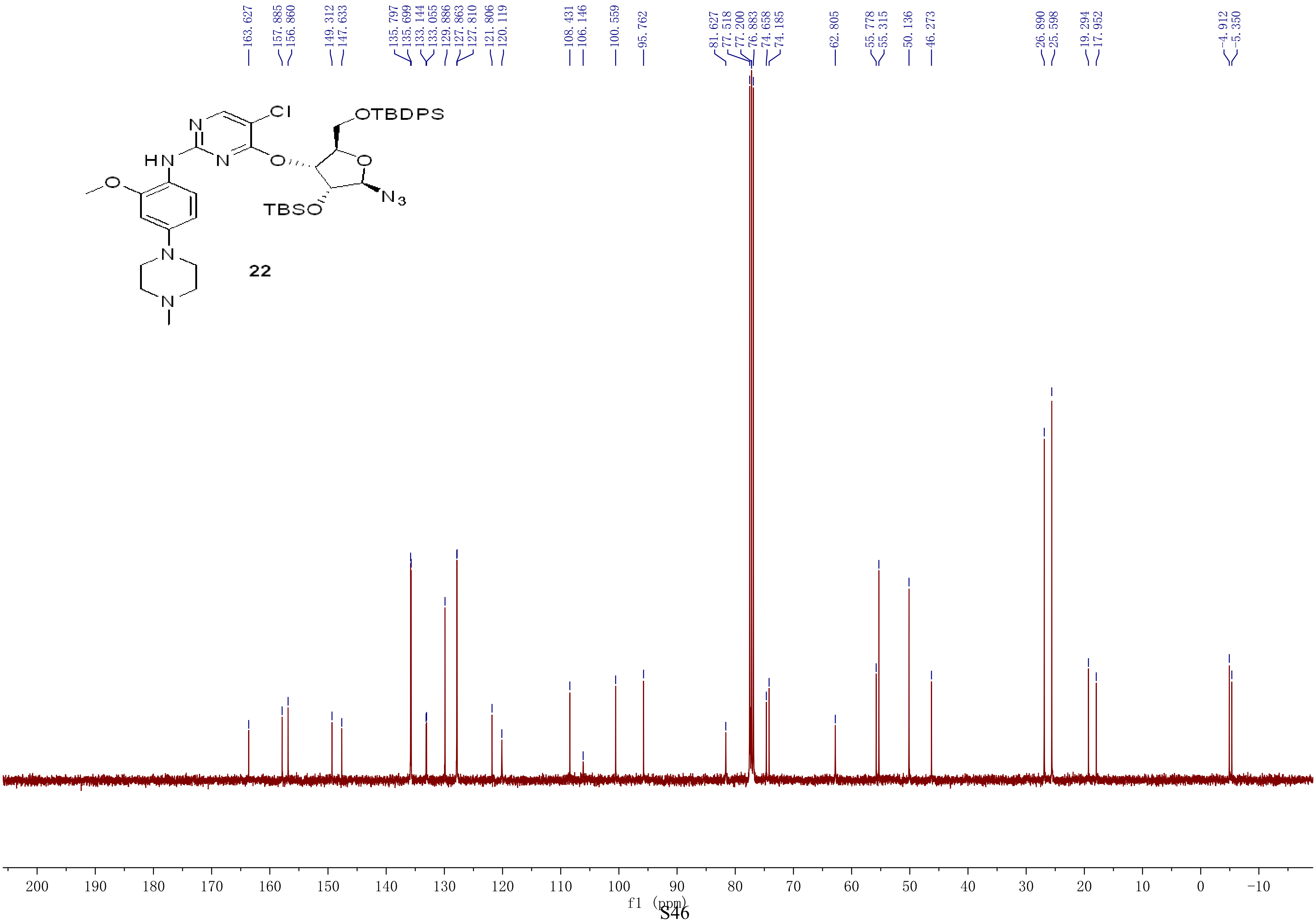

163.627
157.885
156.860
149.312
147.633
135.797
135.699
133.144
133.055
129.886
127.863
127.810
121.806
120.119
108.431
106.146
100.559
95.762
81.627
77.518
77.200
76.883
74.658
74.185
62.805
55.778
55.315
50.136
46.273
26.890
25.598
19.294
17.952
-4.912
-5.350
Cl
N
N
HN
O
OTBDPS
O
N3
TBSO
N
N
22
200 190 180 170 160 150 140 130 120 110 100 90 80 70 60 50 40 30 20 10 0 −10
f1 (ppm)

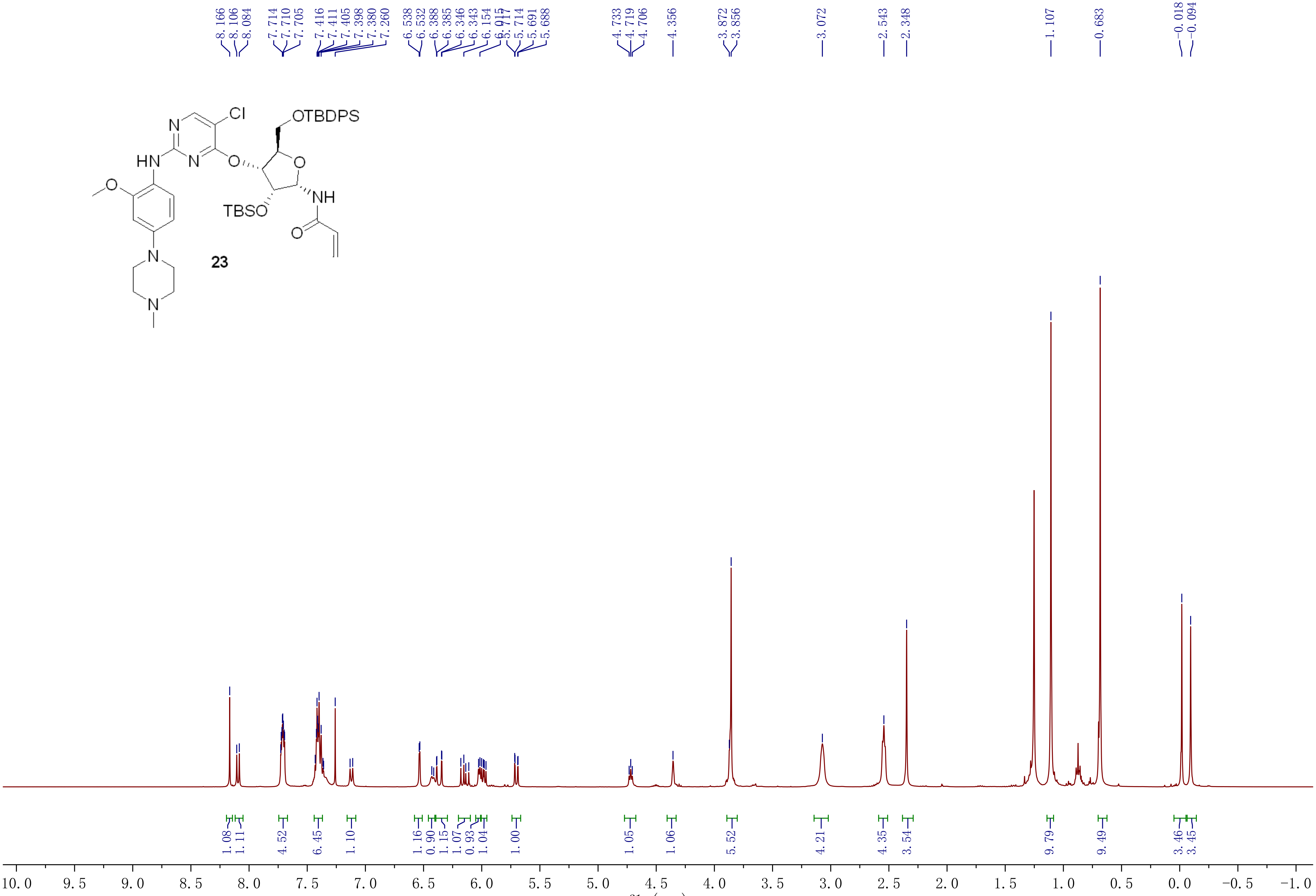

8.166
8.106
8.084
7.714
7.710
7.705
7.416
7.411
7.405
7.398
7.380
7.260
6.538
6.532
6.388
6.385
6.346
6.343
6.154
6.015
5.717
5.714
5.691
5.688
4.733
4.719
4.706
4.356
3.872
3.856
3.072
2.543
2.348
1.107
0.683
-0.018
-0.094
Cl
OTBDPS
N
HN
N
O
O
O
TBSO
NH
O
N
N
23
1.08
1.11
4.52
6.45
1.10
1.16
0.90
1.15
1.07
0.93
1.04
1.00
1.05
1.06
5.52
4.21
4.35
3.54
9.79
9.49
3.46
3.45
10.0 9.5 9.0 8.5 8.0 7.5 7.0 6.5 6.0 5.5 5.0 4.5 4.0 3.5 3.0 2.5 2.0 1.5 1.0 0.5 0.0 -0.5 -1.0
f1 (ppm)

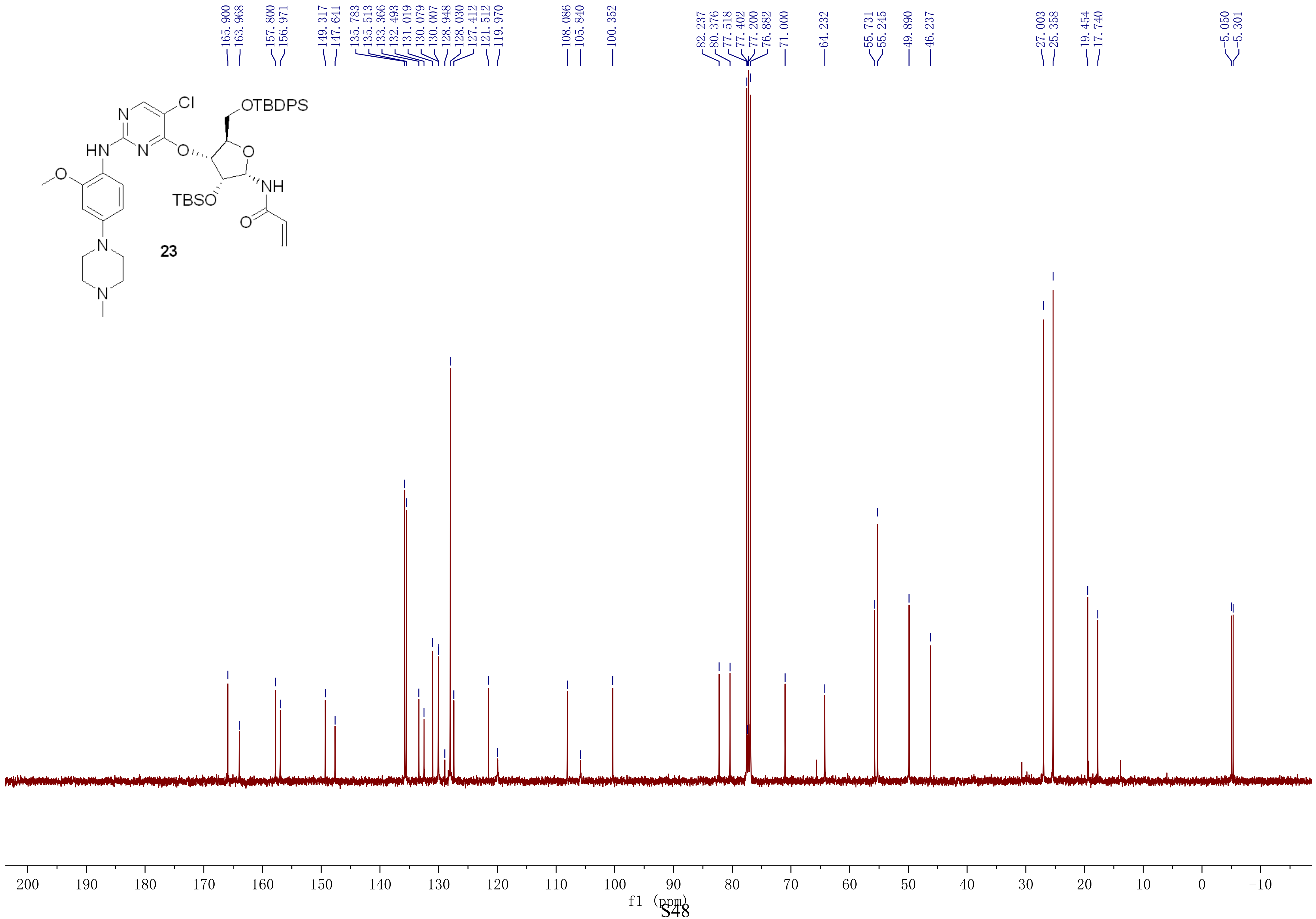
165.900
163.968
157.800
156.971
149.317
147.641
135.783
135.513
133.366
132.493
131.019
130.079
130.007
128.948
128.030
127.412
121.512
119.970
108.086
105.840
100.352
82.237
80.376
77.518
77.402
77.200
76.882
71.000
64.232
55.731
55.245
49.890
46.237
27.003
25.358
19.454
17.740
-5.050
-5.301
Cl
OTBDPS
N
HN
N
O
O
NH
TBSO
O
N
N
23
200
190
180
170
160
150
140
130
120
110
100
90
80
70
60
50
40
30
20
10
0
-10
f1 (ppm)

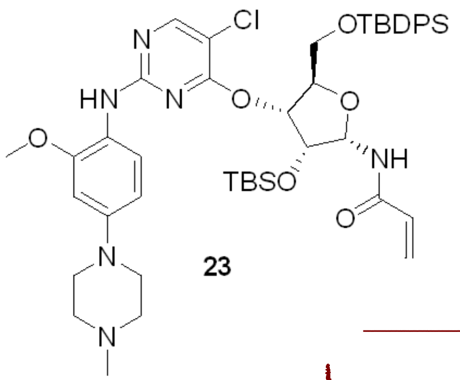

Cl
OTBDPS
N
HN
N
O
O
TBSO
NH
O
N
N

23

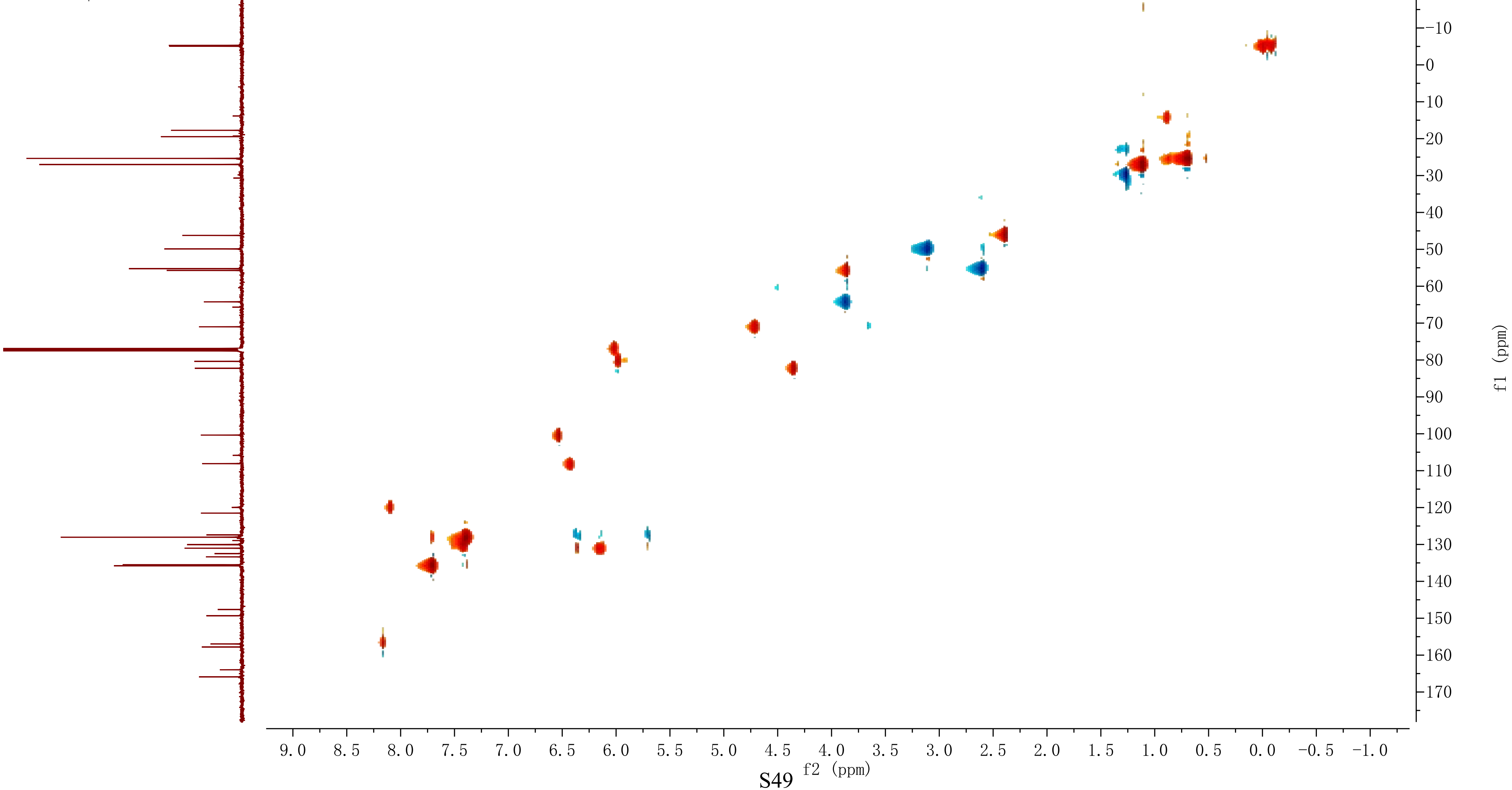

9.0
8.5
8.0
7.5
7.0
6.5
6.0
5.5
5.0
4.5
4.0
3.5
3.0
2.5
2.0
1.5
1.0
0.5
0.0
-0.5
-1.0
f2 (ppm)
-10
0
10
20
30
40
50
60
70
80
90
100
110
120
130
140
150
160
170
f1 (ppm)

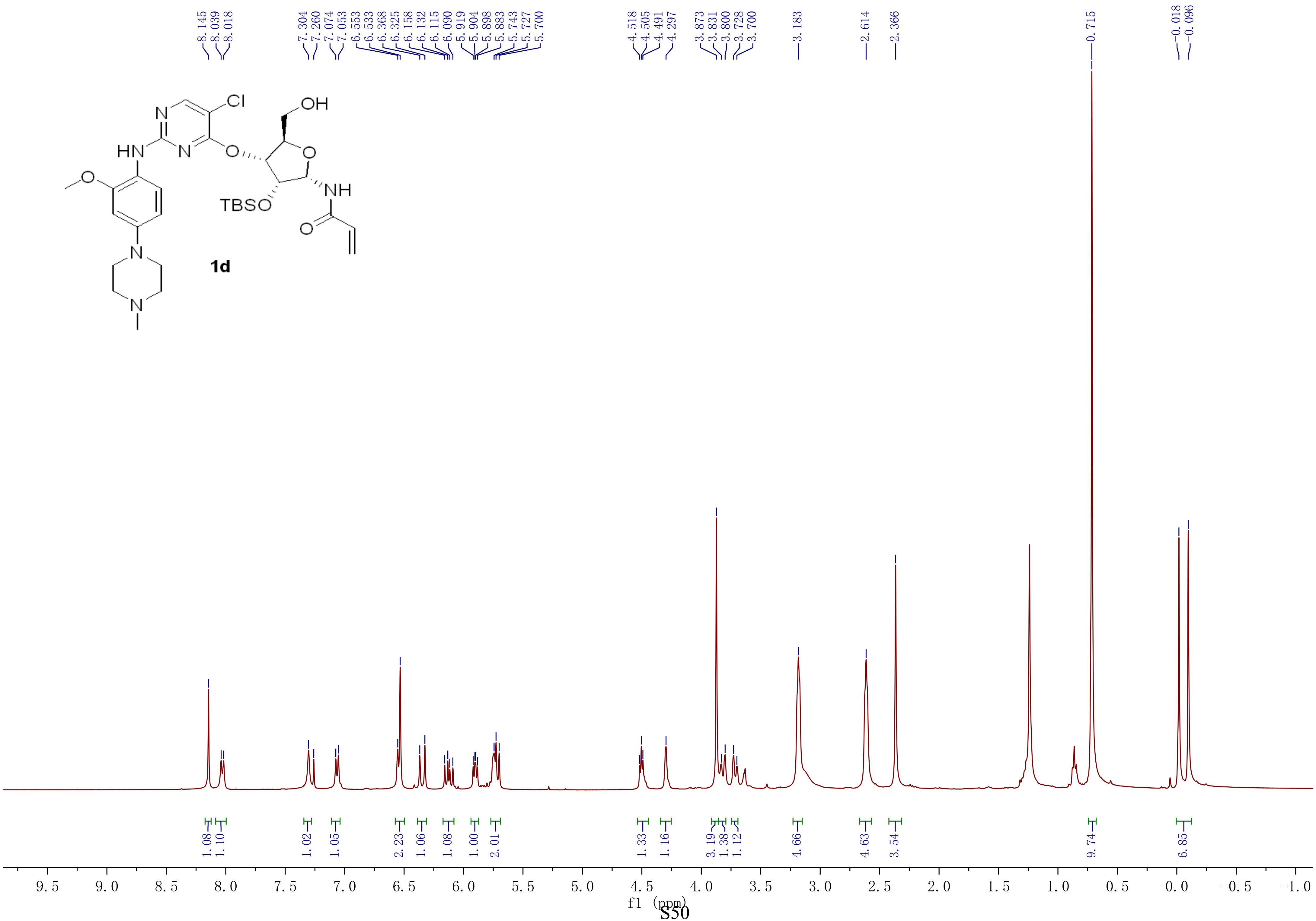
8.145
8.039
8.018
7.304
7.260
7.074
7.053
6.553
6.533
6.368
6.325
6.158
6.132
6.115
6.090
5.919
5.904
5.898
5.883
5.743
5.727
5.700
4.518
4.505
4.491
4.297
3.873
3.831
3.800
3.728
3.700
3.183
2.614
2.366
0.715
-0.018
-0.096
Cl
OH
N
HN
N
O
O
O
NH
TBSO
O
N
N
1d
1.08
1.10
1.02
1.05
2.23
1.06
1.08
1.00
2.01
1.33
1.16
3.19
1.38
1.12
4.66
4.63
3.54
9.74
6.85
9.5
9.0
8.5
8.0
7.5
7.0
6.5
6.0
5.5
5.0
4.5
4.0
3.5
3.0
2.5
2.0
1.5
1.0
0.5
0.0
-0.5
-1.0
f1 (ppm)

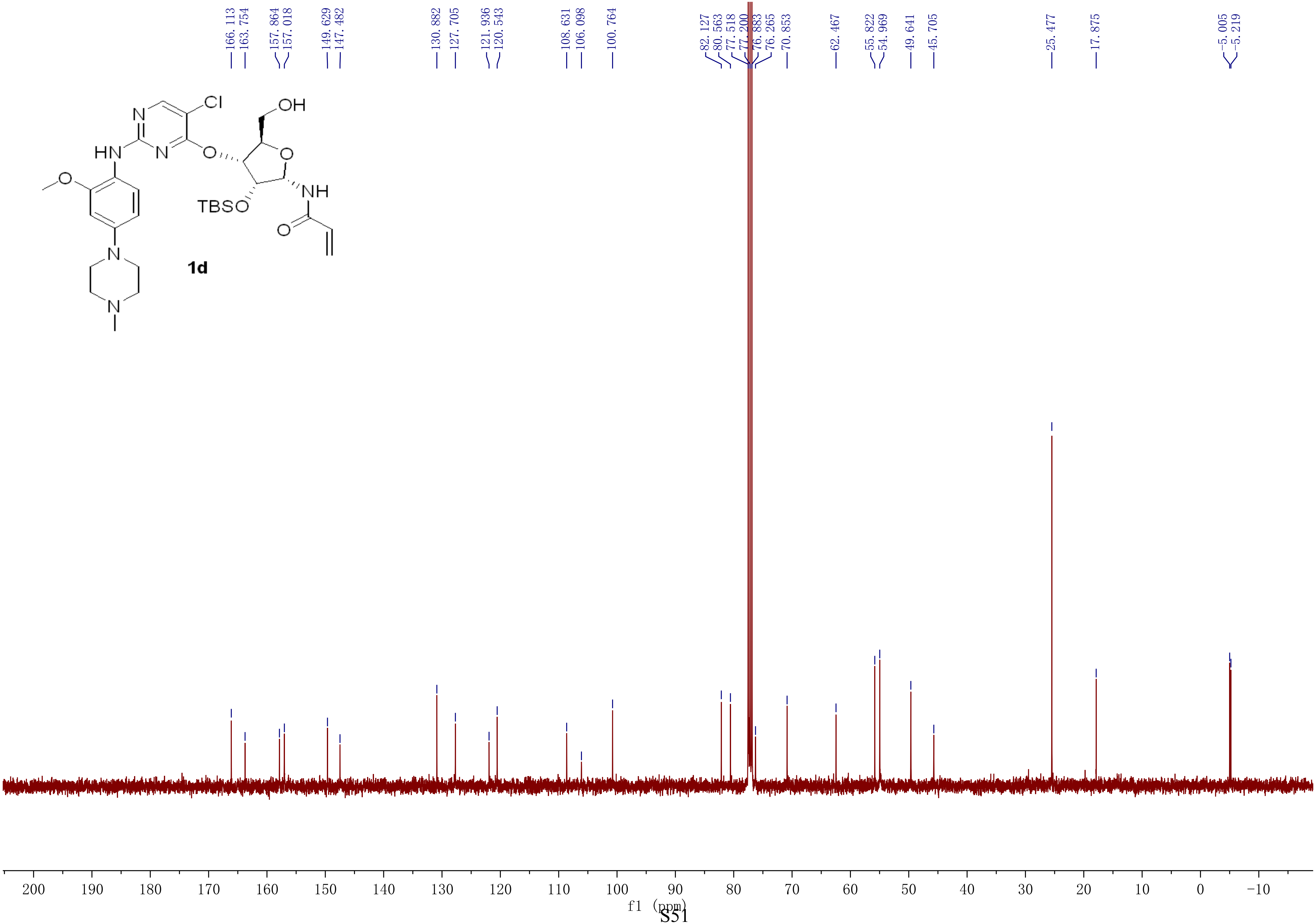

166.113
163.754
157.864
157.018
149.629
147.482
130.882
127.705
121.936
120.543
108.631
106.098
100.764
82.127
80.563
77.518
77.200
76.883
76.265
70.853
62.467
55.822
54.969
49.641
45.705
25.477
17.875
-5.005
-5.219
Cl
OH
N
HN
N
O
O
NH
TBSO
O
N
N
1d
200
190
180
170
160
150
140
130
120
110
100
90
80
70
60
50
40
30
20
10
0
-10
f1 (ppm)

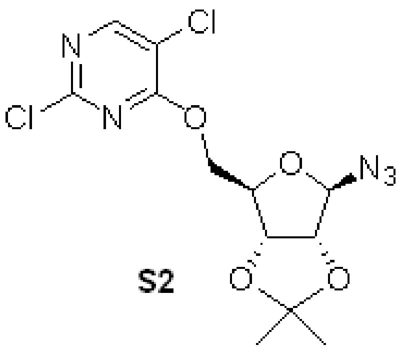

S2

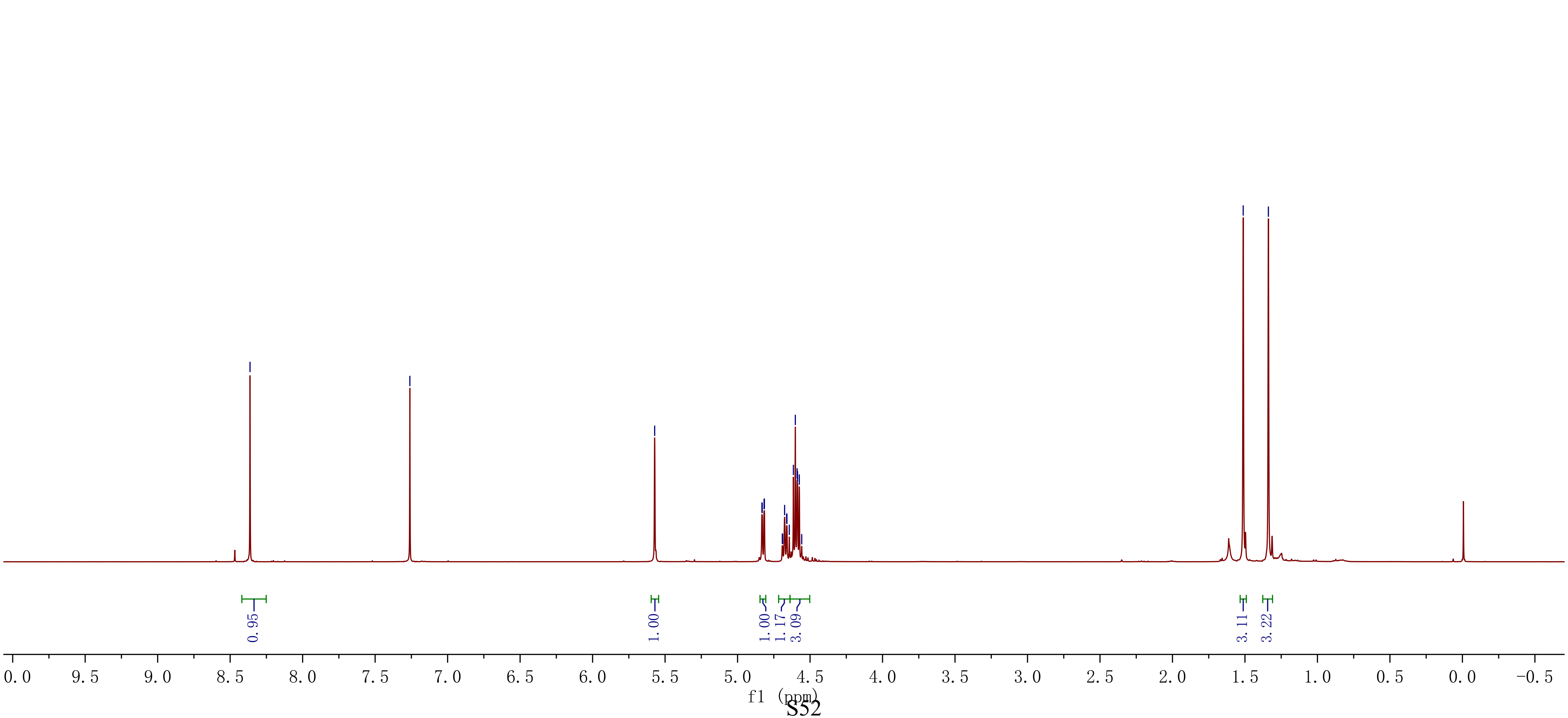

8.363
7.260
5.572
4.832
4.830
4.817
4.815
4.691
4.689
4.675
4.662
4.660
4.643
4.615
4.602
4.589
4.586
4.574
4.557
1.512
1.338
0.95
1.00
1.00
1.17
3.09
3.11
3.22
f1 (ppm)

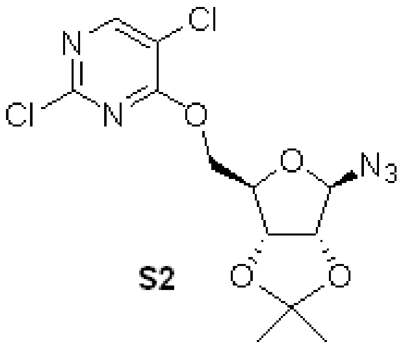

Cl
N
Cl
N
O
O
N3
O
O

S2

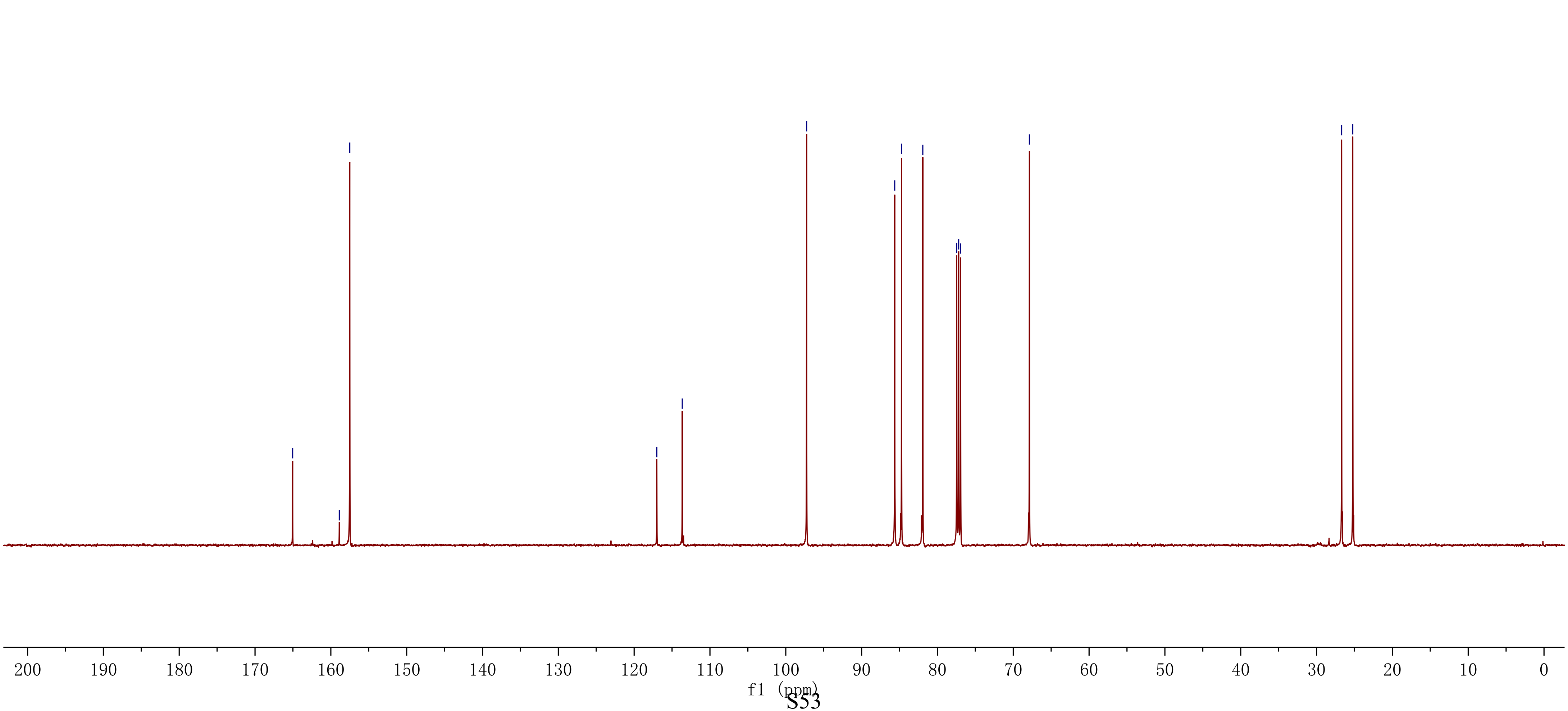

165.040
158.885
157.504
117.007
113.651
97.253
85.635
84.730
81.929
77.454
77.200
76.946
67.867
26.693
25.223
200 190 180 170 160 150 140 130 120 110 100 90 80 70 60 50 40 30 20 10 0
f1 (ppm)

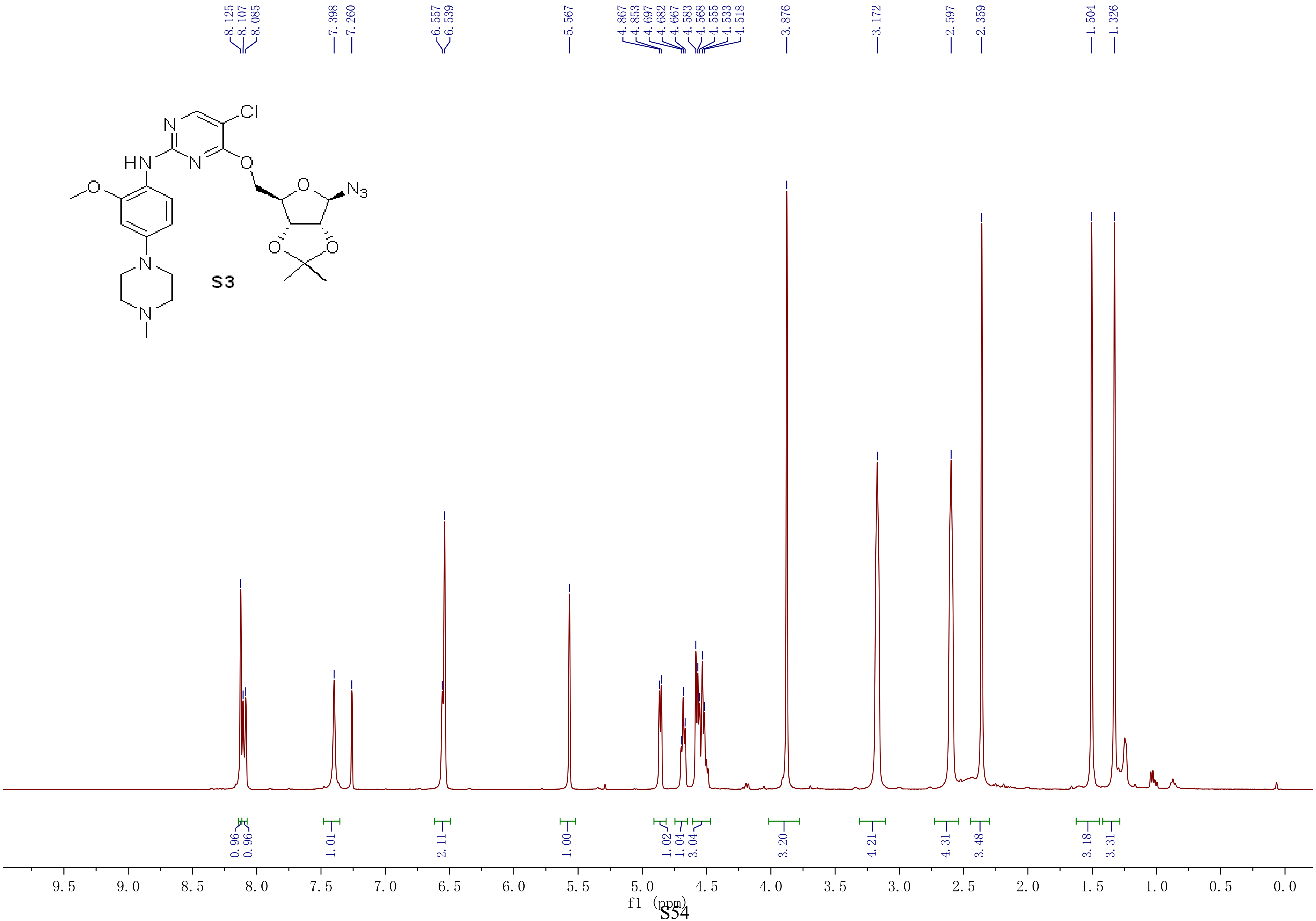
8.125
8.107
8.085
7.398
7.260
6.557
6.539
5.567
4.867
4.853
4.697
4.682
4.667
4.583
4.568
4.555
4.533
4.518
3.876
3.172
2.597
2.359
1.504
1.326
Cl
N
N
HN
O
O
O
N3
O
O
N
N
S3
0.96
0.96
1.01
2.11
1.00
1.02
1.04
3.04
3.20
4.21
4.31
3.48
3.18
3.31
9.5
9.0
8.5
8.0
7.5
7.0
6.5
6.0
5.5
5.0
4.5
4.0
3.5
3.0
2.5
2.0
1.5
1.0
0.5
0.0
f1 (ppm)

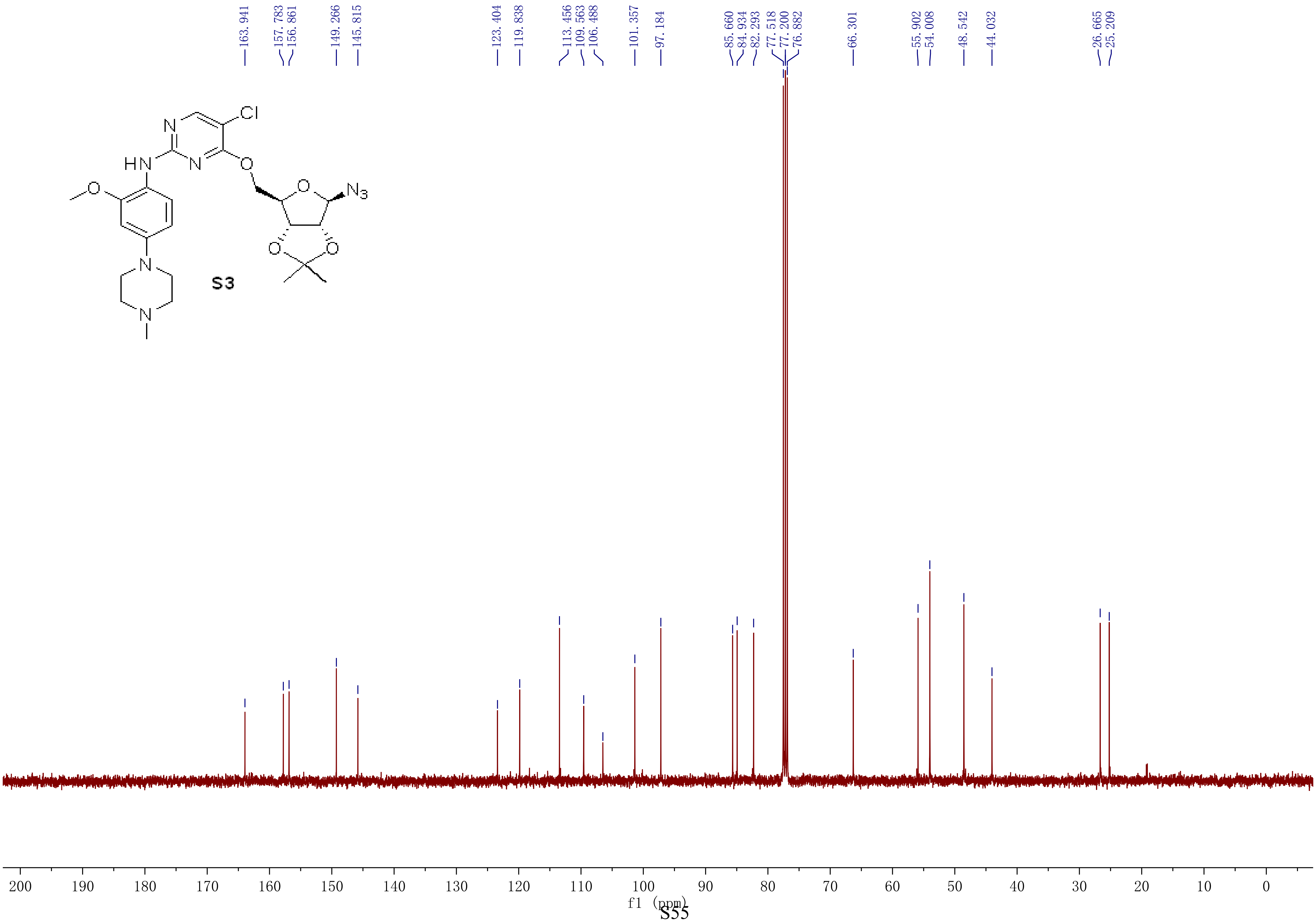
163.941
157.783
156.861
149.266
145.815
123.404
119.838
113.456
109.563
106.488
101.357
97.184
85.660
84.934
82.293
77.518
77.200
76.882
66.301
55.902
54.008
48.542
44.032
26.665
25.209
Cl
N
N
HN
O
O
O
N3
O
O
N
N
S3
200 190 180 170 160 150 140 130 120 110 100 90 80 70 60 50 40 30 20 10 0
f1 (ppm)

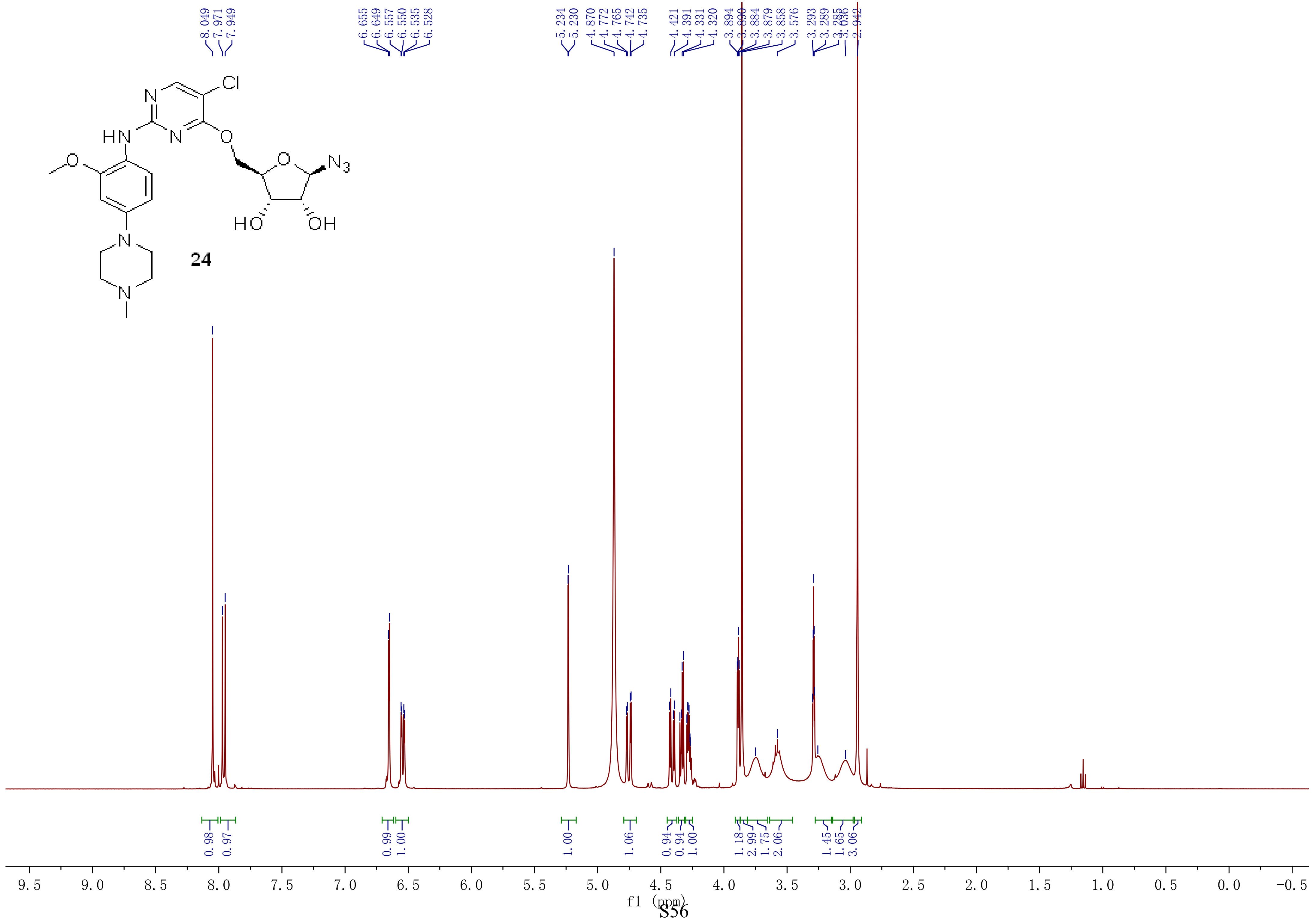
8.049
7.971
7.949
6.655
6.649
6.557
6.550
6.535
6.528
5.234
5.230
4.870
4.772
4.765
4.742
4.735
4.421
4.391
4.331
4.320
3.894
3.884
3.879
3.858
3.576
3.293
3.289
Cl
N
HN
N
O
O
N3
HO
OH
N
N
24
0.98
0.97
0.99
1.00
1.00
1.06
0.94
0.94
1.00
1.18
2.99
1.75
2.06
1.45
1.65
3.06
9.5
9.0
8.5
8.0
7.5
7.0
6.5
6.0
5.5
5.0
4.5
4.0
3.5
3.0
2.5
2.0
1.5
1.0
0.5
0.0
-0.5
f1 (ppm)

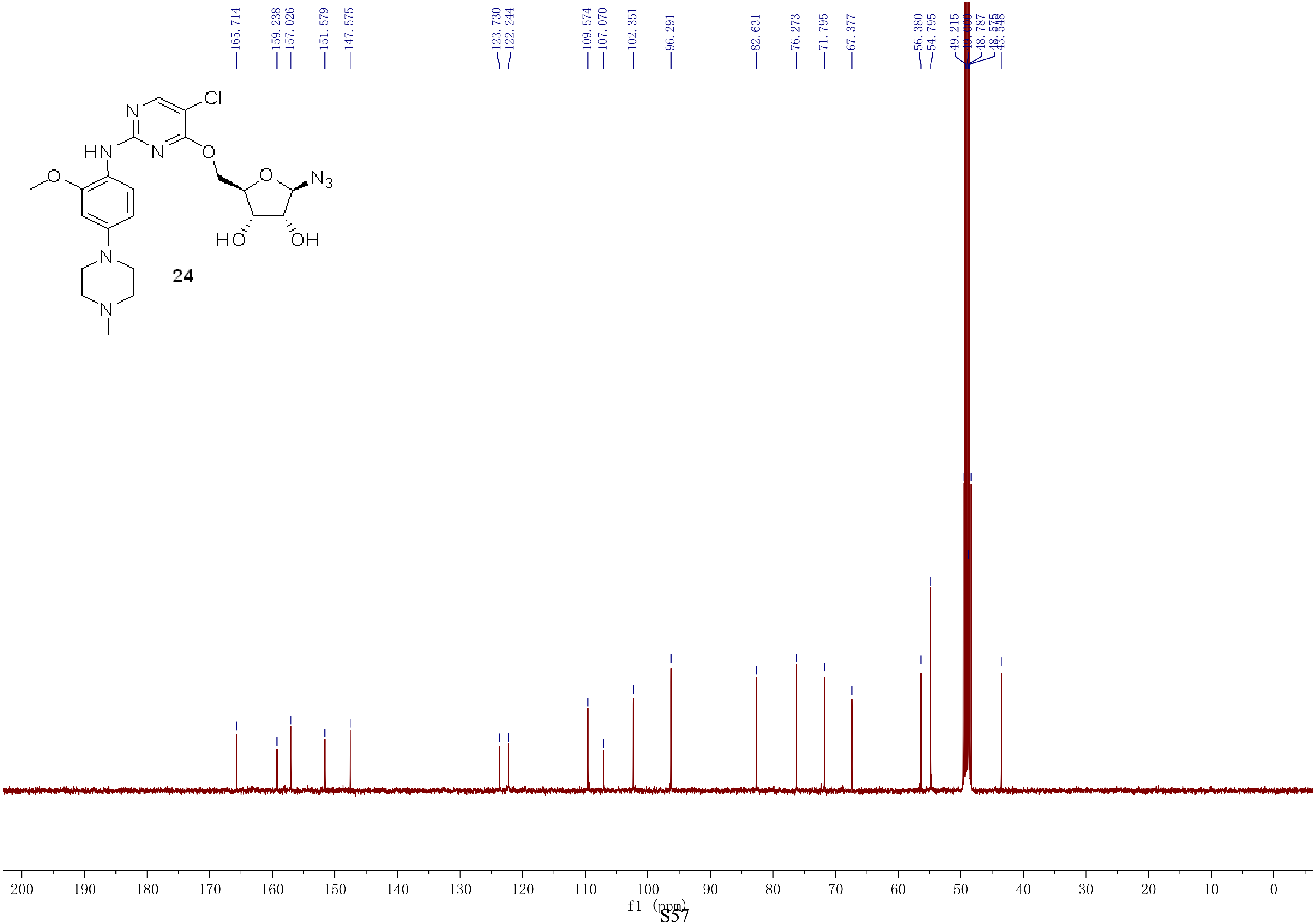
165.714
159.238
157.026
151.579
147.575
123.730
122.244
109.574
107.070
102.351
96.291
82.631
76.273
71.795
67.377
56.380
54.795
49.215
49.000
48.787
48.575
43.548
Cl
N
HN
N
O
O
O
N3
HO
OH
N
N
24
200
190
180
170
160
150
140
130
120
110
100
90
80
70
60
50
40
30
20
10
0
f1 (ppm)

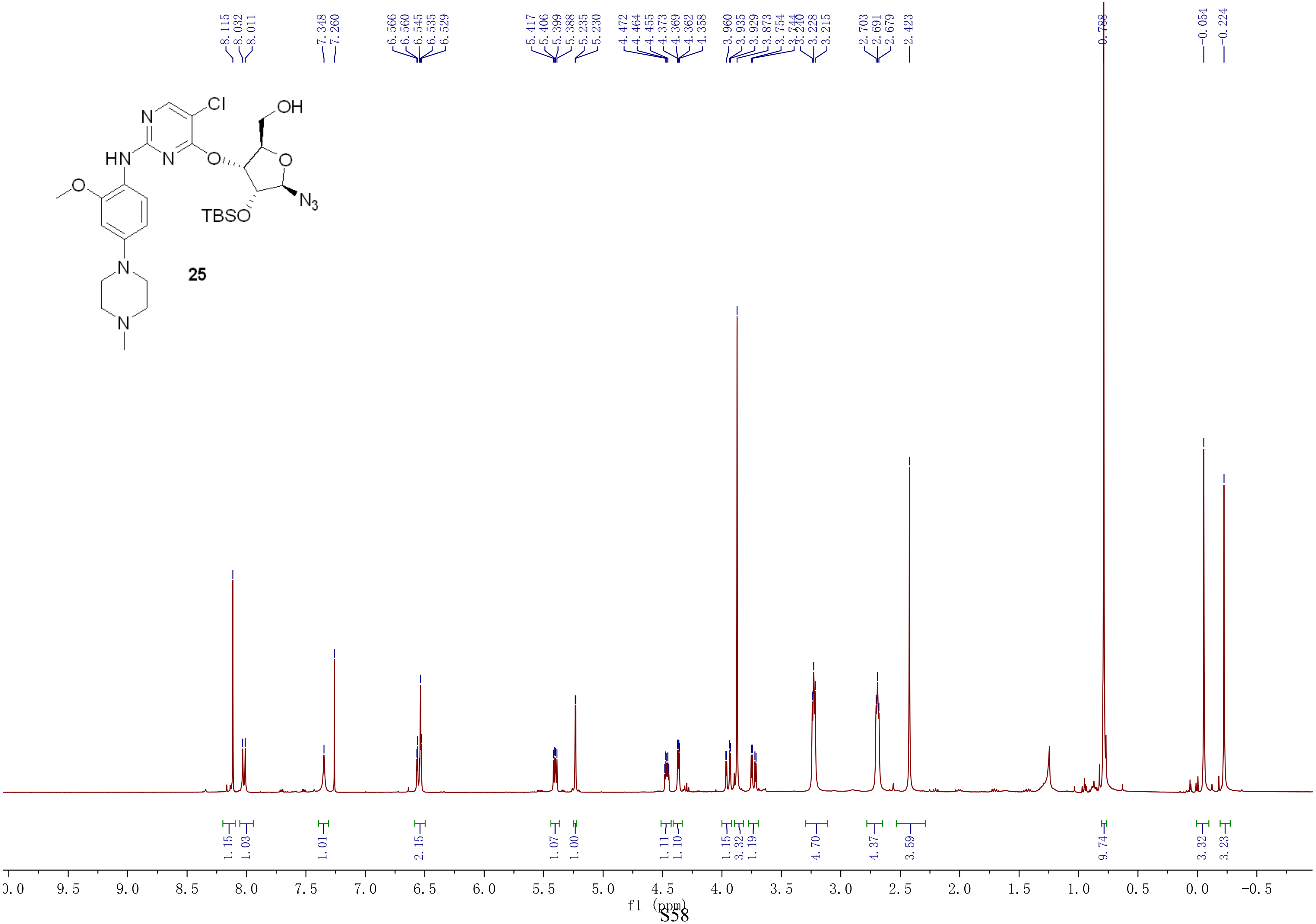
8.115
8.032
8.011
7.348
7.260
6.566
6.560
6.545
6.535
6.529
5.417
5.406
5.399
5.388
5.235
5.230
4.472
4.464
4.455
4.373
4.369
4.362
4.358
3.960
3.935
3.929
3.873
3.754
3.744
3.740
3.228
3.215
2.703
2.691
2.679
2.423
0.788
−0.054
−0.224
Cl
OH
N
HN
N
O
O
N3
TBSO
N
N
25
1.15
1.03
1.01
2.15
1.07
1.00
1.11
1.10
1.15
3.32
1.19
4.70
4.37
3.59
9.74
3.32
3.23
9.5
9.0
8.5
8.0
7.5
7.0
6.5
6.0
5.5
5.0
4.5
4.0
3.5
3.0
2.5
2.0
1.5
1.0
0.5
0.0
−0.5
f1 (ppm)

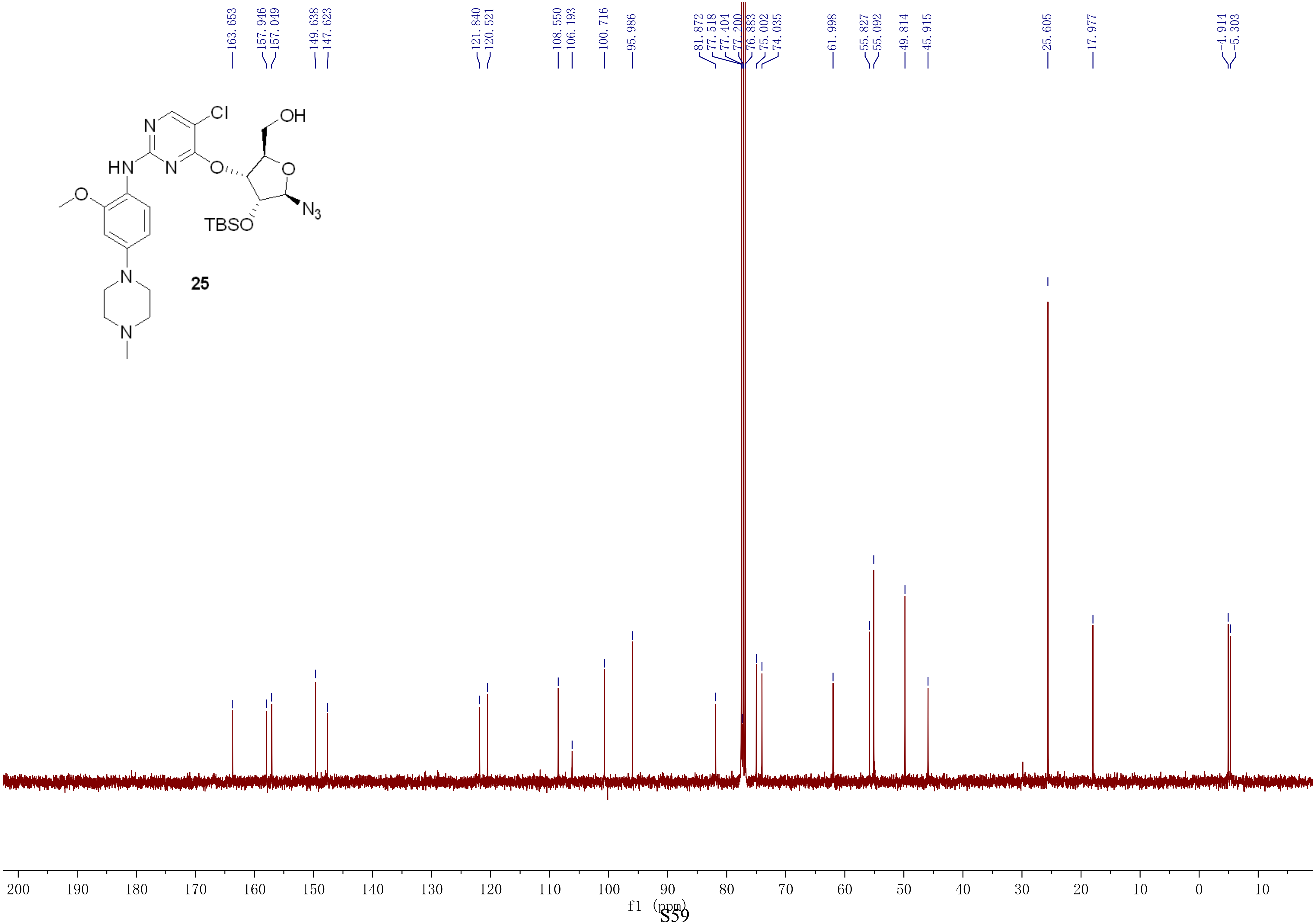
163.653
157.946
157.049
149.638
147.623
121.840
120.521
108.550
106.193
100.716
95.986
81.872
77.518
77.404
77.200
76.883
75.002
74.035
61.998
55.827
55.092
49.814
45.915
25.605
17.977
-4.914
-5.303
25
200 190 180 170 160 150 140 130 120 110 100 90 80 70 60 50 40 30 20 10 0 -10
f1 (ppm)

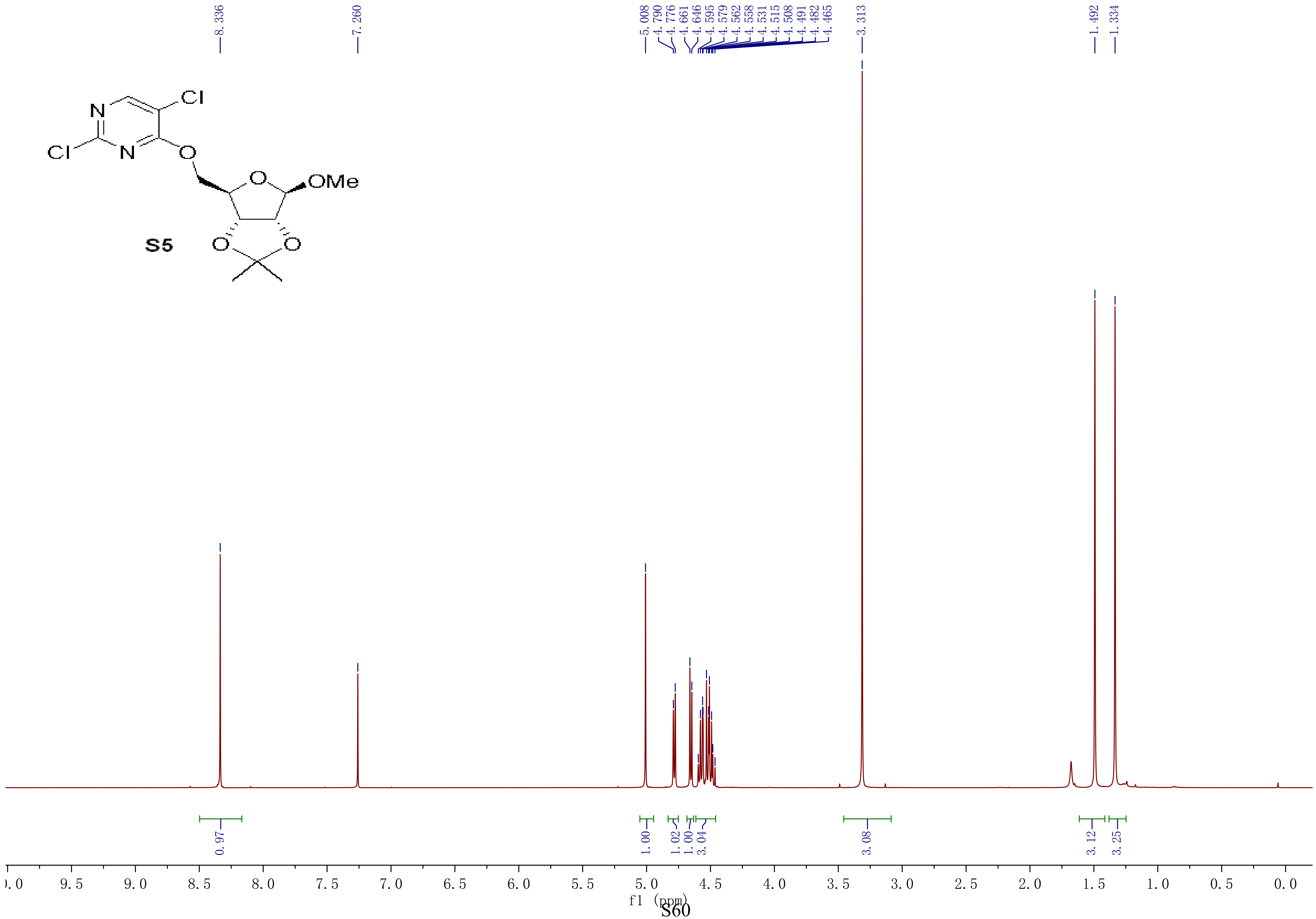
S5
OMe
Cl
N
O
8.336
7.260
5.008
4.790
4.776
4.661
4.646
4.595
4.579
4.562
4.558
4.531
4.515
4.508
4.491
4.482
4.465
3.313
1.492
1.334
0.97
1.00
1.02
1.00
3.04
3.08
3.12
3.25
f1 (ppm)

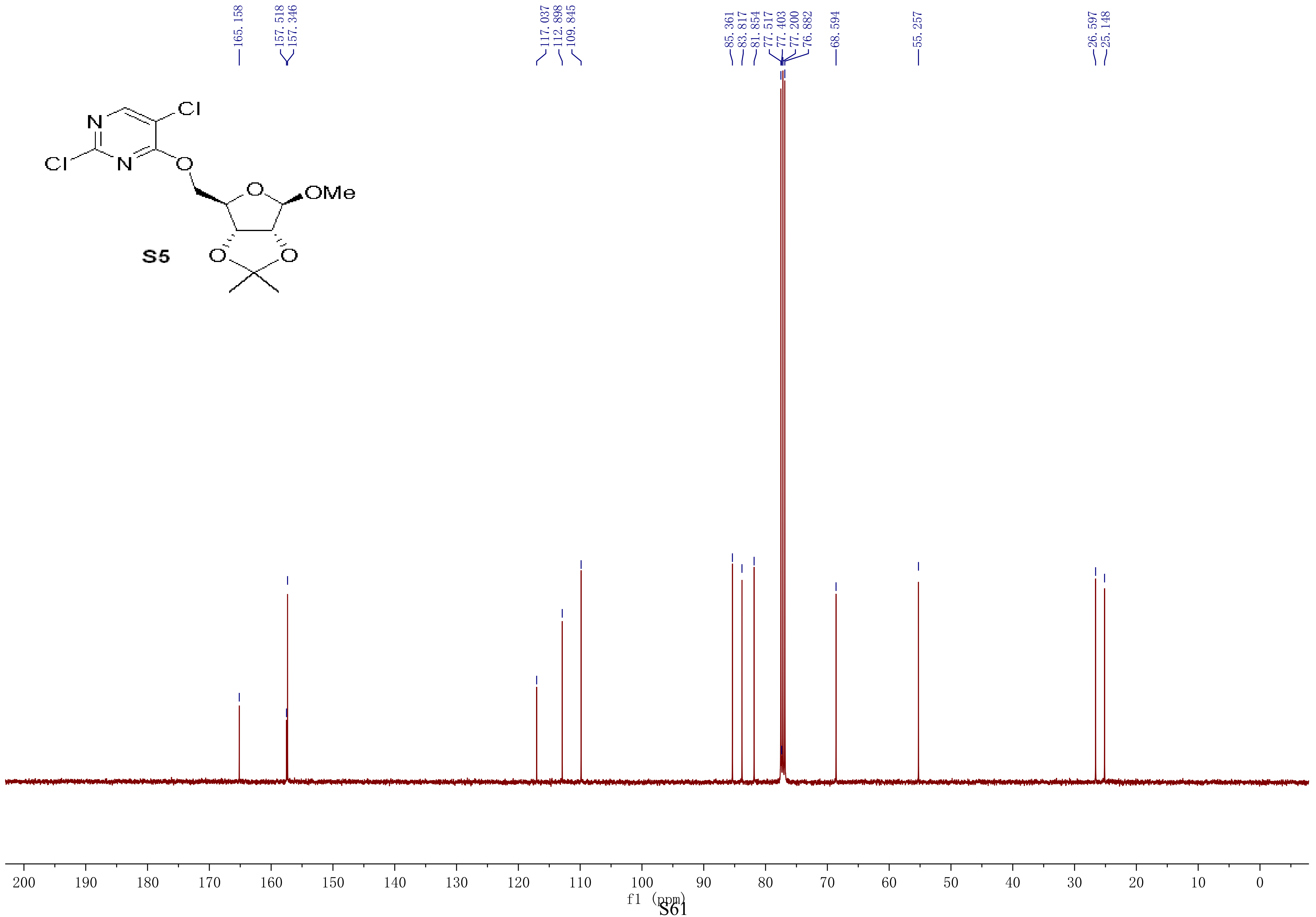
165.158
157.518
157.346
117.037
112.898
109.845
85.361
83.817
81.854
77.517
77.403
77.200
76.882
68.594
55.257
26.597
25.148
S5
OMe
200 190 180 170 160 150 140 130 120 110 100 90 80 70 60 50 40 30 20 10 0
f1 (ppm)

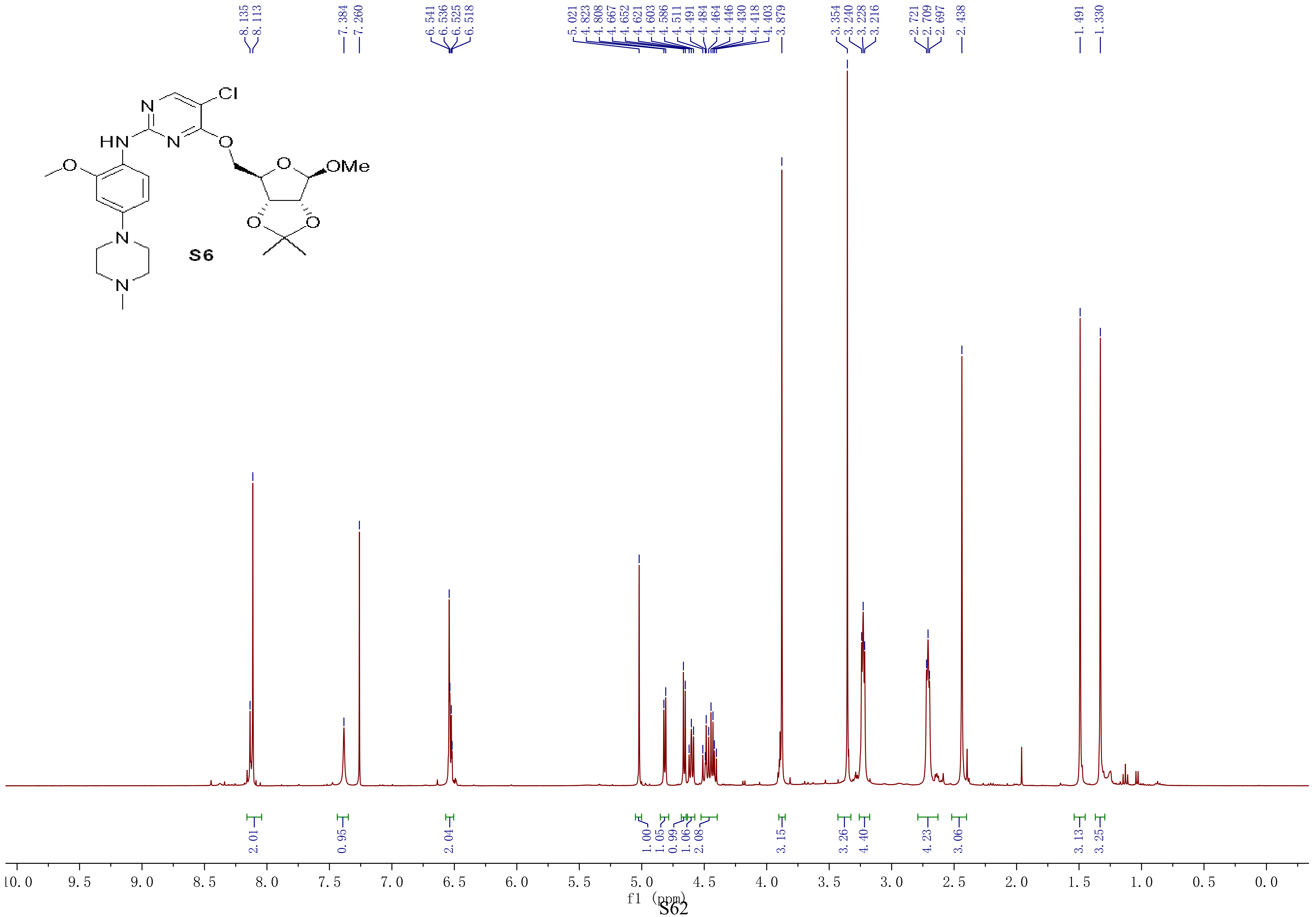
8.135
8.113
7.384
7.260
6.541
6.536
6.525
6.518
5.021
4.823
4.808
4.667
4.652
4.621
4.603
4.586
4.511
4.491
4.484
4.464
4.446
4.430
4.418
4.403
3.879
3.354
3.240
3.228
3.216
2.721
2.709
2.697
2.438
1.491
1.330
2.01
0.95
2.04
1.00
1.05
0.99
1.06
2.08
3.15
3.26
4.40
4.23
3.06
3.13
3.25
f1 (ppm)
Cl
N
HN
O
OMe
S6

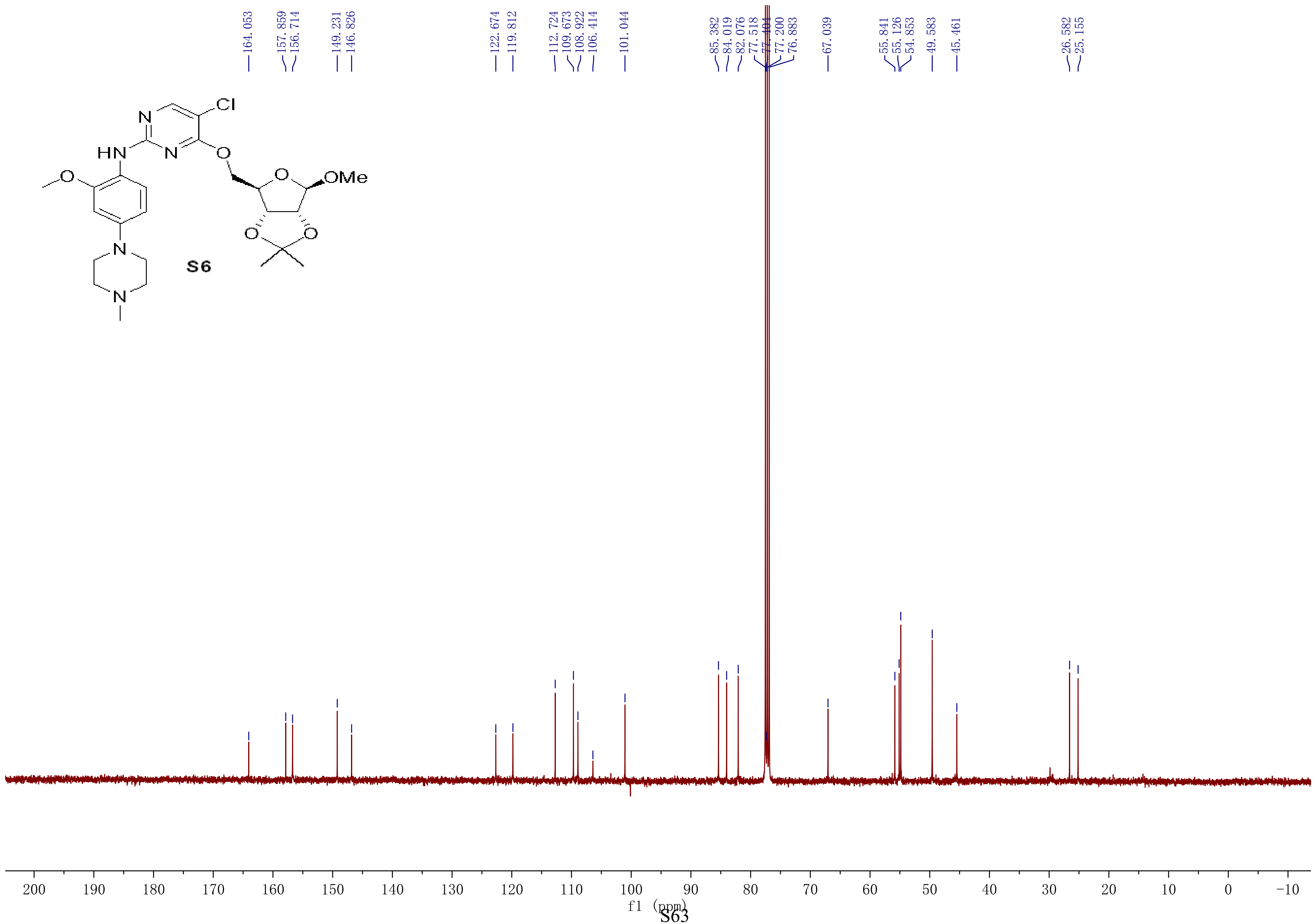
164.053
157.859
156.714
149.231
146.826
122.674
119.812
112.724
109.673
108.922
106.414
101.044
85.382
84.019
82.076
77.518
77.200
76.883
67.039
55.841
55.126
54.853
49.583
45.461
26.582
25.155
Cl
N
HN
N
O
OMe
O
O
O
N
N
S6
f1 (ppm)

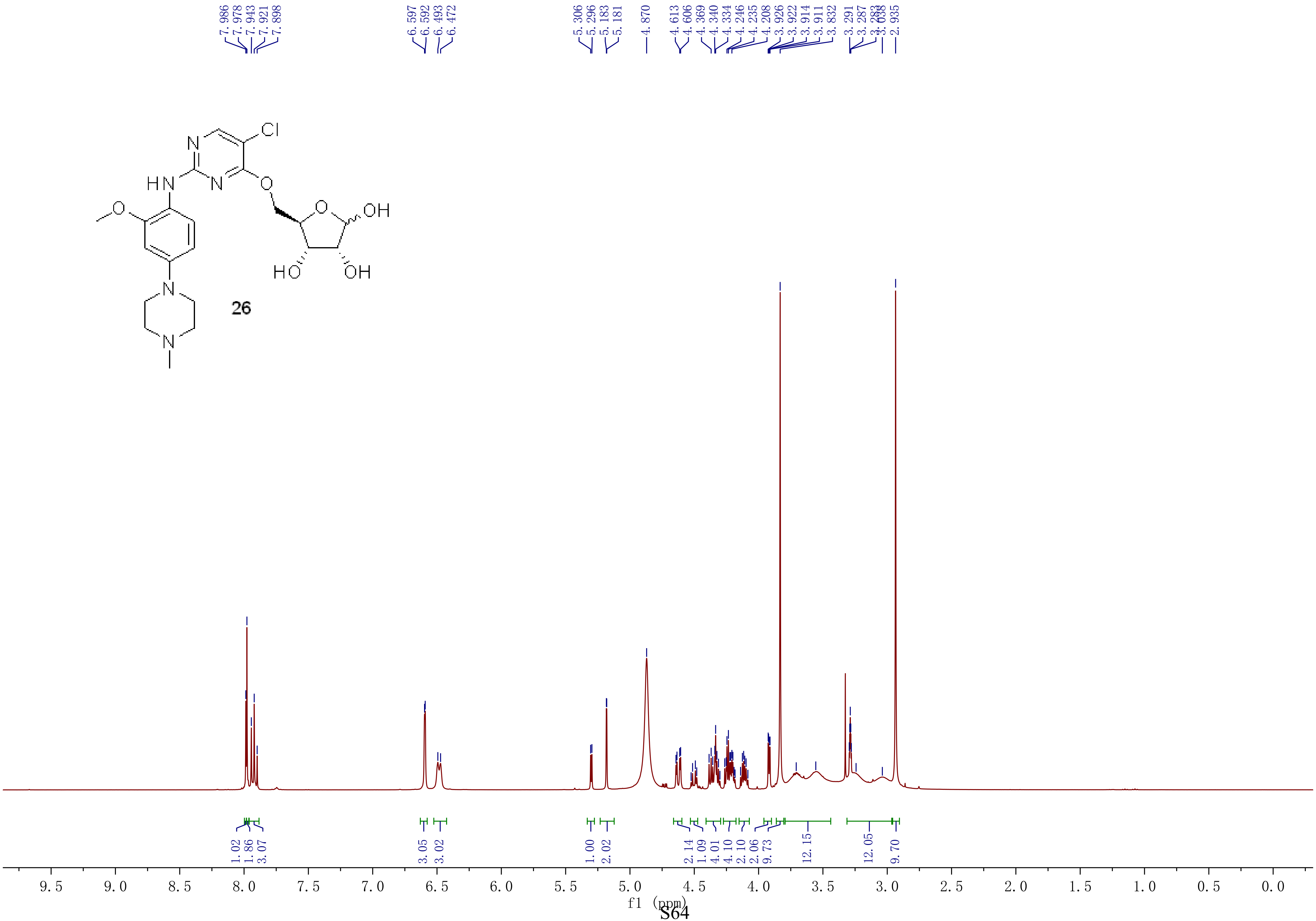
7.986
7.978
7.943
7.921
7.898
6.597
6.592
6.493
6.472
5.306
5.296
5.183
5.181
4.870
4.613
4.606
4.369
4.340
4.334
4.246
4.235
4.208
3.926
3.922
3.914
3.911
3.832
3.291
3.287
2.935
Cl
N
HN
N
O
O
O
OH
HO
OH
N
N
26
1.02
1.86
3.07
3.05
3.02
1.00
2.02
2.14
1.09
4.01
4.10
2.10
2.06
9.73
12.15
12.05
9.70
9.5
9.0
8.5
8.0
7.5
7.0
6.5
6.0
5.5
5.0
4.5
4.0
3.5
3.0
2.5
2.0
1.5
1.0
0.5
0.0
f1 (ppm)
